# EGFR activation disrupts immunotherapy response via SHP2-mediated suppression of tumor-intrinsic response to IFN-**γ**

**DOI:** 10.1172/JCI194377

**Published:** 2026-01-15

**Authors:** Wei-Tao Zhuang, Lan-Lan Pang, Li-Yang Hu, Jun Liao, Jian-Hua Zhan, Ting Li, Ri-Xin Chen, Jia-Ni Zheng, An-Lin Li, Wen-Yan Yu, Tian-Qin Mao, Liang Chen, Yu-Jian Huang, Shao-Dong Hong, Jing Li, Jun-Han Wu, Yi-Ming Zeng, Meng-Juan Yang, Hai-Qing Zeng, Ya-Xiong Zhang, Li Zhang, Wen-Feng Fang

**Affiliations:** 1 Department of Medical Oncology, State Key Laboratory of Oncology in South China, Guangdong Provincial Clinical Research Center for Cancer, Sun Yat-sen University Cancer Center, Guangzhou, China.; 2 State Key Laboratory of Respiratory Diseases, National Clinical Research Center for Respiratory Disease, National Center for Respiratory Medicine, Department of Pulmonary and Critical Care Medicine, Guangzhou Institute of Respiratory Health, The First Affiliated Hospital of Guangzhou Medical University, Guangzhou, China.; 3 Department of Thoracic Oncology, the Cancer Center of the Fifth Affiliated Hospital, Sun Yat-sen University, Zhuhai, China.; 4 Department of Thoracic Surgery, Guangdong Provincial People’s Hospital (Guangdong Academy of Medical Sciences), Southern Medical University, Guangzhou, China.; 5 Department of Medical Oncology, Xiamen Key Laboratory of Antitumor Drug Transformation Research, the First Affiliated Hospital of Xiamen University, School of Medicine, Xiamen University, Xiamen, China.; 6 MOE Key Laboratory of Tumor Molecular Biology and Key Laboratory of Functional Protein Research of Guangdong Higher Education Institutes, Institute of Life and Health Engineering, College of Life Science and Technology, Jinan University, Guangzhou, China.

**Keywords:** Immunology, Oncology, Cancer immunotherapy, Lung cancer, Oncogenes

## Abstract

Epidermal growth factor receptor–activating (EGFR-activating) mutations are established biomarkers of resistance to immune checkpoint blockade (ICB) in lung cancer, yet the precise molecular mechanism and effective therapeutic strategies remain elusive. In this study, we show that *EGFR* overexpression and amplification recapitulated the negative effect of *EGFR* driver mutations on the ICB response, indicating a proactive involvement of EGFR signaling in antagonizing the antitumor immune response. Functional studies unveiled that EGFR activation suppressed the cellular response to IFN-γ following ICB treatment across multiple cancer models. This impairment in IFN-γ responsiveness further limited the upregulation of T cell–recruiting chemokines and antigen presentation, resulting in reduced T cell infiltration and activation, ultimately undermining antitumor immunity. Mechanistically, EGFR promotes Src homology 2-containing protein tyrosine phosphatase 2 (SHP2) activation to accelerate STAT1 dephosphorylation, leading to premature termination of the IFN-γ response. SHP2 inhibition restored ICB sensitivity in EGFR-activated tumors, significantly reducing tumor burden while maintaining a favorable safety profile. Our findings suggest that the EGFR/SHP2 axis functions as a molecular brake to disrupt the initiation and amplification of the IFN-γ–mediated antitumor response during immunotherapy. This discovery unveils a potential avenue to overcome immunotherapy resistance in EGFR-driven tumors, particularly lung cancer, through SHP2-targeted combination strategies.

## Abstract 2

EGFR activation in tumor promotes SHP2-mediated STAT1 dephosphorylation, thereby suppressing tumor-intrinsic IFN-γ signaling and its downstream chemokines production and antigen presentation to confer immunotherapy resistance.

## Graphical abstract

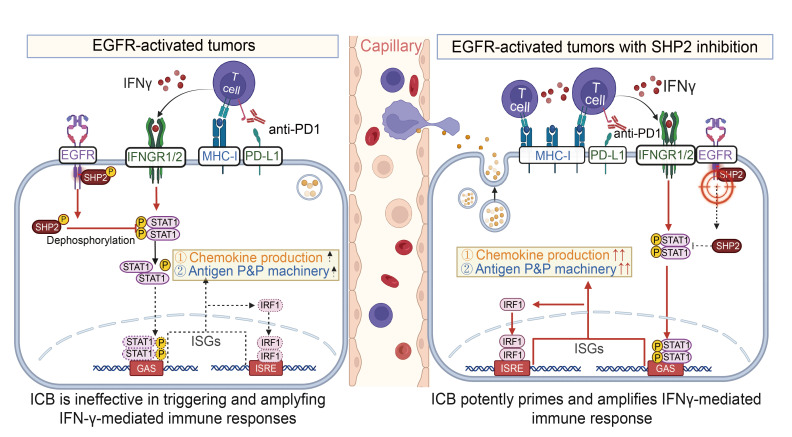

## Introduction

Immune checkpoint blockade (ICB) has revolutionized the treatment paradigm across a wide spectrum of tumor types, while its therapeutic benefit is usually limited to only a subset of patients (1). In contrast to patients who acquire resistance after achieving sustained tumor responses, those who derive no benefit from ICB treatment, a condition known as primary resistance, account for more than half of all treated patients (2–5). This signifies a substantial unmet clinical need and highlights emerging research priorities. In non–small cell lung cancer (NSCLC), epidermal growth factor receptor (EGFR) oncogenic mutation is well established as a genotype associated with primary resistance to ICB, as evidenced by the dismal treatment outcomes for patients with *EGFR-*mutant NSCLC in both retrospective analyses and prospective clinical trials (6–9). However, the current paucity of mechanistic insights into ICB resistance has precluded the establishment of viable therapeutic approaches. A substantial proportion of patients with lung adenocarcinoma (LUAD) — approximately of whom are 45% Asian and 15% White — are consequently excluded from receiving ICB-based therapies, resulting in a dire lack of effective treatments following frontline tyrosine kinase inhibitor (TKI) resistance (10). Therefore, deciphering the immune evasion mechanisms represents a crucial step toward developing tailored interventions to reclaim immunotherapy accessibility for this patient subset.

*EGFR*-mutant LUAD has reportedly been associated with low programmed death ligand 1 (PD-L1) expression levels, low tumor mutational burden (TMB), low tumor-infiltrating lymphocyte (TIL) numbers, and limited T cell receptor repertoires (11–14). Given these findings, *EGFR*-mutant NSCLCs are widely recognized as immunologically “cold” tumors, although the role of EGFR signaling in the formation of an immunosuppressive microenvironment is incompletely understood. Emerging evidence demonstrates that EGFR-targeted therapy dynamically remodels the tumor immune microenvironment (TIME) to a more inflamed phenotype compared with its pretreatment state, notably by increasing cytotoxic T cell infiltration (15, 16). This suggests that *EGFR* mutations may not simply serve as the enrichment biomarkers for cold tumors. Nevertheless, the precise molecular mechanism by which activated EGFR signaling drives ICB resistance remains poorly understood.

IFN-γ is predominantly secreted by activated T lymphocytes during immunotherapy, functioning as a pleiotropic immunoregulator that orchestrates multifaceted biological responses (17). IFN-γ signaling is associated with multiple antitumor effects (18), and the level of IFN-γ responsiveness represents a critical determinant of immunotherapy efficacy (19). Previous studies have indicated that tumor-intrinsic responsiveness to IFN-γ may vary as a result of perturbations by aberrant signaling networks of malignant cells (20, 21). However, the potential role of EGFR activation in conferring ICB resistance via modulation of the cellular response to IFN-γ remains to be elucidated.

In this study, we performed integrative in vivo and in vitro analyses using multiple murine and cell line models to unveil the role of the EGFR in immunoevasion and cross-validated our findings using proper clinical datasets. Utilizing in vivo studies and transcriptomics profiling, we identified the tumor-intrinsic response to IFN-γ as the leading candidate pathway dysregulated by EGFR activation during anti–programmed cell death protein 1 (anti–PD-1) treatment. We then illustrated how EGFR activation facilitates immunoevasion by suppressing IFN-γ–mediated chemokine production and antigen presentation. Further mechanistic investigation unveiled a Src homology 2–containing protein tyrosine phosphatase 2–dependent (SHP2-dependent) dampening of cellular responsiveness to IFN-γ in the context of EGFR activation. These findings provide mechanistic insight for developing combination therapy to overcome EGFR-driven immunoevasion and advocate for their evaluation in future clinical trials.

## Results

### EGFR activation diminishes antitumor efficacy of ICB by impairing the tumor-intrinsic response to IFN-γ.

**Figure 1:**
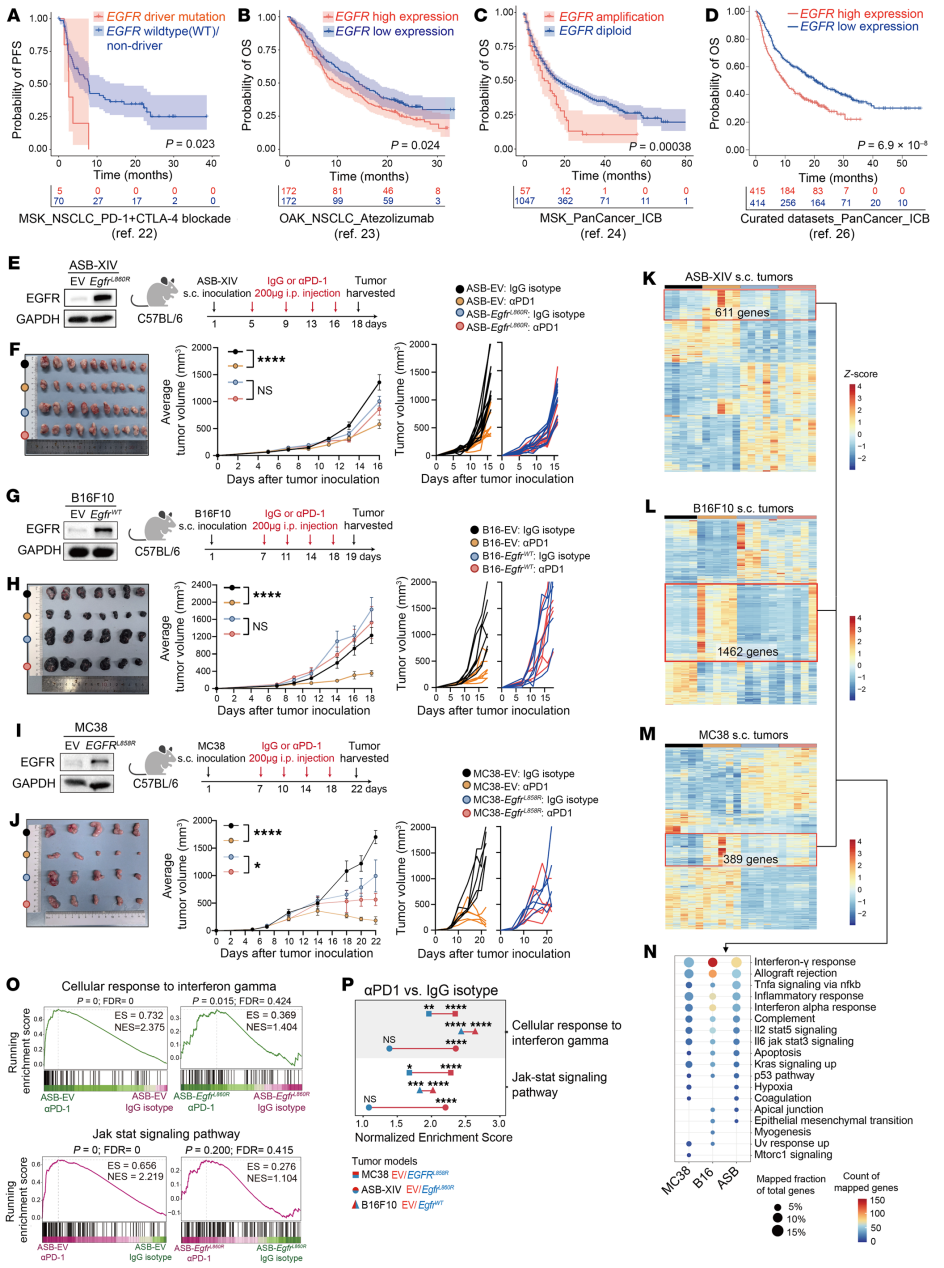
*EGFR* mutation, amplification, and overexpression diminish the antitumor efficacy of ICB by impairing the cellular response to IFN-γ. (**A**) Progression-free survival (PFS) of patients with NSCLC receiving combined PD-1 and CTLA-4 blockade, categorized by *EGFR* mutation status. (**B**) Overall survival (OS) of patients with NSCLC receiving atezolizumab, categorized by *EGFR* mRNA expression levels. (**C** and **D**) OS of pan-cancer patient cohorts receiving ICB, categorized by *EGFR* copy number variation (**C**) or *EGFR* mRNA expression (**D**). (**E** and **F**) Tumor growth curves of subcutaneous ASB-XIV lung cancer models stably transfected with EV or mouse *Egfr^L860R^* and treated with anti–PD-1 (αPD-1) or IgG control (*n* = 10 mice/group). (**G** and **H**) Tumor growth curves of subcutaneous B16F10 melanoma models stably transfected with EV or mouse WT *Egfr* and treated with anti–PD-1 or IgG control (*n* = 6–7 mice/group). (**I** and **J**) Growth curves of subcutaneous MC38 colon tumor models stably transfected with EV or human *EGFR^L858R^* and treated with anti–PD-1 or IgG control (*n* = 5 mice/group). (**K**–**M**) Heatmap of significantly differentially expressed genes in subcutaneous tumors derived from ASB-XIV-EV/*Egfr^L860R^* (**K**), B16F10-EV/*Egfr^WT^* (**L**), or MC38-EV/*EGFR^L858R^* (**M**) models treated with anti–PD-1 antibody or IgG control. (**N**) Top 15 of 50 MSigDB mouse ortholog hallmark gene sets enriched from genes that showed pattern of significant upregulation in EV models but not in ectopic *Egfr/EGFR*-expressing models upon anti–PD-1 treatment. (**O**) GSEA of the transcriptome of ASB-XIV-EV (left) or ASB-XIV-*Egfr^L860R^* (right) models treated with anti–PD-1 antibody or IgG control. (**P**) Summarized paired GSEA results for 3 different tumor models (related to **O**). Data are presented as the mean ± SEM. *FDR *q* < 0.05, **FDR *q* < 0.01, ***FDR *q* < 0.001, ****FDR *q* < 0.0001. Significance was determined by log-rank test (**A**–**D**) and 2-way ANOVA with Tukey’s test (**F**, **H**, and **J**).

To assess the role of the EGFR in ICB treatment resistance, we set out to examine large clinical cohorts of patients who received immunotherapy to determine the survival effect of EGFR activation indicated by *EGFR* driver mutations, high mRNA expression levels, or copy number amplifications (CNAs). Intriguingly, we found that high *EGFR* expression levels were indicative of unfavorable outcomes following ICB, but had no survival effect with monochemotherapy, mirroring the effect of *EGFR* mutations in NSCLC (Figure 1, A and B, and Supplemental Figure 1A; supplemental material available online with this article; https://doi.org/10.1172/JCI194377DS1) (22, 23). We next extended our analysis to clinical cohorts involving multiple cancer types, among which high *EGFR* mRNA expression as well as *EGFR* CNAs consistently served as negative indicators for ICB (Figure 1, C and D) (24–26). This result aligns with previous reports that *EGFR* amplification or high expression predicted worse outcomes for patients undergoing ICB treatment (27, 28). On the basis of these findings, we hypothesized that EGFR activation may actively participate in the immunoevasion during ICB treatment.

To test this hypothesis, we first generated genetically engineered murine cell line models, namely B16F10, ASB-XIV and MC38, to mimic the clinical scenarios of *EGFR* mutation or amplification. These cell lines are reported to be sensitive to ICB treatment (29, 30). Lentiviral vectors were utilized to introduce murine *Egfr* cDNAs encoding the WT *Egfr* or L860R mutation (equivalent to human L858R), as well as human *EGFR* cDNAs encoding the L858R mutation, one of the most common oncogenic mutations in patients with LUAD. These cells were s.c. transplanted into C57BL/6 mice for in vivo evaluation of the anti–PD-1 response compared with IgG treatment (Figure 1, E, G, and I). Interestingly, we observed a significant attenuation of antitumor activity in all 3 inflamed tumor models upon transfection of WT or mutated *Egfr/EGFR*, whereas tumors derived from their respective control cell lines retained the sensitivity to anti–PD-1 treatment (Figure 1, F, H, and J). These phenomena highlight the potential of EGFR activation to facilitate immune evasion during ICB treatment.

We next sought to understand how EGFR activation shields tumors from immune attack. Intriguingly, a screen for transcriptomic changes following anti–PD-1 treatment revealed a consistent pattern across ASB-XIV, B16F10, and MC38 models: a distinct gene set showed marked upregulation in empty vector (EV) controls and remained stable in tumors with ectopic expression of WT or mutant *Egfr/EGFR* (Figure 1, K–M, and Supplemental Table 1). To define the biological processes interrupted by EGFR activation, we mapped these genes to 50 hallmark gene sets, and the results showed that the affected genes were most frequently enriched in the “interferon gamma response” pathway across all 3 tumor models (Figure 1N). This finding was confirmed by gene set enrichment analysis (GSEA) on “cellular response to interferon gamma” and “JAK/STAT signaling” pathways (Figure 1, O and P).

To determine the human relevance of our findings, we took advantage of a public dataset on paired samples from patients with melanoma before and after ICB treatment. As expected, patients in the group with low *EGFR* expression at baseline demonstrated a more substantial enrichment of the IFN-γ response pathway following ICB treatment compared with those in the group with high *EGFR* expression (Supplemental Figure 1, B and C) (31). It is noteworthy that the baseline IFN-γ response signature was also found to be significantly lower in patients with NSCLC who had high mRNA expression or mutation of *EGFR*, given that these tumors were constantly interacting with the host immune system (Supplemental Figure 1, D and E) (23, 32). Furthermore, we confirmed that the IFN-γ response signature accurately predicts survival outcomes following ICB treatment but not monochemotherapy (Supplemental Figure 1, F–I). Taken together, these data show that EGFR activation diminished the antitumor efficacy of ICB by impairing the tumor-intrinsic response to IFN-γ.

### EGFR activation decreases IFN-γ–mediated chemokine production to impair leukocyte recruitment.

**Figure 2:**
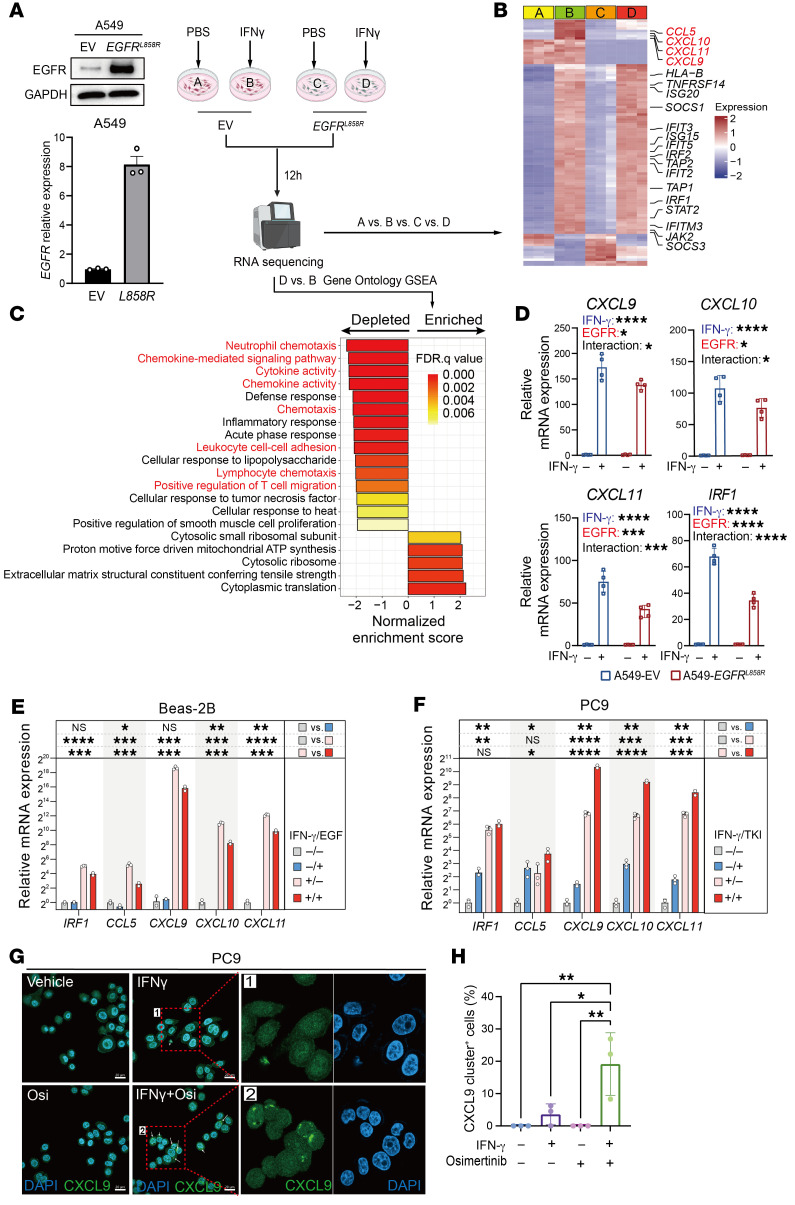
EGFR activation suppresses IFN-γ–mediated chemokine expression and pathways related to chemokine activity. (**A**) Schematic of A549-EV and *EGFR^L858R^* cells treated with PBS or IFN-γ (10 ng/mL) before RNA-seq. (**B**) Top 100 differentially expressed genes with IFN-stimulated genes annotated. (**C**) Top 20 enriched or depleted pathways in A549-*EGFR^L858R^* cells compared with A549-EV cells upon IFN-γ exposure. (**D**) Real-time quantitative PCR (RT-qPCR) analysis of gene expression before and after IFN-γ (10 ng/mL) stimulation for 12 hours in A549-EV and A549-*EGFR^L858R^* cells. (**E**) RT-qPCR analysis of gene expression in WT *EGFR* Beas-2B cells treated with IFN-γ (10 ng/mL), EGF (100 ng/mL), or their combination for 12 hours following 12 hours of serum starvation. (**F**) RT-qPCR analysis of gene expression in *EGFR*-mutant PC9 cells treated with IFN-γ (10 ng/mL), gefitinib (50 nM), or their combination for 12 hours. (**G** and **H**) Immunofluorescence images of CXCL9 (**G**) and positive cell proportions (**H**) after a 24-hour treatment with IFN-γ (10 ng/mL), osimertinib (100 nM), or their combination in PC9 cells. Original magnification, ×400. Data are presented as the mean ± SD. *P < 0.05, **P < 0.01, ***P < 0.001, and ****P < 0.0001, by 2-way ANOVA (**D**), unpaired, 2-tailed *t* test (**E** and **F**), or 1-way ANOVA with Tukey’s test (**H**).

IFN-γ may mediate both antitumor and protumor effects in cancer cells, which are the key responders to IFN-γ during ICB treatment (33). To identify the primary IFN-γ–related biological processes interrupted by EGFR activation, we introduced the *EGFR^L858R^* mutation into the NSCLC cell line A549 and examined its responsiveness to in vitro IFN-γ treatment through bulk RNA-seq (Figure 2A). Notably, a subset of IFN-γ–stimulated genes (ISGs), particularly *CCL5*, *CXCL9*, *CXCL10*, and *CXCL11*, showed markedly higher induction in A549-EV versus A549-*EGFR^L858R^* models (Figure 2B). Consistent with this, GSEA demonstrated that EGFR activation blunted IFN-γ–driven chemokine signaling and chemotaxis pathways (Figure 2C and Supplemental Table 2). To eliminate potential confounding effects from A549’s complex genetic background (e.g. *KRAS^G12S^*, *TP53* mutation), we introduced another classical driver mutation, *EGFR^19del^*, into the Beas-2B immortalized bronchial epithelial cell line, reproducing the observed suppression of IFN-γ–mediated chemotaxis (Supplemental Figure 2, A–C).

**Figure 3:**
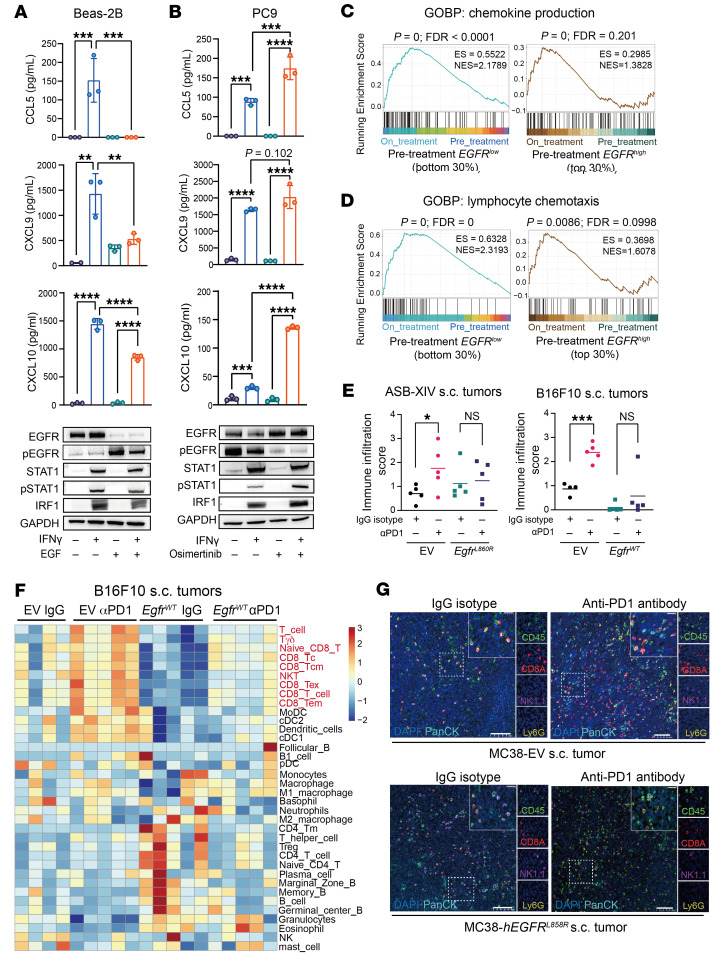
EGFR activation impairs T cell recruitment following ICB by suppressing IFN-γ–mediated chemokine production. (**A** and **B**) ELISA of secreted chemokines in cell culture supernatant and changes in STAT1/IRF1 axis protein levels in Beas-2B cells (12 hours after serum starvation) treated with IFN-γ (10 ng/mL), EGF (50 ng/mL), or their combination for 12 hours (**A**) and in PC9 cells treated with IFN-γ (10 ng/mL), gefitinib (50 nM), or their combination for 12 hours (**B**). (**C** and **D**) GSEA of selected pathways in patients with melanoma before or on treatment with nivolumab, based on pretreatment *EGFR* expression levels (GEO GSE91061). (**E**) Immune infiltration scores for ASB-XIV-EV/*Egfr^L860R^* and B16F10-EV/*Egfr^WT^* tumors treated with anti–PD-1 antibody or IgG. (**F**) Relative abundance of immune subsets in B16F10-EV/*Egfr^WT^* tumors after anti–PD-1 or IgG treatment, estimated from RNA-seq data by the ImmuCellAI mouse algorithm. (**G**) Multiplex immunofluorescence images of immune cells in MC38-EV/*EGFR^L858R^* tumors treated with anti–PD-1 or IgG, related to Figure 1J. Data are presented as the mean ± SD. *P < 0.05, **P < 0.01, ***P < 0.001, and ****P < 0.0001, by 1-way ANOVA with Tukey’s test (**A** and **B**) or unpaired, 2-tailed *t* test (**E**). Original magnification, ×400. ES, enrichment score; NES, normalized enrichment score.

To validate our RNA-seq findings, we modulated EGFR activity across multiple cell lines to evaluate its effect on the expression of the IFN-γ–induced transcriptional factor *IRF1* and these chemokines. As anticipated, in the setting of EGFR activation, either by stable transfection of driver mutations or by EGF ligand stimulation in WT *EGFR* cell lines (A549, H1299, Beas-2B), IFN-γ–mediated upregulation of *CXCL9*, *CXCL10*, *CXCL11*, and *IRF1* was significantly attenuated (Figure 2, D and E, and Supplemental Figure 2, D and E). Conversely, we found that TKI treatment in *EGFR*-mutant cell lines (PC9, HCC827) markedly enhanced these responses (Figure 2F and Supplemental Figure 2F). Consistently, immunofluorescence staining also revealed that inhibition of the EGFR significantly increased the number of IFN-γ–induced CXCL9 protein clusters (Figure 2, G and H). We then measured the secreted protein levels of CCL5, CXCL9, and CXCL10 in the culture medium of Beas-2B and PC9 after ligand activation or TKI suppression of EGFR activity in the presence of IFN-γ, and the results further supported the inhibitory effect of the EGFR on IFN-γ–induced chemokine production (Figure 3, A and B). This effect was found to operate in a dose-dependent manner when cells were exposed to a concentration gradient of EGF or osimertinib (Supplemental Figure 2, G and H). Given these results, we hypothesized that EGFR activation decreases IFN-γ–mediated chemokine production to impair leukocyte recruitment.

*EGFR* driver mutations in LUAD had been associated with decreased TIL abundance in previous reports, although the underlying mechanism remains largely undescribed (13, 34). We analyzed the abundance of TIL subsets in tumor microenvironment (TME) using The Cancer Genome Atlas Program (TCGA) LUAD dataset, which identified T cells as the predominant immune cell population affected by *EGFR* mutations, along with significant downregulation of CD8^+^ T cell effector genes (*GZMB*, *GZMA*, *IFNG*) (Supplemental Figure 2, I and J). These findings well echoed our in vitro demonstration that EGFR suppressed T cell–recruiting chemokines (*CCL5*, *CXCL9*, *CXCL10*, *CXCL11*), with significant coexpression patterns between these chemokine genes and canonical T cell markers (*CD8A* and *GZMB*) providing additional validation (Supplemental Figure 2, K and L). Moreover, the activation of EGFR signaling may also contribute to the exhaustion of T cells, given the increased baseline expression levels of exhaustion-related markers such as *Lag3*, *Havcr2*, and *Pdcd1*, especially in the MC38 tumor model (Supplemental Figure 2, M and N).

In addition to modulating TIL abundance, we found that *EGFR* hyperactivation via mutations or gene amplification could also alter TIL spatial organization, manifesting as a diminished diffuse infiltrative pattern (Supplemental Figure 3, A–C) (35). Conversely, elevated expression of IFN-γ pathway components (*IFNG*, *IRF1*) and chemotaxis-related genes (*CCL5*, *CXCL9*, *CCR5*, *CXCR3*) correlated with an enhanced diffuse infiltrative pattern (Supplemental Figure 3D), suggesting that EGFR-mediated suppression of these pathways may underlie the observed spatial reorganization of TILs.

We next assessed whether *EGFR* overexpression recapitulates the suppressive effect on lymphocyte chemotaxis, as observed with *EGFR* driver mutation or amplification. In patients with NSCLC, high *EGFR* expression correlated with significant downregulation of *CCL5*, *CXCL9*, *CXCL10*, *CXCL11*, and *IRF1* (Supplemental Figure 3E). It is noteworthy that in patients with melanoma, compared with those with higher baseline *EGFR* levels, patients with lower baseline *EGFR* levels exhibited more pronounced chemokine production and lymphocyte chemotaxis following ICB treatment (Figure 3, C and D) (31). This finding was further evidenced by the dynamic changes in these chemokines’ representative genes, including *CCL5*, *CXCL9*, *CXCL10*, *CXCR3*, and *CD8A* (Supplemental Figure 3F). Notably, successful upregulation of these genes following treatment was associated with a higher likelihood of a favorable immunotherapy response, consistent with previous reports (Supplemental Figure 3G) (29). Building upon these observations in the clinical setting, we reanalyzed transcriptomics profiles of subcutaneous MC38, B16F10, and ASB-XIV tumor models. Consistently, we found that tumors harboring *EGFR* mutations or amplification had significant suppression of chemokine signaling pathways and impaired chemotaxis following ICB treatment, ultimately resulting in diminished immune cell infiltration (Figure 3E and Supplemental Figure 3H). Through ImmuCellAI-mouse computational analysis (36), we identified T cells as the most profoundly affected immune cell subset under EGFR-activated conditions, corroborating our earlier findings (Figure 3F and Supplemental Figure 3I). These findings were further validated by flow cytometric analysis and multiplex immunohistochemical staining of MC38 subcutaneous tumors (Figure 3G and Supplemental Figure 3J).

Taken together, these results suggest that cancer cells may serve as a source of specific chemokines, leading to the exposure of their location to immune effectors during ICB treatment. EGFR activation can hinder the recruitment of effector T cells by suppressing IFN-γ–induced chemokine production, thereby facilitating the “camouflage” of cancer cells in the “three Cs” model of immune evasion (37).

### Impairment of ICB efficacy by EGFR activation is partially dependent on EGFR’s inhibition of leukocyte recruitment.

**Figure 4:**
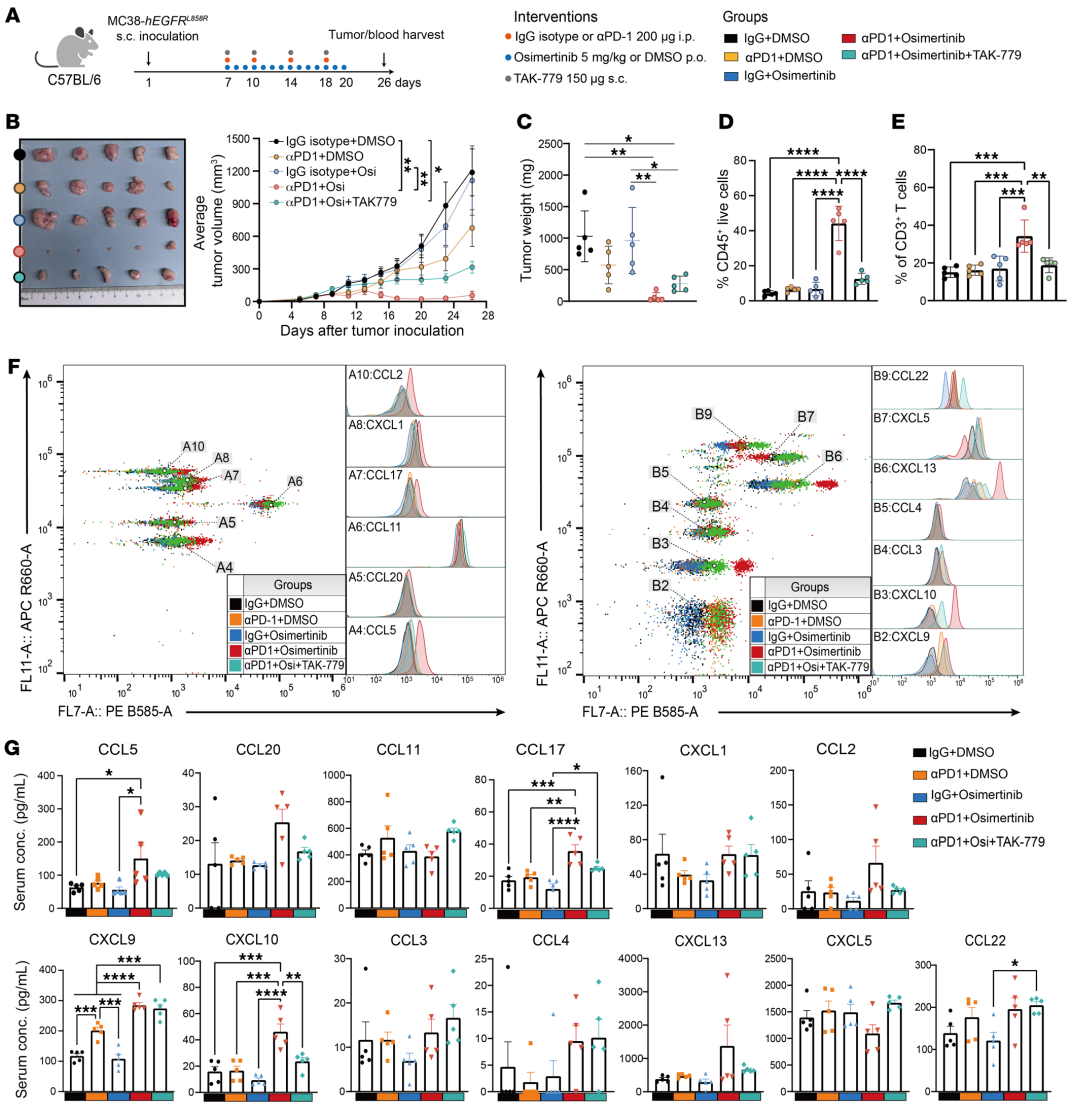
Impairment of ICB efficacy by EGFR activation is partially dependent on its inhibition of leukocyte recruitment. (**A**) Schematic of the dosing strategy and treatment groups in subcutaneous MC38-*EGFR^L858R^* models. (**B**) Image of the harvested tumors and tumor growth curves of MC38-*EGFR^L858R^* models treated with the indicated regimens. (**C**) Tumor weights from the MC38-*EGFR^L858R^* models after completion of the indicated treatments. (**D**) Flow cytometric quantification of CD45^+^ live TILs in MC38-*EGFR^L858R^* tumors. (**E**) Flow cytometric quantification of CD3^+^ T cells among CD45^+^ live TILs in MC38-*EGFR^L858R^* tumors. (**F**) Representative cytometric results of the LEGENDplex assay quantifying 13 proinflammatory chemokines in the serum of mice after completion of the indicated treatments. (**G**) Serum levels of 13 proinflammatory chemokines in mice receiving the indicated treatments. Data are presented as the mean ± SEM (**B**) and as the mean ± SD (**C**–**G**). *P < 0.05, **P < 0.01, ***P < 0.001, and ****P < 0.0001, by 1-way ANOVA with Tukey’s test. Osi, osimertinib.

We next sought to determine whether the impairment of ICB efficacy by EGFR activation is dependent on its inhibition of leukocyte recruitment. First, we found that inhibition of the EGFR using the third-generation EGFR TKI osimertinib resensitized the MC38-*EGFR^L858R^* subcutaneous tumor model to anti–PD-1 antibody and profoundly suppressed tumor growth (Figure 4, A–C, and Figure 1, I and J). This synergistic effect likely stems from enhancement of the leukocyte recruitment capacity under EGFR-inactivated conditions, as evidenced by a marked elevation in CD45^+^ TILs and CD3^+^ T cells within the TME (Figure 4, D and E). To identify the molecular indications of this TME change, we measured the dynamic alteration of multiple chemokines in serum using a cytometric bead array. The results revealed that EGFR TKI significantly boosted the circulating levels of T cell–recruiting chemokines (CCL5, CXCL9, CXCL10, and CCL17) during anti–PD-1 treatment (Figure 4, F and G). Importantly, analysis of clinical trial datasets confirmed that elevated expression of genes encoding these chemokines correlated with improved survival for patients with NSCLC receiving either immunotherapy alone or immunochemotherapy (Supplemental Figure 4, A and B). Interestingly, yet not surprisingly, the expression levels of these genes demonstrated no association with monochemotherapy treatment outcomes (Supplemental Figure 4A) (23, 38, 39).

Meanwhile, we tested whether the blockade of chemokine receptors using TAK-779, a dual blocker of CCR5 and CXCR3, would abolish the synergistic effect of EGFR TKI on ICB treatment. As anticipated, TAK-799 abrogated the recruitment of TILs, particularly of CD3^+^ T cells, into the TME following combination treatment of osimertinib and anti–PD-1 antibody (Figure 4, D and E). Also, the reduced T cell infiltration into the TME may decrease the secretion of IFN-γ and, subsequently, the corresponding IFN-γ–stimulated chemokines (Figure 4G). Intriguingly, we only observed partial rescue of the antitumor effect on CCR5 and CXCR3 inhibition, suggesting the presence of other antitumor mechanisms at play when the cell-derived xenografts were treated with immunotherapy and EGFR-TKI (Figure 4, A–C).

### EGFR activation suppresses IFN-γ–mediated antigen processing and presentation.

**Figure 5:**
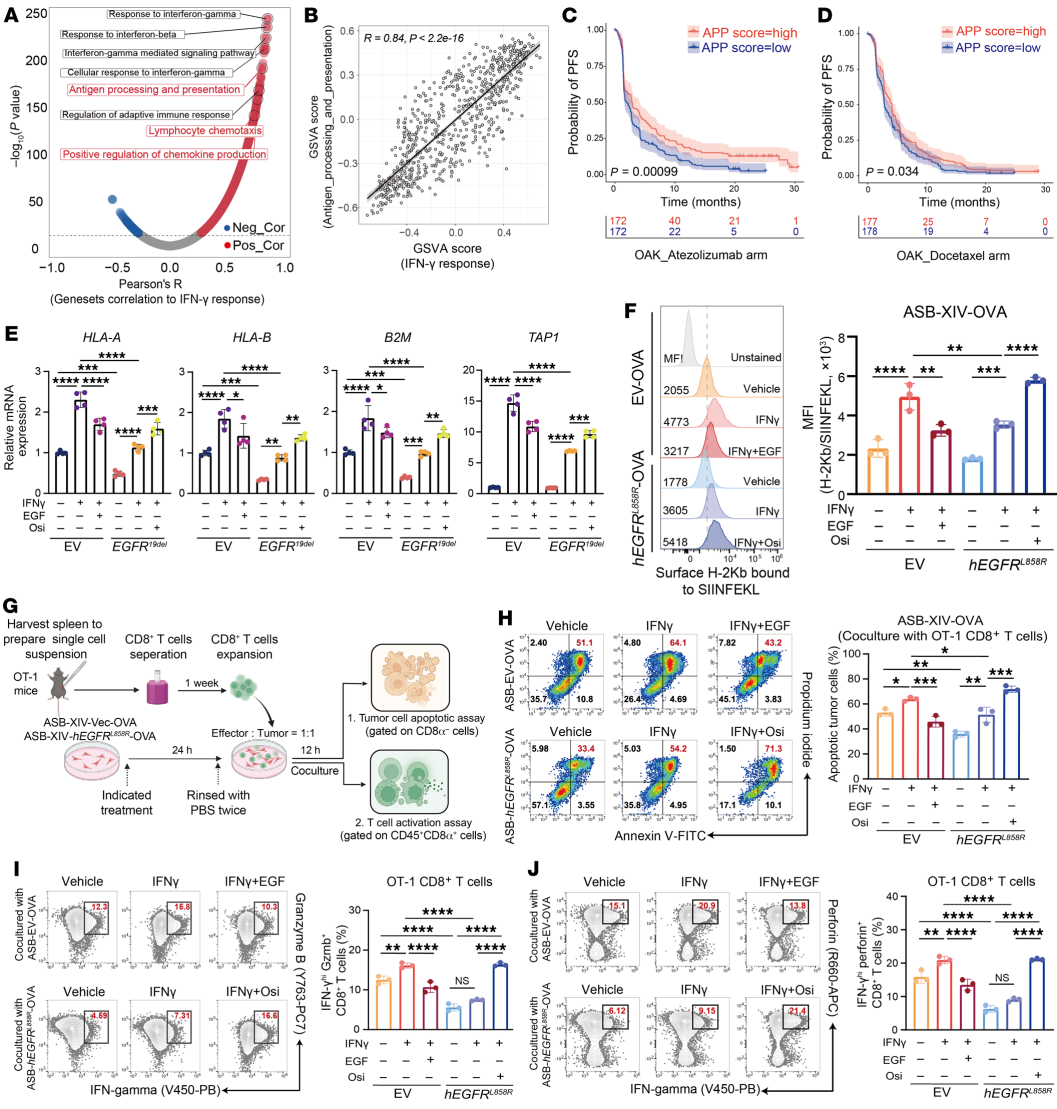
EGFR activation suppresses IFN-γ–mediated APP. (**A**) Pearson’s correlation analysis between the “Hallmark IFN-γ response” signature score and 5,393 GO biological process pathways, assessed by gene set variation analysis (GSVA) on transcriptomic data from patients with NSCLC (OAK trial, *n* = 699). (**B**) Pearson’s correlation plot between GSVA scores for the “Hallmark IFN-γ response” and the “antigen processing and presentation” pathways. (**C** and **D**) PFS of patients with NSCLC who received atezolizumab (**C**) or docetaxel (**D**), categorized by high or low signature scores for APP. (**E**) Change in mRNA expression of APP-related genes in response to IFN-γ (10 ng/mL) in Beas-2B-EV and *EGFR^19del^* cells after a 24-hour treatment with EGF (100 ng/mL) or osimertinib (100 nM) following 12 hours of serum starvation. (**F**) Change in the OVA peptide–presenting capacity in response to mouse IFN-γ (10 ng/mL) in ASB-XIV-OVA models upon genetic or chemical (mouse EGF 100 ng/mL; osimertinib 100 nM for 24 hours) modulation of EGFR activity. (**G**) Illustration of the tumor apoptotic and T cell activation assays in the coculture system of OT-1 T cells and ASB-XIV-OVA cell models. (**H**) ASB-XIV-EV-OVA and ASB-XIV-*EGFR^L858R^*-OVA cells were treated for 24 hours with vehicle, mouse IFN-γ (5 ng/mL) alone, or in combination with mouse EGF (50 ng/mL) or osimertinib (100 nM), followed by 12 hours of coculture with OT-1 T cells. Representative FACS plots (left) and quantification of apoptotic tumor cells (right) are shown. (**I** and **J**) OT-1 T cells were cocultured for 12 hours with ASB-XIV-EV-OVA and ASB-XIV-*EGFR^L858R^*-OVA cells pretreated as in **H**. The proportions of IFN-γ^hi^Gzmb^+^ T cells (**I**) and IFN-γ^hi^perforin^+^ T cells (**J**) among CD45^+^CD8^+^ T cells were analyzed by FACS and compared. Data are presented as the mean ± SD. *P < 0.05, **P < 0.01, ***P < 0.001, and ****P < 0.0001, by log-rank test (**C** and **D**) and 1-way ANOVA with Tukey’s test (**E**–**J**).

As a master regulator of immune responses, IFN-γ coordinates multiple biological processes. We hypothesized that EGFR activation might compromise immunotherapy efficacy by broadly suppressing IFN-γ signaling pathways beyond chemotaxis suppression. We therefore sought to identify therapeutically relevant IFN-γ–regulated pathways vulnerable to EGFR-mediated disruption. Using a data-driven strategy, we quantified the correlations between the IFN-γ response and the complete repertoire of 5,393 gene ontology (GO) terms in a NSCLC cohort (*n* = 699) from the OAK trial (23) (Figure 5A). From this global analysis emerged a top-rated connection to antigen processing and presentation (APP) — a finding particularly compelling, given independent reports of EGFR’s regulation of the antigen presentation machinery (APM) in diverse cancer contexts (Figure 5B) (40–43). Notably, the APP signature has a more pronounced effect on immunotherapeutic efficacy compared with chemotherapy, underscoring the value of targeted modulation of APP during immunotherapy (Figure 5, C and D). While the EGFR is reported to modulate specific APM components independently, its suppression on IFN-γ–dependent antigen presentation remains poorly characterized, representing a potentially targetable mechanism of EGFR-driven ICB resistance.

To delineate the role of the EGFR in IFN-γ–regulated antigen presentation, we activated EGFR signaling in vitro through either a driver mutation or ligand stimulation, which exerted a suppressive effect on IFN-γ–induced expression of multiple APM genes (*HLA-A*, *HLA-B*, *B2M*, *TAP1*). This suppression was reversible with inhibition of the EGFR by osimertinib (Figure 5E). We proceeded to establish OVA-expressing ASB-XIV cell models with concomitantly stable transfection of a lentiviral vector or human *EGFR^L858R^* mutation. This was done to determine whether changes in APM expression could result in corresponding functional alterations in antigen presentation, T cell activation, and antigen-directed cytotoxicity. Indeed, the abundance of H-2K^b^ bound to SIINFEKL on the cell membrane could be significantly enhanced with IFN-γ treatment. However, upon EGFR activation, either through ligand stimulation or introduction of a driver mutation, the extent of this enhancement was notably diminished. Treatment of ASB-XIV-*hEGFR^L858R^* cells with osimertinib fully restored the presented peptides to levels comparable to those in control cells (Figure 5F). Next, we determined the influence of EGFR activation on cytotoxic T cell–mediated (CTL-mediated) killing of tumor cells by using the OT-1–transgenic mouse model (44), with which we isolated T cells engineered to express T cell receptors (TCRs) specifically bound to OVA-derived antigen. Tumor cell models were subjected to IFN-γ stimulation alone or combined treatment with EGF or osimertinib for 24 hours before being cocultured with OT-1 T cells for the purpose of modulating EGFR activity and thereby the level of antigens presented to the cell surface (Figure 5G). Our results indicated that IFN-γ–treated tumor cells were significantly more vulnerable to CTL-mediated killing. As expected, EGFR activation obviously rendered the IFN-γ–treated tumor cells less susceptible to CTL-mediated killing, whereas blockade of the EGFR remarkably augmented the cytotoxic effects of CTLs (Figure 5H). We next evaluated the functional activation of T cells in the coculture systems. Consistently, we found that EGFR activation significantly impaired the capacity of ASB-XIV-OVA cells to activate CTLs, regardless of whether the tumor cells were treated with IFN-γ. Inhibition of the EGFR in IFN-γ–treated tumor cells completely restored the capacity to activate CTLs. This was demonstrated by the changes in proportions of both IFN-γ^hi^ granzyme B^+^ (Gzmb^+^) and IFN-γ^hi^perforin^+^ CTLs (Figure 5, I and J). Consistent with our in vitro findings, *EGFR*-activating mutations in ASB-XIV– and MC38-derived in vivo models substantially undermined the APP capacity, which may account for the models’ weaker T cell activation upon anti–PD-1 blockade (Supplemental Figure 5, A and B, see also Figure 1).

To clinically validate our in vitro and in vivo findings, we analyzed the transcriptomics data from patients with melanoma in paired pre- and post-ICB samples. Elevated baseline *EGFR* expression in patients was associated with a decreased capacity of APP upregulation following treatment (Supplemental Figure 5C), as evidenced by attenuated dynamic changes in key antigen presentation genes, including *HLA-A*, *HLA-B*, *TAP1*, *TAP2*, and *B2M* (Supplemental Figure 5D). Crucially, patients who achieved robust post-treatment upregulation of these genes had superior immunotherapy responses, aligning with our findings for chemokine production (Supplemental Figure 5E). Collectively, these findings highlight the role of EGFR activation in facilitating another dimension of “camouflage” in cancer cells by allowing them to escape T cell recognition through direct impairment of IFN-γ–induced antigen presentation (37).

### The EGFR promotes SHP2 activation to accelerate STAT1 dephosphorylation and abort the IFN-γ response.

**Figure 6:**
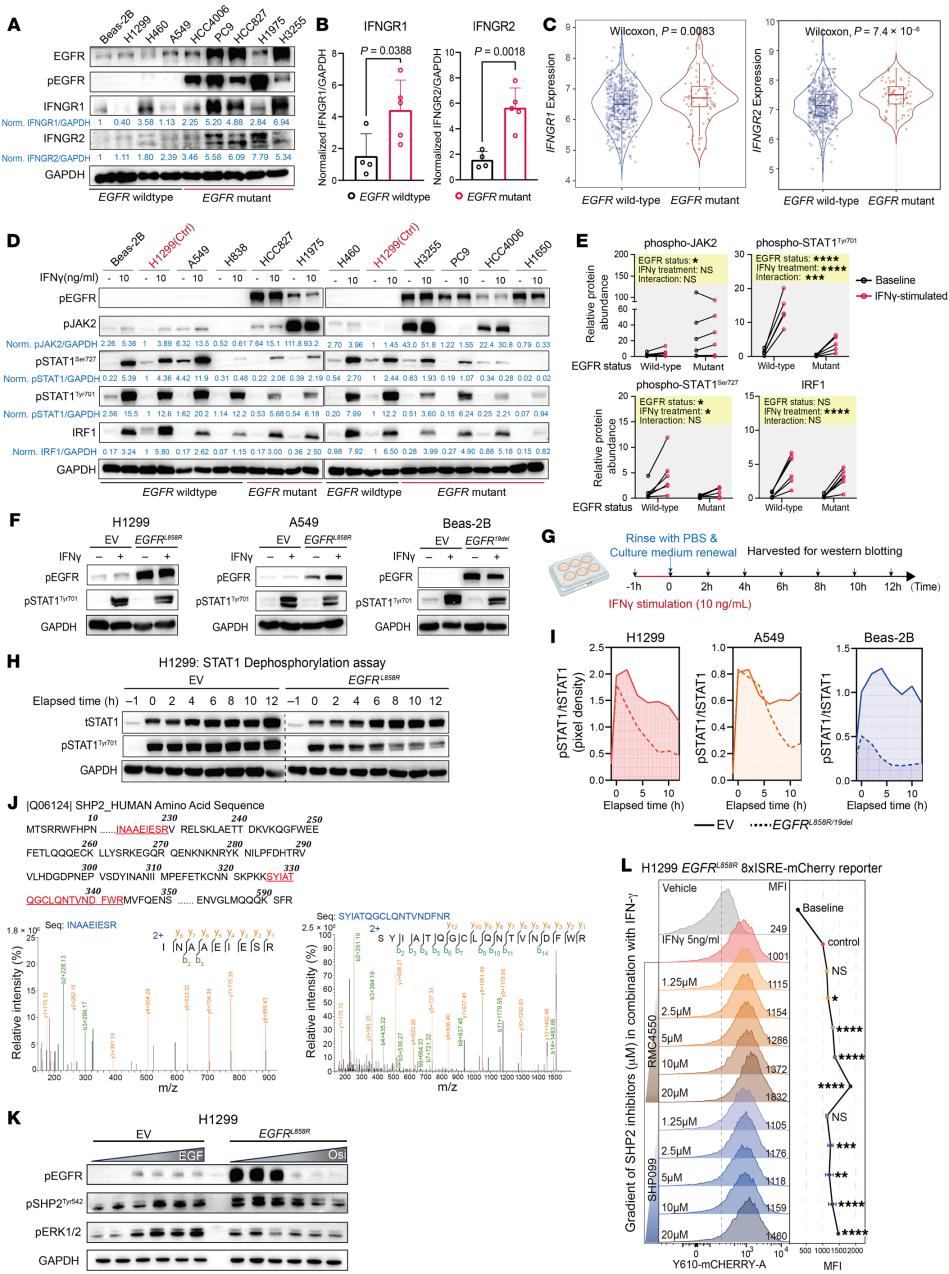
EGFR promotes SHP2 activation to accelerate STAT1 dephosphorylation and abort the IFN-γ response. (**A** and **B**) Western blot analysis of IFNGR1 and IFNGR2 expression in WT *EGFR* and *EGFR*-mutant cell lines (**A**), quantified in ImageJ by pixel intensities and normalized to GAPDH (**B**). (**C**) RNA expression of *IFNGR1* and *IFNGR2* in WT *EGFR* and *EGFR*-mutant LUAD from TCGA dataset. (**D** and **E**) Western blot analysis of IFN-γ signaling markers (pJAK2, pSTAT1, IRF1) in EGFR-WT and *EGFR*-mutant cell lines with or without IFN-γ stimulation (10 ng/mL) (**D**), quantified in ImageJ by pixel intensities and normalized to GAPDH (**E**). (**F**) Western blot analysis of pSTAT1 (Tyr701) at baseline or following stimulation with IFN-γ (10 ng/mL) for 24 hours in H1299, Beas-2B, or A549 cells stably expressing EV, *EGFR^L858R^*, or *EGFR^19del^*. (**G**) Schematic illustration of dephosphorylation assays of pSTAT1 (Tyr701). (**H** and **I**) Western blot analysis of pSTAT1^Tyr701^ at the indicated time points following IFN-γ exposure in H1299-EV/*EGFR^L858R^* cells (**H**), as outlined in **G**. Protein levels were quantified in ImageJ and then plotted as trend curves (**I**). See also Supplemental Figure 6, C and D. (**J**) Identification of the amino acid sequence of SHP2 by mass spectrometric analysis of the IP pulldown. (**K**) Western blot analysis of pSHP2^Tyr542^ and downstream pERK1/2 in H1299-EV/*EGFR^L858R^* cell models treated with a concentration gradient of EGF (0, 1, 10, 50, 100, 500 ng/mL) or osimertinib (0, 10, 50, 100, 1,000, 10,000 nM) for 3 hours. (**L**) FACS analysis of mCherry fluorescence in H1299-*EGFR^L858R^* cells pretreated with a gradient of RMC4550 or SHP099 for 3 hours, followed by combination with IFN-γ (5 ng/mL) for 24 hours. Data are presented as the mean ± SD. *P < 0.05, **P < 0.01, ***P < 0.001, and ****P < 0.0001, by unpaired, 2-tailed *t* test (**B**), Wilcoxon rank-sum test (**C**), 2-way ANOVA (**E**), or 1-way ANOVA with Tukey’s test (**L**).

We next sought to unveil the underlying mechanism mediating the crosstalk between the EGFR and IFN-γ signaling pathways. Our initial hypothesis postulated that the expression levels of IFN-γ receptors may be downregulated in EGFR-activated tumors. Surprisingly, upon assessing protein expression across multiple cell lines, we observed higher expression levels of IFNGR1 and IFNGR2 in *EGFR*-mutant cell lines (Figure 6, A and B), a trend corroborated by the transcriptomics data from The Cancer Genome Atlas–LUAD (TCGA-LUAD) dataset (Figure 6C). This finding implies a potential negative feedback upregulation, and the key crosstalk event likely occurs downstream of the IFN-γ receptor. When we compared IFN-γ responsiveness across cell lines, a paradoxical pattern emerged: despite higher IFN-γ receptor expression, *EGFR*-mutant cells showed markedly blunted signaling activation, as evidenced by the attenuated upregulation of multiple signaling nodes, including phosphorylated STAT1 (pSTAT1) (Tyr701 or Ser727) and IRF1(Figure 6D). This was particularly evident in pSTAT1 (Tyr701) dynamics, which demonstrated the strongest interaction with *EGFR* mutation status (Figure 6E). Subsequently, we confirmed the EGFR dependence of this impaired IFN-γ responsiveness, as introducing *EGFR* driver mutations into 3 WT cell lines (H1299, Beas-2B, and A549) recapitulated the phenotype (Figure 6F). To further support this in vitro finding in a clinically relevant manner, we performed GSEA on paired pre- and post-immunotherapy melanoma samples (Gene Expression Omnibus [GEO] GSE91061) stratified by their baseline *EGFR* expression levels. We found that in the *EGFR*^lo^ group, immunotherapy significantly enriched gene sets related to tyrosine phosphorylation of the STAT protein and the signaling pathway via STAT. In contrast, no such enrichment was observed in the *EGFR*^hi^ group (Supplemental Figure 6, A and B, and Supplemental Tables 3 and 4).

Given the paradoxically higher expression of IFNGR1/2 and pJAK2 in *EGFR*-mutant cell lines, we hypothesized that decreased pSTAT1 may stem from enhanced dephosphorylation, which we subsequently tested using a dephosphorylation chasing assay in isogenic *EGFR*-mutant versus EV control cell lines (Figure 6G). Quantitative analysis revealed consistently accelerated pSTAT1/total STAT1(tSTAT1) decay kinetics across all 3 *EGFR*-mutant models originating from H1299, A549, and Beas-2B cells (Figure 6, H and I, and Supplemental Figure 6, C and D). This finding suggested a potential involvement of a phosphatase in the crosstalk between the EGFR and IFN-γ signaling pathways. To identify the phosphatase interacting with STAT1 in the context of EGFR activation, we conducted an immunoprecipitation assay using STAT1 protein as a bait in the cell lysate prepared from the H1299-*EGFR^L858R^* model pretreated with IFN-γ, followed by mass spectrometric analysis. From the 182 interactors identified, SHP2 emerged as the only detectable phosphatase, positioning it as the prime candidate for EGFR-mediated STAT1 inactivation (Figure 6J and Supplemental Table 3). We further validated the protein interaction between SHP2 and STAT1 through in vitro immunoprecipitation in HEK-293T cells (Supplemental Figure 6E). Encoded by protein tyrosine phosphatase nonreceptor type 11 (*PTPN11*), SHP2 functions primarily as an adapter protein downstream of various receptor tyrosine kinases to facilitate the full activation of the RAS/ERK pathway (45). Additionally, as previously established, SHP2 directly interacts with and dephosphorylates STAT1, acting as a well-documented negative regulator of the JAK/STAT signaling pathway (46, 47). It was reported that overexpression of SHP2 in head and neck cancer may inhibit pSTAT1-mediated APM component expression and the secretion of T cell–attracting chemokines, which aligns closely with our observation regarding the effect of EGFR activation on the IFN-γ response (48). However, whether EGFR activation diminishes cellular responsiveness to IFN-γ through the activity of SHP2 has not been elucidated before. Given this knowledge, we postulate that the EGFR promotes SHP2 activation to expedite STAT1 dephosphorylation and thereby aborts the cellular response to IFN-γ.

We subsequently showed that EGFR activation dose dependently phosphorylated SHP2 at Tyr542, thereby relieving SHP2 from autoinhibited conformation and exposing the catalytic site for phosphatase activity (Figure 6K and Supplemental Figure 6F) (45). To functionally link SHP2 to the impaired cellular IFN-γ responsiveness in the context of EGFR activation, we developed an ISRE-mCherry reporter system in isogenic H1299-EV and H1299-*EGFR^L858R^* cells for quantitative monitoring of JAK/STAT1/IRF1 signaling activity (49, 50). We observed that EGFR activation suppressed the transcriptional activity in response to IFN-γ (Supplemental Figure 6G). However, the inhibition of SHP2 activity with 2 different inhibitors — RMC4550 and SHP099 — was able to restore the IFN-mediated transcriptional activity in H1299-*EGFR^L858R^* cells in a dose-dependent manner (Figure 6L). Further analysis of the TCGA-NSCLC cohort revealed that the suppressive effects of high *PTPN11* expression on the IFN-γ response, leukocyte chemotaxis, and APP completely recapitulated those associated with high *EGFR* expression (Supplemental Figure 7, A and B). In particular, patients with high *PTPN11* expression had significantly lower expression of *IRF1*, *CCL5*, *CXCL9*, *CXCL10*, and *CXCL11* (Supplemental Figure 7C). These molecular alterations might contribute to a less diffusely infiltrative TIL structural pattern (Supplemental Figure 7D). Likewise, higher *PTPN11* expression was also associated with significant downregulation of APM components, including *HLA-A*, *HLA-B*, *TAP1*, *TAP2*, and *B2M* (Supplemental Figure 7E). Inhibition of SHP2 activity with RMC4550 and SHP099 potentiated the IFN-γ–mediated antigen presentation (SIINFEKL peptide) in ASB-XIV-*hEGFR^L858R^* cells (Supplemental Figure 7F). Given these findings, it is plausible that *PTPN11* expression could mimic the effects of *EGFR* on ICB treatment outcomes. By analyzing the survival data from the OAK clinical trial, we verified that *PTPN11* expression serves as a reliable predictor for treatment outcomes of ICB but not monochemotherapy (Supplemental Figure 7, G and H). Collectively, our results suggest that SHP2 functions as a proxy for the EGFR in accelerating STAT1 dephosphorylation and halting the IFN-γ response prematurely during ICB treatment.

### Desensitization of the tumor cellular response to IFN-γ upon EGFR activation is dependent on the activity of SHP2.

**Figure 7:**
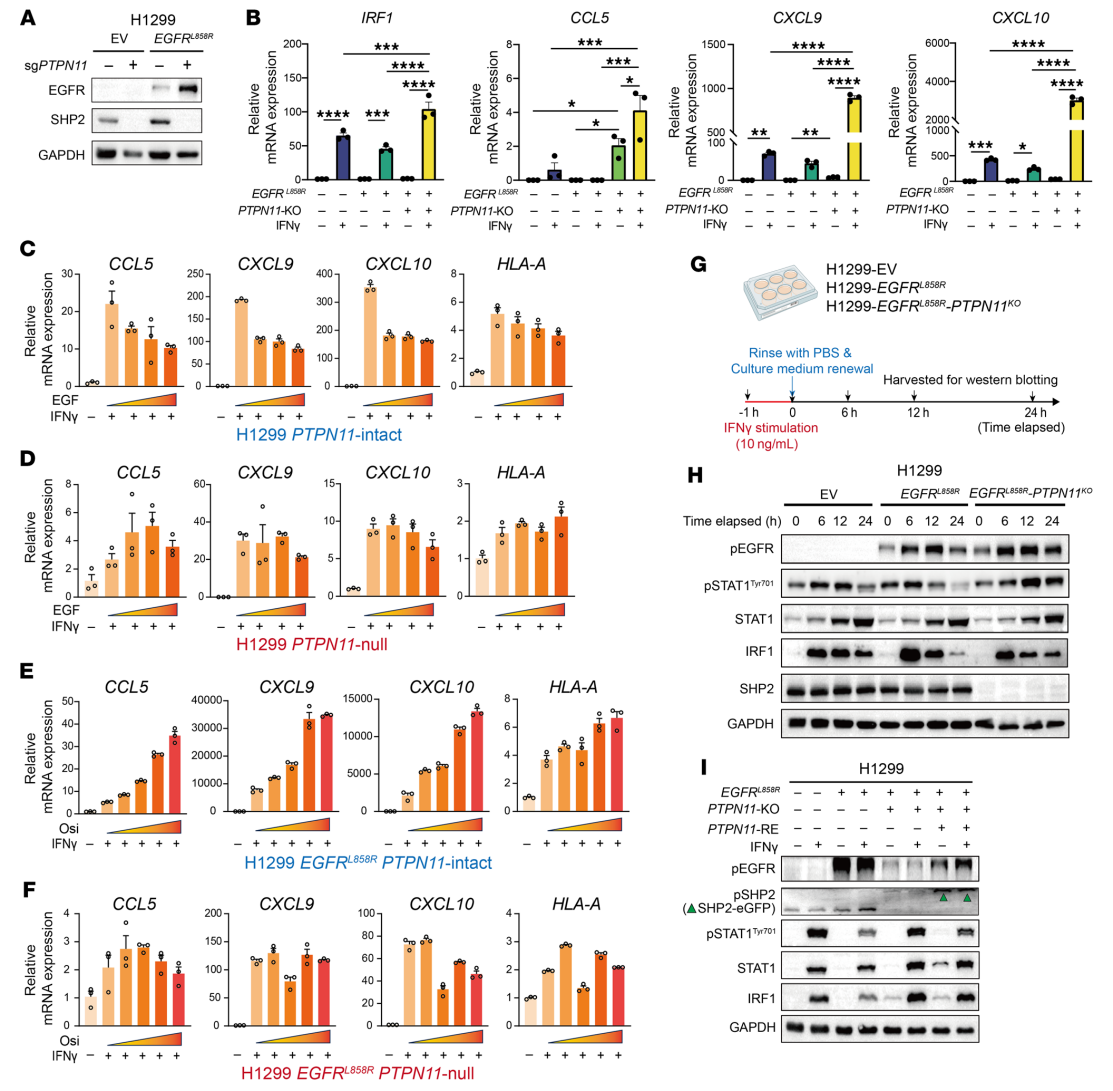
Desensitization of the tumor cell response to IFN-γ upon EGFR activation is dependent on SHP2 activity. (**A**) CRISPR/Cas9 KO of *PTPN11* in H1299-EV or H1299-*EGFR^L858R^* cell models. (**B**) Normalized mRNA expression of *IRF1*, *CCL5*, *CXCL9*, and *CXCL10* in H1299-EV, H1299-*EGFR^L858R^*, and H1299-*EGFR^L858R^-PTPN11^KO^* models at baseline and following stimulation with IFN-γ (5 ng/mL) for 6 hours. (**C** and **D**) Normalized mRNA expression of *CCL5*, *CXCL9*, *CXCL10*, and *HLA-A* in H1299 (**C**) and H1299-PTPN11^KO^ (**D**) cells treated with a concentration gradient of EGF (0, 10, 50, 100 ng/mL) in the presence of IFN-γ (5 ng/mL) for 6 hours following 12 hours of serum starvation. (**E** and **F**) Normalized mRNA expression of *CCL5*, *CXCL9*, *CXCL10*, and *HLA-A* in H1299-*EGFR^L858R^* (**E**) and H1299-*EGFR^L858R^-PTPN11^KO^* (**F**) cells treated with a concentration gradient of osimertinib (0, 10, 50, 100, 500 nM) in the presence of IFN-γ (5 ng/mL) for 6 hours. (**G**) Schematic illustration of dephosphorylation assays of pSTAT1. (**H**) H1299-EV/*EGFR^L858R^*/*EGFR^L858R^-PTPN11^KO^* cell models were harvested for Western blot analysis of IFN-γ signaling nodes (pSTAT1, IRF1) at different time points (0 hours, 6 hours, 12 hours, and 24 hours) subsequent to a 1-hour stimulation with IFN-γ (10 ng/mL). (**I**) Western blot analysis of variations in IFN-γ signaling nodes (pSTAT1, IRF1) in H1299-based cell models treated with the PBS control or IFN-γ (10 ng/mL) for 24 hours. PTPN11-eGFP was reconstituted in the H1299-*EGFR^L858R^-PTPN11^KO^* cell model by transient transfection. Data are presented as the mean ± SD. *P < 0.05, **P < 0.01, ***P < 0.001, and ****P < 0.0001, by 1-way ANOVA with Tukey’s multiple-comparison test (**B**).

Next, we asked whether the diminished responsiveness to IFN-γ upon EGFR activation is dependent on SHP2. We generated *PTPN11*-KO variants in H1299-EV and H1299-*EGFR^L858R^* cells and tested their alteration of the responsiveness to IFN-γ by evaluating the transactivation level of ISGs (Figure 7A). As expected, KO of *PTPN11* fully restored the basal expression of *IRF1*, *CCL5*, *CXCL9*, and *CXCL10* in *EGFR-*mutant cells,and further augmented their upregulation in response to IFN-γ (Figure 7B). In *PTPN11*-intact H1299 cells, we observed a dose-dependent suppressive effect on the transcription of ISGs following EGF stimulation (Figure 7C). However, this dose-dependent pattern was abrogated upon *PTPN11* KO (Figure 7D). Conversely, the dose-dependent activating effect on the transcription of ISGs following osimertinib inhibition was also abolished upon *PTPN11* KO in H1299-*EGFR^L858R^* cells (Figure 7, E and F). We next evaluated the dephosphorylation dynamic of pSTAT1 in these cell models (Figure 7G). Remarkably, we observed a notable delay in pSTAT1 dephosphorylation upon *PTPN11* KO, accompanied by more sustained IRF1 expression (Figure 7H). The reconstitution of *PTPN11* via transient transfection reversed the rescue effect observed with *PTPN11* KO in the *EGFR^L858R^* cell model (Figure 7I). Collectively, these findings suggested that desensitization of the tumor cell response to IFN-γ upon EGFR activation was dependent on the activity of SHP2.

### Inhibition of SHP2 resensitizes the EGFR-activated tumor to ICB treatment through restoration of IFN-γ signaling.

**Figure 8:**
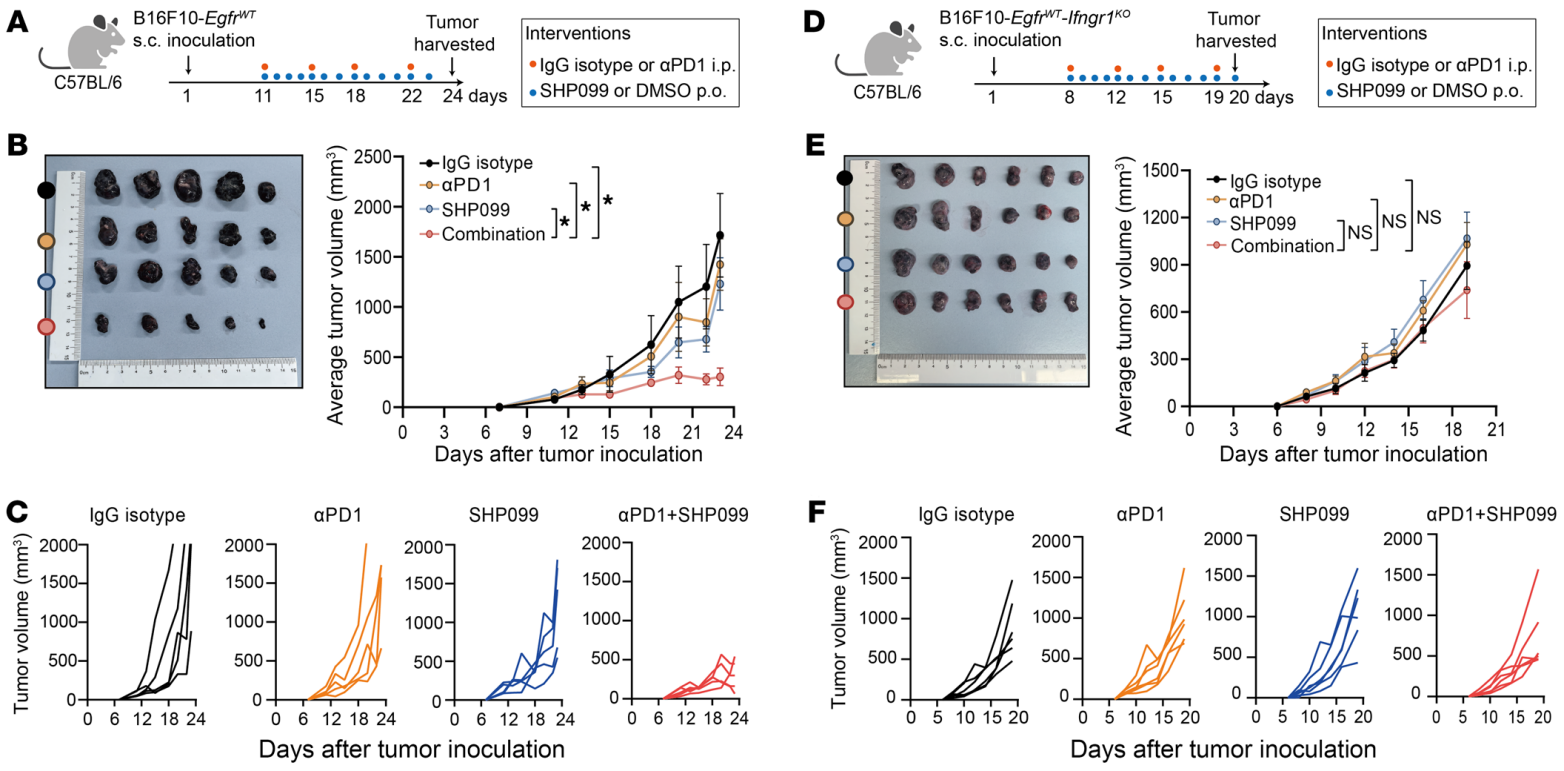
Inhibition of SHP2 resensitizes the EGFR-activated tumor to ICB treatment through restoring the IFN-γ signaling. (**A**) Schematic of drug regimens and dosing strategy (200 μg IgG or anti–PD-1 twice per week for 2 weeks; 75 mg/kg SHP099) in C57BL/6 mice bearing subcutaneous B16F10-*Egfr^WT^* tumors. (**B** and **C**) Overall and individual tumor growth curves of B16F10-*Egfr^WT^* tumors (*n* = 5 mice/group). (**D**) Schematic of the drug regimens and dosing strategy (200 μg IgG or anti–PD-1, twice per week for 2 weeks; 75 mg/kg SHP099) in C57BL/6 mice bearing subcutaneous B16F10-*Egfr^WT^*-*Ifngr1^KO^* tumors. (**E** and **F**) Overall and individual tumor growth curves of B16F10-*Egfr^WT^*-*Ifngr1^KO^* tumors (*n* = 6 mice/group). Data are presented as the mean ± SEM (**B** and **E**). *P < 0.05, by 1-way ANOVA with Tukey’s test.

We previously demonstrated that EGFR activation through ectopic overexpression is sufficient to render the well-established immunogenic tumor models resistant to ICB. This resistance is likely mediated by SHP2, which suppresses the cellular response to IFN-γ. Therefore, we reasoned that inhibition of SHP2 may resensitize the EGFR-activated tumor models to ICB treatment. We tested this hypothesis in the subcutaneous tumor model derived from B16F10-*Egft^WT^* cell lines. As anticipated, we observed that treatment with either anti–PD-1 antibody or SHP099 alone did not lead to a notable shrinkage in tumor size. However, tumor growth was significantly suppressed when we combined anti–PD-1 antibody with the SHP2 inhibitor (Figure 8, A–C). A cytometric bead array revealed significantly increased serum concentrations of multiple proinflammatory chemokines in the combination group (Supplemental Figure 8, A and B). To evaluate whether SHP2 represents a therapeutic vulnerability specifically in EGFR-activated tumors, we further evaluated the antitumor efficacy of SHP099 in combination with ICB in a parental B16F10 cell–derived tumor model. Interestingly, both ICB alone and the combination of ICB with SHP099 significantly suppressed tumor growth compared with the IgG isotype and SHP099 monotherapy. However, the combination did not enhance the antitumor effect beyond that achieved with ICB alone (Supplemental Figure 8, C–E).

We next investigated the origin of this discrepancy in treatment efficacy. RNA-seq of 3 randomly selected subcutaneous tumors from each group revealed that combining SHP099 with anti–PD-1 antibody generally increased the abundance of cytotoxic CD8^+^ T cells in both the parental B16F10 model and the ectopic *Egfr^WT^* overexpression model (Supplemental Figure 8, F and G). Nonetheless, combination with SHP099 remarkably restored the expression of APP-related genes in B16F10-*Egfr^WT^* tumors compared with ICB alone. In contrast, this regimen only marginally improved APP in parental B16F10-derived tumors (Supplemental Figure 9, A and B). To test this finding in an in vitro setting, we compared the effects of SHP2 inhibition on IFN-γ–mediated antigen presentation and CTL killing using ASB-XIV-Vec-OVA and ASB-XIV-*hEGFR^L858R^*-OVA cells. In line with our in vivo findings, we found that the combination of IFN-γ and SHP099 significantly increased apoptosis of the ASB-XIV-*hEGFR^L858R^*-OVA cells but not the ASB-XIV-Vec-OVA cells (Supplemental Figure 9, C–F). These results suggest that SHP2 inhibition may restore ICB sensitivity primarily in tumors exhibiting impaired IFN-γ responsiveness and antigen presentation as a consequence of constitutively active EGFR/SHP2 signaling.

To further validate that SHP2 inhibition restores ICB sensitivity primarily by rescuing tumor-intrinsic responsiveness to IFN-γ, rather than through alleviation of the inhibitory signal of the PD-1/SHP2 axis in immune cells, we knocked out *Ifngr1* in B16F10-*Egfr^WT^* tumor cells to disrupt IFN-γ signaling specifically in the tumor compartment (Supplemental Figure 9, G and H). The established B16F10-*Egfr^WT^-Ifngr1^KO^* subcutaneous tumors in C57BL/6 mice were then treated with IgG isotype, anti–PD-1 antibody, SHP099, or their combination. We found that SHP099 no longer restored the ICB sensitivity of EGFR-activated tumors when the intrinsic response to IFN-γ was abrogated, despite the preserved potential for SHP099 to act on immune cells (Figure 8, D–F). This finding strongly supports the idea that tumor-intrinsic IFN-γ resistance constitutes the dominant mechanism underlying impaired immunity in EGFR-activated tumors.

**Figure 9:**
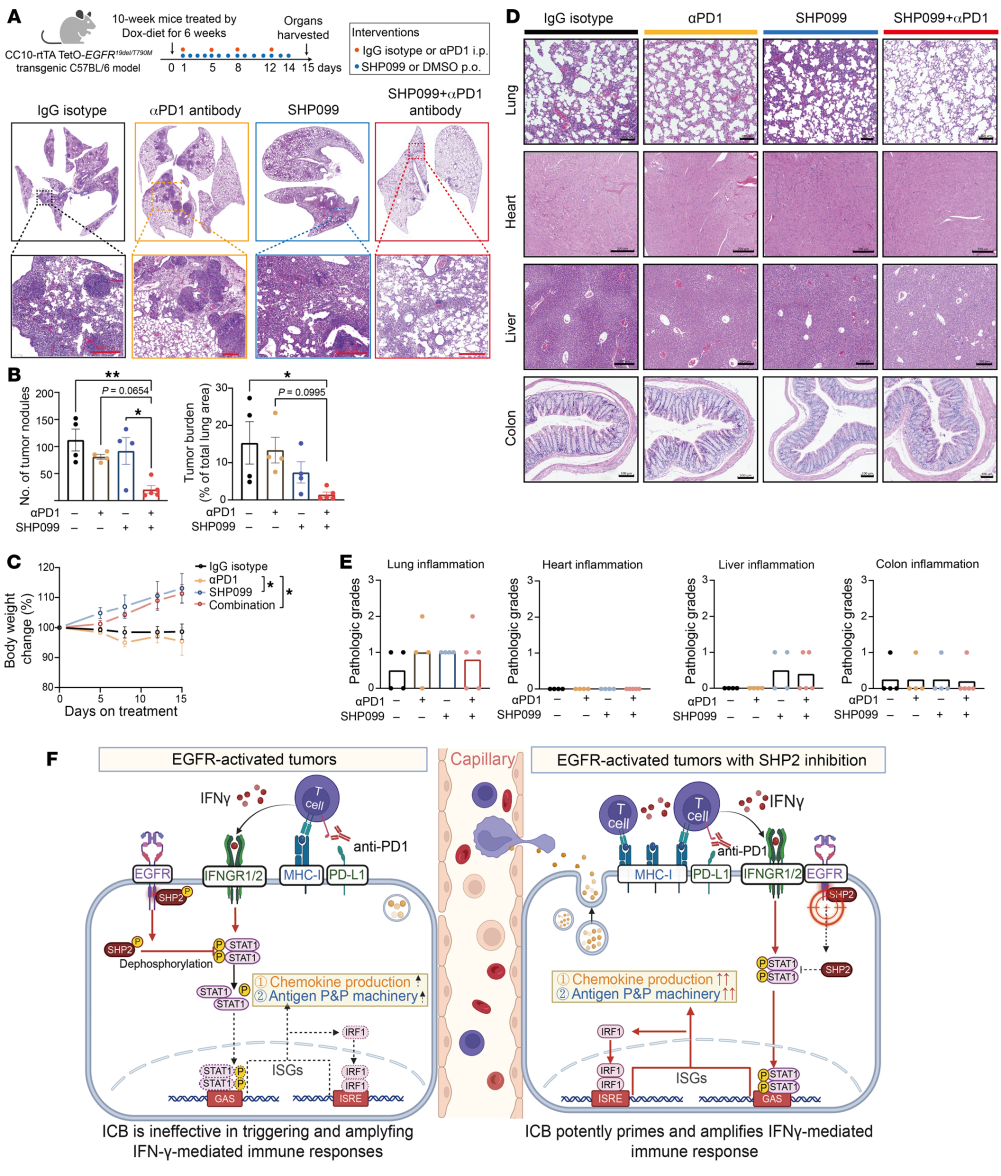
Combining ICB with SHP2 inhibition is effective and potentially safe to overcome primary resistance to immunotherapy in *EGFR*-mutated lung cancer. (**A**) *EGFR^19del/T790M^*-transgenic C57BL/6 mice were treated with doxycycline for 6 weeks to induce lung cancer, and then with IgG or anti–PD-1 antibody (200 μg, 2 times/week i.p.), SHP099 (75 mg/kg daily p.o.), or anti–PD-1 combined with SHP099 for 2 weeks. Representative H&E staining of lungs from each treatment group (*n* = 4–5 mice/group). Scale bars: 500 μm (enlarged insets, bottom). Original magnification, ×100. (**B**) Number of lung tumor nodules (left) and total tumor burden (right) at treatment endpoint. (**C**) Body weight change of transgenic mice during treatment. (**D**) H&E staining of primary organ tissues from transgenic mice after completion of the indicated treatment. (**E**) Pathologic grade of organ-specific inflammation. Scale bars: 200 μm (lung, heart, and liver) and 100 μm (colon, bottom row). (**F**) Schematic illustration of how EGFR activation desensitizes tumors to ICB treatment through SHP2-mediated abrogation of the cellular IFN-γ response, which compromises chemokine production and antigen presentation. Inhibition of SHP2 augments the tumor-intrinsic IFN-γ response, resulting in increased CTL recruitment and activation, which facilitates the reinforcement of a positive feedback loop and resensitizes EGFR-activated tumors to ICB treatment. The schematic in **F** was drawn using BioRender.com. Data are presented as the mean ± SD (**B**) and mean ± SEM (**C**). *P < 0.05 and **P < 0.01, by 1-way ANOVA with Tukey’s test.

We then moved on to evaluate this regimen in orthotopic lung cancer using the CC10-rtTA TetO-*EGFR^19del/T790M^*–transgenic C57BL/6 model. Once again, we verified that *EGFR*-mutant lung cancer was unresponsive to anti–PD-1 blockade. However, the treatment of *EGFR*-mutant lung cancer with SHP099 yielded promising results in resensitizing the immunotherapy (Figure 9A). This was supported by a notable decrease in both tumor nodules and overall tumor burden (Figure 9B). Our results are in agreement with a recent study showing that the combined inhibition of SHP2 and RAS can transform an immune-excluded TME toward an inflamed phenotype and resensitized the immune-resistant tumor model 3LL-ΔΝRAS to anti–PD-1 blockade (51). Interestingly, a substantial enhancement of the tumor-intrinsic IFN response was also documented in the combined therapy, although the underlying mechanism remained undisclosed (51). It is noteworthy that the mice receiving SHP099 or the combination of SHP099 and anti–PD-1 blockade experienced a steady increase in body weight (Figure 9C). This suggests limited toxicity and lends support to the reduction in tumor burden of the lungs. Additionally, the pathologic examination of primary organs prone to immune-related adverse events, including the lung, liver, heart, and colon as part of safety monitoring did not reveal any concerning findings (Figure 9, D and E).

In summary, our study suggests that EGFR activation diminished ICB efficacy through SHP2-mediated suppression of the tumor-intrinsic IFN-γ response. SHP2 inhibition abrogates the inhibitory effects of EGFR activation on IFN-γ–mediated chemokine production and antigen presentation, thereby resensitizing the EGFR-activated cancer to ICB-based treatment (Figure 9F).

## Discussion

The classical “three Es” model of tumor immunoediting (elimination, equilibrium, escape) has recently evolved into the “three Cs” paradigm (camouflage, coercion, cytoprotection) (37). This conceptual evolution marks a fundamental shift from immune-centric to tumor-centric perspectives, recognizing cancer cells as active architects of their own immune evasion. This study, along with our previous report (52), demonstrates that EGFR activation facilitates tumor camouflage by contributing to defective macrophage phagocytosis, impaired recruitment of immune effectors, and abnormalities in APP. Our findings advance the understanding of immune evasion in *EGFR*-mutant NSCLC from passive mechanisms characterized by low TIL abundance, low TMB, or low PD-L1 expression to the recognition of *EGFR* as an active driver in the establishment of immunosuppressive microenvironment (11–13).

In lung cancer, *EGFR*-activating mutations pose a formidable challenge to effective immunotherapy, especially the classical mutations such as *EGFR^19del^* and *EGFR^L858R^* (6, 53). However, for patients with NSCLC harboring atypical or rare *EGFR* variants who are less sensitive to EGFR-TKI, immunotherapy appears to yield better outcomes than in patients carrying classical mutations (7, 54). The underlying mechanisms for this discrepancy remain unclear but may involve structural alterations in the EGFR that might affect its binding affinity to adaptor proteins such as SHP2 (55, 56). Whether tumors with different *EGFR* mutations vary in their responsiveness to IFN-γ remains an open question for future investigation. However, the confounding effects from treatment lines and average TMB across different patient groups should not be ignored (57). In other tumor types in which *EGFR* mutations are infrequent, high expression or amplification of *EGFR* has also been associated with poorer outcomes for patients receiving immunotherapy (27, 28). These observations suggest that EGFR-associated immune evasion probably transcends tumor types; however, the common molecular mechanisms underlying this phenomenon had remained unclear prior to this study. To date, the majority of previous studies have focused primarily on exploring the baseline differences between *EGFR*-mutant and WT lung cancer and inferring resistance mechanisms on the basis of these observations. There has been insufficient study of the EGFR’s effect on dynamic changes during the course of immunotherapy, which could be a more critical determinant of immunotherapy efficacy. Here, our unbiased screening identified impaired cellular responsiveness to IFN-γ in EGFR-activated tumors. This finding can be supported in many previous studies by the alterations in ISGs expressed upon modulation of EGFR activity (43, 58–61). Our subsequent screening revealed that tumor-intrinsic production of T cell–recruiting chemokines and antigen presentation, which are closely related to the IFN-γ response, were notably impaired by EGFR activation during ICB treatment. Nonetheless, our focus on these 2 biological processes does not exclude the involvement of the EGFR in other IFN-γ–regulated programs, such as the regulation of PD-L1 expression. Our findings may help elucidate the apparent discrepancy between preclinical and clinical observations: while EGFR mutation upregulates PD-L1 expression in vitro (62), patients with *EGFR*-mutant NSCLC typically exhibit lower PD-L1 expression levels than do those with WT *EGFR* NSCLC (12, 13, 34, 63). This may be explained by a suppressed tumor response to IFN-γ within the TME owing to constitutive EGFR signaling, which could attenuate IFN-γ–mediated PD-L1 induction. This explanation highlights the complex interplay between constitutive oncogenic regulation and immune regulation of PD-L1.

It is well appreciated that chemokines play multifaceted roles and regulate critical aspects of the immune response, including immune cell trafficking, activation, and function (64). The CCL5-CCR5 and CXCL9/CXCL10/CXCL11-CXCR3 axes are primarily involved in the recruitment of T cells, thereby serving as robust predictors of the antitumor response (65, 66). Given the presence of a chemokine-dependent positive feedback loop, initial differences in the capacity for chemokine production induced by IFN-γ may be amplified over time, leading to substantial discrepancies that ultimately determine the tumor immune contexture (64, 67). This may account for the low TIL abundance in *EGFR*-mutant NSCLC at baseline and its insufficient ability to transition to an inflamed phenotype of the TIME following ICB treatment. Recent studies have revealed that the immunotherapy response probably relies on the replenishment of peripheral T cells rather than the reinvigoration of preexisting exhausted cells (68, 69). Although myeloid cells such as macrophages are also important cellular sources of multiple chemokines, defective production of CTL-recruiting chemokines by tumor cells is sufficient to confer resistance to immunotherapy (70). Therefore, boosting the production of antitumor chemokines from tumor cells represents a potential strategy to overcome the primary resistance to ICB in EGFR-activated tumors (6).

APP by tumor cells is required for the activation of CD8^+^ T cells upon their migration to the tumor bed, which helps to elicit a durable adaptive antitumor immunity (71). Both germline and somatic aberrations in the genes encoding APM components can alter tumor immunogenicity and influence the clinical response to ICB (72, 73). However, dysregulation of APM components is a more commonly encountered defect caused by abnormalities in signaling or epigenetic changes, making them correctable to some extent (74–76). *EGFR*-mutant lung cancer is characterized by a low TMB, which theoretically corresponds to a reduced abundance of neoantigens. Interestingly, a recent study found no significant difference in the spectrum of the HLA class I–presented immunopeptidome between low and high TMB cancers (77). This observation suggests that the impaired capacity to upregulate APM components in response to ICB treatment may be a more important contributor to ICB resistance in *EGFR*-mutant cancers. Notably, IFN-γ–induced APP is also involved in the positive feedback loop of the immune response, whereby the enhanced recognition of target cells further triggers the secretion of IFN-γ by immune effectors (71). This offers a promising chance to sensitize tumors with dysregulated APP to ICB through the appropriate combination therapy (74).

Mechanistically, our study reveals a previously unappreciated link between EGFR and SHP2 activation in accelerating the dephosphorylation of pSTAT1 and thereby aborting the tumor-intrinsic IFN-γ response prematurely. This finding exposes a potential vulnerability that could be targeted to counteract EGFR-driven immune evasion and improve the clinical efficacy of immunotherapy. Indeed, SHP2 plays an immunosuppressive role not only in tumor cells, but also in CTLs and myeloid cells, where its function is primarily linked to the PD-1/PD-L1 axis (78, 79). PD-1/SHP2 signaling was found to suppressed the gene expression profiles responsible for the activation and phenotypic response of T cells, as well as the differentiation of myeloid cell into antitumor states (80, 81). SHP2 inhibition was found to enhance the ICB response partly through alleviation of the immunosuppressive effects of macrophages (79). This implies that the concurrent targeting of SHP2 in immunotherapy could potentially yield multiple advantageous outcomes, especially in *EGFR*-mutant or *EGFR*-overexpressing tumors. However, the extent of tumor-intrinsic or tumor-extrinsic SHP2 inhibition contributing to immunotherapy resensitization may be context dependent and influenced by multiple factors. In our study using a B16F10 model, tumor-intrinsic suppression of IFN-γ signaling by SHP2 was the dominant mechanism underlying immune evasion (i.e., impaired chemokine secretion and APP) in EGFR-driven tumors, whereas the tumor-extrinsic effect of the PD-1/SHP2 axis only played a minor role. Importantly, the differences in the TIME and immunogenicity among various tumor models may influence the magnitude and kinetics of IFN-γ signaling and subsequent biological processes. This inherent difference in the TIME may also limit the robustness of interpreting treatment efficacy and immune modulation upon SHP2 inhibition, as B16F10 is a relatively “cold” and poorly immunogenic model compared with MC38.

It is also important to acknowledge that IFN-γ–driven responses may have dual roles in tumor immunity (33). Therefore, EGFR or SHP2 inhibition could theoretically amplify undesirable effects of IFN-γ — such as PD-L1 upregulation — that favor immune evasion. However, the mechanisms determining the balance between the antitumor and protumor effects of IFN-γ remain incompletely understood. The ultimate biological outcome likely depends on contextual factors such as signal intensity, the duration of activation, and the broader immune microenvironment (33). Further studies are needed to delineate the conditions that favor antitumor immunity over potential immune evasion mechanisms.

In summary, our study revealed that the SHP2-mediated abortion of tumor-intrinsic IFN-γ response was responsible for the compromised immune effector recruitment and antigen presentation following ICB treatment in EGFR-activated tumors. Blockade of SHP2 activity resensitized tumor cells to IFN-γ stimulation, thereby fortifying the positive feedback loop of the immune response and ultimately enhancing the efficacy of ICB treatment. SHP2 combination therapy represents a promising strategy to overcome ICB resistance in *EGFR*-mutant NSCLC.

## Methods

See the Supplemental Methods for a detailed description of all experimental procedures.

### Sex as a biological variable.

Sex was not considered as a biological variable in this study. Clinical data from patients of both sexes were included in this study. Both male and female mice were used in this study.

### Statistics.

Statistical analyses were performed using R version 4.2.1 or GraphPad Prism version 9.0 (GraphPad Software). Survival curves were estimated by the Kaplan-Meier method, compared with the log-rank test, and plotted using the “survival” and “survminer” packages in the R program. A 2-tailed, unpaired Student’s *t* test was applied for experiments involving 2 groups with equal variances in the data. A paired, 2-tailed *t* test was used for comparison of paired pre- and on-treatment samples. The Wilcoxon rank-sum test was used for 2 independent groups when normality or equal variance assumptions were violated. One-way ANOVA with Tukey’s multiple-comparison test was performed for experiments with more than 2 groups. Two-way ANOVA with Tukey’s test was used to evaluate main effects and interaction of 2 factors on a continuous outcome. A *P* value of less than 0.05 was considered statistically significant. Data in the figures are presented as the mean ± SD or mean ± SEM.

### Study approval.

All animal experiments were approved by the IACUC of the Sun Yat-Sen University Cancer Center (L025504202310009) and were conducted in accordance with institutional guidelines. Clinical data from patients in this study are anonymous and publicly available for use in studies.

### Data availability.

Raw RNA-seq data for this study have been deposited in the Biological Project Library at the National Genomics Data Center (https://www.cncb.ac.cn/; BioProject accession codes: PRJCA038433, PRJCA049438, and PRJCA053905). Bulk RNA-seq of NSCLCs from the OAK trial are publicly available in the European Genome-phenome Archive (EGA) (accession code: EGAD00001007703) (23). The bulk RNA-seq and clinical data of pre- and on-treatment melanoma samples are publicly available in the NCBI Gene Expression Omnibus (GEO) database (GEO accession code: GSE91061) (31). The public release of bulk RNA-seq and survival data from the ORIENT11 trial are subjected to institutional regulation restrictions and are only available from the principal investigator, Li Zhang, upon reasonable request (39). The genomic and survival data for NSCLC patients and pan-cancer patients from Memorial Sloan Kettering Cancer Center are publicly available at cBioportal (https://www.cbioportal.org/study/summary?id=tmb_mskcc_2018; https://www.cbioportal.org/study/summary?id=nsclc_mskcc_2018). Values for all data points in graphs are reported in the Supporting Data Values file. This study did not use any custom computer code or algorithms.

## Author contributions

WFF, WTZ, YX Zhang, and LZ conceptualized and designed the study. WTZ, LLP, LYH, J Liao, TL, JNZ, WYY, RXC, TQM, YJH, MJY, and HQZ performed experiments and curated data. WTZ, WFF, LYH, YXZ, RXC, JNZ, ALL, LC, J Li, YMZ, and JHW analyzed and interpreted data. WFF, LZ, LC, and SDH provided resources. WFF and LZ supervised the project. WTZ, WFF, LLP, and JHZ wrote and revised the manuscript. All authors critically discussed the results and approved the manuscript.

## Funding support

- National Natural Science Foundation of China (grants 82173101, 82373262, 82241232, and 82272789, to WFF and LZ).
- Natural Science Foundation of Jiangsu Province, China (BG2025051, to WFF).

## Supplementary Material

Supplemental data

Unedited blot and gel images

Supplemental tables 1-7

Supporting data values

## Version 1 01/15/2026

In-Press Preview

## Version 2 03/02/2026

Electronic publication

## Version 3 07/31/2026

Corrected supporting data values for supplemental figure 2D
