# Supplementary material for: EGFR activation disrupts immunotherapy response via SHP2-mediated suppression of tumor-intrinsic response to IFN-γ
Source: J Clin Invest. 2026 Jan 15;136(5):e194377. doi: 10.1172/JCI194377 (PMC12948428; doi:10.1172/JCI194377)
Supplement: Supplemental data [file jci-136-194377-s010.pdf]

Supplemental Information

EGFR activation disrupts immunotherapy response via SHP2-mediated suppression of tumor-intrinsic response to IFN-γ

Wei-Tao Zhuang, Lan-Lan Pang, Li-Yang Hu, Jun Liao, Jian-Hua Zhan, Ting Li, Ri-Xin Chen, Jia-Ni Zheng, An-Lin Li, Wen-Yan Yu, Tian-Qin Mao, Liang Chen, Yu-Jian Huang, Shao-Dong Hong, Jing Li, Jun-Han Wu, Yi-Ming Zeng, Meng-Juan Yang, Hai-Qing Zeng, Ya-Xiong Zhang, Li Zhang, Wen-Feng Fang

*Lead Correspondence: fangwf@sysucc.org.cn

List of supplemental information:

Supplemental Methods.

Supplemental figure 1. EGFR activation diminishes antitumor efficacy of ICB by impairing cellular response to IFN-γ.

Supplemental figure 2. EGFR activation impairs IFN-γ-mediated chemokine production to compromise CTLs recruitment.

Supplemental figure 3. EGFR activation impairs IFN-γ-mediated chemokine production to compromise CTLs recruitment and spatial organization of TILs.

Supplemental figure 4. Expression of IFN-γ-inducible chemokines are associated with clinical survival outcome of ICB.

Supplemental figure 5. EGFR activation compromises the upregulation of antigen presentation machinery components upon ICB treatment.

Supplemental figure 6. EGFR promotes SHP2 activation to accelerate STAT1 dephosphorylation thereby impairing the upregulation of interferon-stimulated genes.

Supplemental figure 7. Impacts of *PTPN11* expression levels on chemokine production, antigen presentation and immunotherapy outcome recapitulates the impact of *EGFR*.

Supplemental figure 8. SHP2 inhibition restores chemokine production and immune infiltration in EGFR-activated tumor models.

Supplemental figure 9. SHP2 inhibition restores antigen processing and presentation specifically in EGFR-activated tumor models.

Supplemental Methods

Mouse strains

Six- to eight-week old male or female C57BL/6 were obtained from the Animal Center of Sun Yat-sen University Cancer Center (SYSUCC) for subcutaneous implantation of indicated cell line models and subsequent experiments. CC10rtTA/TetO-EGFR$^{19del/T790M}$ C57BL/6 mice were gifts from laboratory of Prof. Liang Chen (College of Life Science and Technology, Jinan University, Guangzhou, China) and were breed in our laboratory in animal center of SYSUCC. OT-1 C57BL/6 mice were purchased from Cavens biogle (Suzhou, China). Mice were maintained under specific pathogen free conditions and randomly assigned to each experimental group.

Cell lines

MC38 (murine colorectal adenocarcinoma, RRID: CVCL_B288), B16F10 (murine melanoma, RRID: CVCL_0159), ASB-XIV (murine lung large cell carcinoma, RRID: CVCL_5686) and human HEK-293T cells (RRID: CVCL_0063) were cultured at 37°C under 5% CO2 in DMEM (Gibco) with 10% fetal bovine serum (FBS, Procell) supplemented with 1% Penicillin/Streptomycin (P/S). A549 (RRID: CVCL_0023), H1299 (RRID: CVCL_0060), H1975 (RRID: CVCL_1511), HCC827 (RRID: CVCL_2063) and PC-9 (RRID: CVCL_B260) human lung cancer cell lines and Beas-2B (RRID: CVCL_0168) human transformed bronchial epithelial cell lines were cultured at 37°C under 5% CO2 in RPMI1640 (Gibco) with 10% FBS supplemented with 1% P/S. All cell lines were authenticated by short tandem repeat analysis before use and routinely tested to be mycoplasma-free. These cell lines were sourced from the American Type Culture Collection except for ASB-XIV, which was purchased from Biocreative (Beijing, China).

Construction of stably transfected cell lines

Lentiviral vectors (pLVX or Lv137) carrying the genes of interest [human *EGFR* (Tsingke Biotech), human *EGFR*^{19del} (Tsingke Biotech), human *EGFR*^{L858R} (Tsingke Biotech), mouse *Egfr* (GeneCopoeia), mouse *Egfr*^{L860R} (GeneCopoeia), gallus SERPINB14-mCherry (GeneCopoeia)] were packaged in HEK-293T cells through co-transfection with packaging plasmids [psPAX2 (RRID:Addgene_12260) and pMD2.G (RRID:Addgene_12259)] or HIV packaging mix provided in the Lenti-Pac™ HIV Expression Packaging Kit (GeneCopoeia, Cat# LT001). The lentivirus-containing culture medium were collected 48 hours post transfection, centrifuged, and filtered through the 0.45 μm polyethersulfone low protein-binding filters. Target cells were seeded onto 6-well plate

one-day before lentivirus harvest to ensure the cell confluent reach 40%–50% when incubated with diluted lentivirus suspension (1:2) and 8 ug/ml polybrene (Beyotime, Cat# C0351). After incubation for 48 hours, stably transfected cells were selected with the puromycin (2-6 ug/ml as appropriate). Cell lines previously transfected with Puromycin-resistant (PuroR) plasmid were sorted through fluorescence activating cell sorting upon secondary transfection with fluorescence-encoding plasmid. All stably transfected cells were then validated through western blotting or quantitative RT-PCR. The established stable cell lines were maintained in fresh complete medium without puromycin for a minimum of 72 hours before experiments to rule out the influence of antibiotics.

In-vivo studies of transplantable tumor models

For in-vivo immune checkpoint blockade (ICB) challenge studies (Result 1), ASB-XIV-EV/$\textit{Egfr}^{L860R}$ (1.5×10^6 cells/100ul), B16F10-EV/$\textit{Egfr}^{WT}$ (1×10^6 cells/100ul) and MC38-EV/$\textit{hEGFR}^{L858R}$ (5×10^5 cells/100ul) were subcutaneous injected into the right flank of 6- to 8-week old C57BL/6. After five to seven days, all mice with subcutaneous tumors reaching approximately 50-75 mm^3 were included for the subsequent experiment and randomly allocated into two treatment groups: one receiving IgG isotype (HRP00256, Hengrui Pharmaceuticals Co.) [200 ug intraperitoneally (i.p.) injection, twice weekly for 2 weeks] and the other receiving anti-mouse PD1 antibody (HRP00262, Hengrui Pharmaceuticals Co.) (200 ug i.p. injection, twice weekly for 2 weeks). Treatment schedule of each tumor model was indicated in the main results. After completion of treatment, the animals were euthanized, and subcutaneous tumors were harvested for subsequent RNA-sequencing, flow cytometry or multiplex immunohistochemistry staining.

For in-vivo EGFR-TKI and chemokine receptor antagonist rescue study (Result 3), MC38- $\textit{hEGFR}^{L858R}$ (5×10^5 cells/100ul) were subcutaneous injected into the right flank of 6- to 8-week old C57BL/6. Seven days after subcutaneous inoculation, all mice with subcutaneous tumors reaching approximately 50-75 mm^3 were included for the subsequent experiment and randomly allocated into five treatment groups: IgG isotype (200 ug i.p. injection, twice weekly for 2 weeks) + DMSO, anti-mouse PD1 antibody (200 ug i.p. injection, twice weekly for 2 weeks) + DMSO, IgG isotype + Osimertinib (TargetMol, Cat#T2490) (5 mg/kg, oral gavage, q.d., for 2 weeks), anti-mouse PD1 antibody + Osimertinib, anti-mouse PD1 antibody + Osimertinib + TAK-779 (TargetMol, Cat#T7499) (150 ug subcutaneous injection, twice weekly for 2 weeks). Treatment schedules for different groups were indicated in the main result. After completion of treatment, the animals were euthanized, and peripheral blood were harvested for profiling the pro-inflammatory chemokines through cytometric bead array (CBA).

For in-vivo mechanistic validation study (Result 7), B16F10-*Egfr*^{WT} (4×10^5 cells/100ul) were subcutaneous injected into the right flank of 6- to 8-week old C57BL/6. All mice with subcutaneous tumors reaching approximately 75-100 mm^3 were randomly allocated into four treatment groups: IgG isotype (200 ug i.p. injection, twice weekly for 2 weeks) + DMSO; anti-mouse PD1 antibody (200 ug i.p. injection, twice weekly for 2 weeks) + DMSO; IgG isotype + SHP099 (TargetMol, Cat#T3544) (75 mg/kg, oral gavage, q.d., for 2 weeks); anti-mouse PD1 antibody + SHP099. Treatment schedules for different groups were indicated in the main result. Body weights of mice were documented for toxicity monitoring during treatment.

All oral-administrated drugs including vehicle control (DMSO) were prepared in 0.5% Sodium carboxymethyl cellulose (CMC-Na) (Selleck, Cat#S6703). All subcutaneous tumors were measured with electronic calipers, and tumor volume (V) was calculated according to the following formula: $V = 0.5 \times \text{Length} \times \text{Width}^2$.

In-vivo studies of transgenic mouse model

Four-week old male or female *EGFR*$^{19del/T790M}$ transgenic mice started treatment by doxycycline diet (Research Diets, Cat# C11300-2000) for 6 weeks to induce the expression mutant-*EGFR* in pulmonary epithelial cells and to allow the development of lung cancer. The doxycycline diet was administered throughout the subsequent experiment. Upon induction of lung cancer, 10-week old mice were randomized into four treatment groups: IgG isotype (200 ug i.p. injection, twice weekly for 2 weeks) + DMSO; anti-mouse PD1 antibody (200 ug i.p. injection, twice weekly for 2 weeks) + DMSO; IgG isotype + SHP099 (75 mg/kg, oral gavage, q.d. for 2 weeks); anti-mouse PD1 antibody + SHP099. Body weights of mice were recorded for toxicity monitoring during treatment. After completion of treatment, the animals were euthanized and the lungs were harvested for histopathologic staining. Only lung tumor nodules with diameter larger than 200 μm were counted and compared between treatment groups. The tumor burden was determined by calculating the ratio of the area occupied by tumor nodules to the total lung area in ZEN v3.1 (Carl Zeiss).

CRISPR-Cas9 gene editing

Double-nicking strategy was applied to generate *PTPN11* knock-out cell models in order to minimize off-target mutagenesis, following the protocol from Zhang's Lab (Supple ref. 1, 2). In brief, paired small guide RNAs (sgRNAs) targeting *PTPN11* were designed (sgRNA-a target sequence: 5′-TCACCCAAATATCACTGGTG-3′; sgRNA-b target sequence: 5′-GCTAGAGTCTATGAAAACGT-3′) and cloned into the pSpCas9(BB)-2A-EGFP

(PX458) plasmid (Tsingke Biotech, RRID: Addgene_48138). Cells were transfected and sorted through FACS. Three PCR primers (Outer Forward: 5′-GCTGTGTTGTTGTGGAAAGTAGTG-3′; Outer Reverse: 5′-CAGGAACAGTGATACCTGCAC-3′; Inner Reverse: 5′-AGGGCCTCAGATAAGGCCTTCA-3′) were designed for screening gene deletion. Sanger sequencing was employed to confirm genome editing, and the knockout efficiency was verified through western blot analysis. *Ifngr1* knock-out cell model was generated using the same method as PTPN11 knock-out cell model except for targeting a single sequence (sgRNA target sequence: 5′-ACTTGAACCCTGTCGTATGC-3′). Two PCR primers were designed for validating genome editing (Forward: 5′-TCATCACCTGCTGCAACTGA-3′; Reverse: 5′-CAGAAGGAAGGATGACGGAA-3′).

Western blot

Whole-cell lysates were prepared with RIPA cell lysis buffer (MIKX, Cat# DB258) in the presence of protease and phosphatase inhibitor cocktails (Cwbio, Cat# CW2200S; CW2383S). Whole-cell lysates were separated by SDS-polyacrylamide gel electrophoresis and then transferred onto polyvinylidene fluoride membranes (Merck Millipore). After blocking with 5% skim milk (diluted in TBST) for 1h at room temperature (RT), membranes were incubated overnight at 4°C with the primary antibody at the dilution recommended by the manufacturer. Membranes were then washed three times in TBST and incubated with the HRP-conjugated secondary antibody in 5% skim milk for 1h at RT. Membranes were washed again in TBST and the antigen-antibody reaction was visualized in Tanon 5200 imaging analysis system by incubated with chemiluminescence HRP substrate (Merck Millipore). Whenever needed, membranes were stripped (Cwbio) and reprobed with an additional antibody. A full list of primary and secondary antibodies is provided in Supplemental table 5.

Real-time quantitative PCR

Total RNA was extracted from tumor cells using the Rapid RNA Extraction Kit (GOONIE, Cat# 400-100), then reverse transcribed using PrimeScript RT Master Mix (TAKARA, Cat# RR036A-1), and analyzed by real-time quantitative PCR (RT-qPCR) using TB Green Premix Ex Taq (Tli RNaseH Plus) (TAKARA, Cat# RR820A), following the manufacturer’s instructions. The qRT-PCR assay was conducted on the Bio-Rad CFX (96 or 384) system and expression was normalized to that of the *GAPDH* or *ACTB*. The sequences of the primers are listed in Supplemental table 6.

Enzyme-linked immunosorbnent assay (ELISA)

Beas-2B and PC-9 cells (70–80% confluent) were treated under indicated conditions for 24h (as illustrated in Figure 2) and the cell culture supernatant was then collected and used for subsequent ELISA assays measuring the concentration of CCL5, CXCL9 and CXCL10 (Proteintech, Cat# KE00093; KE00165; KE00128). The experiment was performed according to the manufacturer's instructions. Optical Density (OD) values at a detection wavelength of 450 nm were read using the Bio-Tek microplate reader (Agilent). The concentrations of different chemokine proteins were calculated using ELISACalc.

RNA-sequencing and bioinformatic analysis

RNA extraction, library construction and sequencing of the subcutaneous tumor models or A549 and Beas-2B cell lines were performed by LC-Bio Technologies Co., Ltd. (Hangzhou, China) as previously described (Supple ref. 3). In brief, total RNA was extracted using Trizol reagent (Thermofisher) and were quality-controlled by Bioanalyzer 2100 (Agilent). cDNA library was then constructed using SuperScript™ II Reverse Transcriptase (Invitrogen). A 2×150bp paired-end sequencing (PE150) was then performed on an Illumina Novaseq™ 6000 platform.

Bioinformatic analysis was executed in the Rstudio or on the OmicStudio platform accessible at https://www.omicstudio.cn. Differential expression analysis of genes was performed by DESeq2 between two different groups. Genes with a false discovery rate (FDR) below 0.05 and an absolute fold change of ≥2 were considered to be differentially expressed. GSEA was performed using software GSEA (v4.1.0) and MSigDB. Briefly, we input gene expression matrix and rank genes by Signal2Noise normalization method (Supple ref. 4). Enrichment scores and *p* value was calculated in default parameters. |NES|>1, nom. p-val<0.05, FDR q-val<0.15 were considered to be different in two groups. Immune Cell Abundance Identifier (ImmuCellAI) analysis in mice tumor models was performed using the OmicStudio tools at https://www.omicstudio.cn/tool (Supple ref. 5).

Immunofluorescence and colocalization analysis

PC-9 cells were seeded in 12-well dishes containing the round coverslips at 1×10^5 cells/well density. After indicated treatment for 24h, culture medium was discarded and cells were washed once with cold PBS and then fixed with 4% paraformaldehyde for 15 mins. Cells were then washed three times with cold PBS and permeabilized with 0.1% Triton X-100 (in PBS) at RT for 5 mins. After 30 mins blocking in QuickBlock™ Blocking Buffer for Immunol Staining (Beyotime), coverslips were incubated over night at 4℃ with primary antibodies against CXCL9

(Proteintech, RRID:AB_2879086) at a dilution of 1:100 (in PBS). Coverslips were then washed 5 mins at room temperature three times with cold PBS and incubated with Alexa Fluor 488 Goat Anti-rabbit Antibody (Immunoway, Cat#RS3211) at a dilution of 1:500 for 1h at RT. Coverslips were washed again and then sealed with antifade mounting medium with DAPI (Beyotime) at RT. Immunofluorescence signals were detected within 6h under a laser scanning confocal microscope (Carl Zeiss) and images were analyzed by the ZEN software (Carl Zeiss). This experiment was conducted in biological triplicate.

Flow cytometry

For evaluation of the immune infiltration in subcutaneous tumor tissue, tumors were collected at the day of harvest and were mechanically dissociated and incubated in tissue digestion solution [0.5mg/ml DNase-I (Roche, Cat#10104159001), 1mg/ml Collagenase IV (Sigma-Aldrich, Cat# C5138), 1mg/ml Dispase II (Sigma-Aldrich, Cat# D4693) in RPIM1640] at 37℃ for 1h. Single cell suspension was then filtered through 70μm filter and underwent red blood cell lysis. Approximately 10^6 cells/sample were stained for live and dead (Zombie UV™ Fixable Viability Kit, Biolegend, Cat# 423107), blocked for Fc receptor (TruStain FcX™, Biolegend, RRID:AB_1574975) and then stained for cell surface markers at the recommended dilution of each antibody. Cells were then washed twice in PBS and analyzed on Cytoflex cytometer (Beckman Coulter).

For evaluation of the antigen presentation capacity of OVA-expressing ASB-XIV cell models, cells were harvested after indicated treatment through digestion by Trypsin-0.25% EDTA. Digestion was terminated within 90 seconds by culture medium containing 10% FBS and the cells were then washed with cold PBS twice. Cells were subsequently incubated with APC anti-mouse H-2Kb bound to SIINFEKL (Biolegend, RRID:AB_11219595) for 30 mins on ice in the dark, washed twice in PBS, and analyzed on Cytoflex cytometer (Beckman Coulter). This experiment was conducted in biological triplicate. A full list of flowcytometry antibodies is available in Supplemental table 5.

For evaluation of the transcriptional activity of Jak-STAT1-IRF1 signaling pathway, the 8×ISRE-mCherry-expressing H1299 cell models were harvested after indicated treatment through digestion by Trypsin-0.25% EDTA. Digestion was terminated within 2 mins by culture medium containing 10% FBS and the cells were then washed with cold PBS twice. Cells were resuspended in cold PBS and were immediately analyzed for the mCherry fluorescence intensity on Cytoflex cytometer (Beckman Coulter). This experiment was conducted in biological triplicate.

Multiplex cytokines array

The profiling of treatment-induced changes of serum chemokines in C57BL/6 mice was performed using the LEGENDplex™ Mouse Proinflammatory Chemokine Panel (Biolegend, Cat#740451). Briefly, peripheral blood of mice was collected immediately after their euthanasia and allowed to clot for at least 30 mins, followed by centrifugation for 10 mins at 1,000×g. Serum was collected and stored at -80°C before use. Serum samples were diluted 2-fold with Assay Buffer and the assay was performed in a V-bottom plate following the manufacturer's instructions. The processed samples were then read on Cytoflex cytometer (Beckman Coulter) and the generated FCS files were analyzed using Biolegend's LEGENDplex™ data analysis software.

Histology and multiplex immunohistochemistry staining

Mouse subcutaneous tumors or primary organs (lungs, heart, liver, colon) were fixed by 4% paraformaldehyde, dehydrated with ethanol, immersed in xylene, embedded in paraffin and sectioned at 3μm thickness at the largest cross-sectional area. The paraffin-embedded lung tissues were deparaffinized, rehydrated and stained with hematoxylin and eosin (H&E) following the standard protocol. All images were acquired using the ZEISS Axio Scan.Z1 system (Carl Zeiss) and analyzed by ZEN software v3.1 (Carl Zeiss). To evaluate the degree of inflammation, the following criteria described by Jia, et al were used (Supple ref. 6): 0 = no abnormality; 1 = presence of inflammation involving < 25% of the tissue section; 2 = presence of inflammation involving 25−50% of the tissue section; and 3 = presence of inflammation involving > 50% of the tissue section of specific organs.

To evaluate the T cells and NK cells infiltration in the tumor microenvironment, we employed a multiplex immunohistochemistry (IHC) staining technique using the PANO 6-plex IHC Kit (Panovue). Briefly, the paraffin-embedded slides were subjected to deparaffinization and rehydration, followed by microwave treatment for antigen retrieval. After blocking for 20 mins, different primary antibodies [CD45 (Cell Signaling, RRID:AB_2799780), CD8α (Cell Signaling, RRID:AB_2756376), NK1.1 (Cell Signaling, RRID:AB_2892989), pan-Cytokeratin (Abcam, RRID:AB_306047), Ly-6G (Cell Signaling, RRID:AB_2909808)] were applied in a sequential manner at 4°C overnight or at RT for 1-2 hours. This was followed by incubation with horseradish peroxidase-conjugated secondary antibodies and subsequent tyramide signal amplification (TSA) for enhanced detection. After each TSA application, the tumor tissue underwent next-round of microwave treatment. Finally, the nuclei were stained with DAPI after all antigens had been labeled. Whole-slide fluorescent images were acquired

using the PanoScanner 20 (PanoVue) and analyzed in HALO analysis system (Indica Labs).

Co-Immunoprecipitation (Co-IP) assay

Pierce Classic Magnetic IP/Co-IP Kit (ThermoFisher, Cat#88804) was used for immunoprecipitation experiments. Briefly, H1299-*EGFR*^{L858R} (90% confluent) were treated by IFN-γ (10ng/ml, MedChemExpress, Cat#HY-P7025) for 6h and washed once with cold PBS. Approximately 1×10^7 cells were lysed with 1 mL IP lysis buffer (PMSF added before use) (Beyotime) for 10 mins on ice with periodic mixing. The supernatants were then collected after centrifugation (13,000 g×15 mins, 4℃). Antibody against STAT1 (6 µg, Cell Signaling, RRID: AB_2737027) or control rabbit IgG (6 µg, Beyotime, Cat# A7016) were added to the lysates and incubated at 4℃ overnight on a rotator. The lysates were then incubated with 25µl prewashed Protein A/G magnetic beads for 30 mins at RT. Beads was washed twice with IP Lysis/Wash Buffer and once with purified water. The antigen/antibody complex was eluted using 100µl low-pH buffer on a rotator, which was then neutralized and immediately subjected to mass spectrometry analysis.

Liquid chromatography-mass spectrometry (LC-MS)

The LC–MS analysis was performed by LC-Bio Technologies Co., Ltd. (Hangzhou, China). Briefly, the proteins were quantified, followed by reduction and alkylation to unfold the three-dimensional structure. After enzyme digestion, the resulting peptides were extracted and then analyzed in Q-Exactive (ThermoFisher). Results were analyzed in Proteome Discoverer v2.1 (ThermoFisher) to identify the proteins present in the sample. The identified proteins were listed in Supplemental table 7.

OT-1 mice CD8+ T cells isolation

The CD8+ T cells were isolated from the splenocytes of OT-1 mice using EasySep™ Mouse CD8+ T Cell Isolation Kit (Stemcell, Cat#19853) according to the manufacturer's instruction. Briefly, spleens were harvested from 6- to 8-week-old OT-1 mice using aseptic techniques, and then mechanically dissociated in PBS containing 2% FBS to prepare single-cell suspension. CD8+ T cells were then isolated and purified by immunomagnetic negative selection. The CD8+ T cells were then maintained in T cell culture medium [RPIM1640, 10% FBS, 1% P/S, 1mM sodium pyruvate (Macklin, Cat#S817535), 1% HEPES (Sigma-Aldrich, Cat#H0887), 1% MEN non-essential amino acid solution (Biospecies, Cat# Bios-01000), 55 µM β-Mercaptoethanol (Aladdin, Cat#M301574) and 10

ng/ml Recombinant Murine IL-2 (PeproTech, Cat#212-12)] and activated by anti-mouse CD3/CD28 antibodies (BioGems, Cat#05112; 10312) to allow proliferation for 1 week before use.

Co-culture experiments

ASB-XIV-EV-OVA or ASB-XIV-*hEGFR*^{L858R}-OVA cells were seeded at 2×10^5/well in a 12-well plate and allowed to adhere and grow for 12h prior to treatments. These cells were then treated by vehicle control, murine IFN-γ (MedChemExpress, Cat#HY-P7071), murine EGF (Beyotime, Cat#P6114), Osimertinib (TargetMol, Cat#T2490), SHP099 (TargetMol, Cat#T3544) or their combination as illustrated in figure 4G or supplemental figure 7D for 24h to allow the signaling-induced change in OVA-peptide presentation. After completion of treatment, the tumor cells were washed twice with PBS to remove the pretreated agents. $CD8^+$ T cells was centrifuged, resuspended in T cell culture medium in the absence of anti-mouse CD3/CD28 antibodies and cocultured with the ASB-XIV-OVA tumor cell models at a ratio of 1:1 (7.5×10^5 cells/well, as the doubling time of ASB-XIV is about 24h). After a 12-hour or 6-hour co-culture at 37°C under 5% CO2 in T cell culture medium, the co-culture system underwent either a tumor cell apoptotic assay or a T cell activation assay.

Tumor cell apoptotic assay

Tumor cell killing by $CD8^+$ T cells was determined using flow cytometry with the Annexin V FITC/PI Apoptosis detection kit (Elabscience, Cat# E-CK-A211). Upon completion of co-culture, ASB-XIV-EV-OVA or ASB-XIV-*hEGFR*^{L858R}-OVA cells along with the floating cells in cell culture medium were collected, centrifuged and washed twice with PBS. The cell was then suspended in 100μL Annexin V binding buffer and stained with Annexin V-FITC (2.5μL), PI (2.5μL) and APC-Cy7 Rat Anti-Mouse CD8a (1:100, BD Biosciences, RRID: AB_396769) for 15 min in the dark. Finally, add 400 μL Annexin V binding buffer to the staining mixture and analyzed on Cytoflex cytometer (Beckman Coulter). This experiment was conducted in biological triplicate.

T cell activation assay

T cell activation in the coculture system with ASB-XIV-EV-OVA or ASB-XIV-hEGFR^{L858R}-OVA was determined using flow cytometry. BFA/Monensin Mixture (MultiSciences, Cat# CS1002) was added to culture medium 3h prior to the completion of co-culture. Following 12-hour coculture, the floating cells in cell culture medium were collected, centrifuged and washed once with ice-cold PBS. The cells were then stained with anti-mouse antibodies

against CD45 (Brilliant Violet 785™, Biolegend, RRID: AB_2564590), CD8a (APC-Cy7, BD Biosciences, RRID: AB_396769) at their recommended dilution for 20 mins at RT in the dark. Next, cells were washed with PBS, fixed in fixation buffer (Biolegend) for 20 mins at RT and permeabilized using Intracellular Staining Perm Wash Buffer (Biolegend) following the instruction. The cells were then stained with IFN-γ (Brilliant Violet 421™, Biolegend, RRID: AB_2563105), Granzyme B (PE/Cyanine7, Biolegend, RRID: AB_2728381) and Perforin (APC, Biolegend, RRID: AB_2721462) at their recommended dilution for 20 mins at RT in the dark. Cells were washed with Intracellular Staining Perm Wash Buffer (Biolegend, Cat#421002) and resuspended in cell staining buffer (Biolegend, Cat#420201) followed by analysis on Cytoflex cytometer (Beckman Coulter). This experiment was conducted in biological triplicate.

Supplemental figure 1

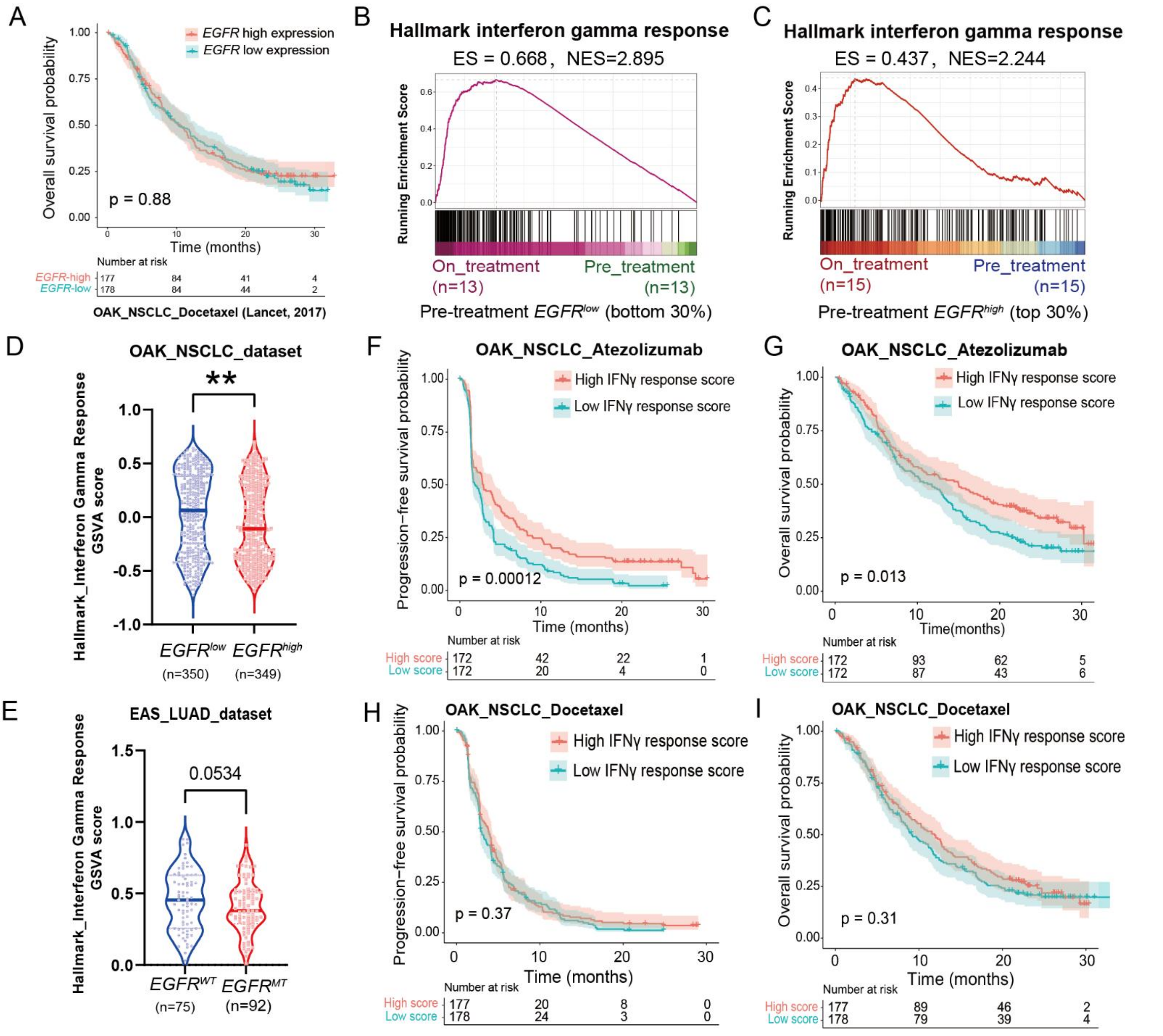

Supplemental figure 1. EGFR activation diminishes antitumor efficacy of ICB by impairing cellular response to IFN-γ. (A) Overall survival curve of NSCLC patients in OAK trial receiving Docetaxel categorized by EGFR mRNA expression. **(B and C)** GSEA of "Hallmark_interferon gamma response" in melanoma patients pre- or on-treatment of nivolumab based on pre-treatment *EGFR* low **(B)** or high **(C)** expression (GSE91061). ES, enrichment score; NES, normalized enrichment score. **(D and E)** GSVA score of the hallmark "interferon gamma response" signature in NSCLC patients of OAK trial **(D)** with high or low levels of *EGFR* mRNA expression and LUAD patients of EAS cohort **(E)** with wildtype or mutant *EGFR*. **(F and G)** Progression-free survival **(F)** and overall survival **(G)** of NSCLC patients receiving atezolizumab, classified by high or low GSVA score of IFN-γ response at baseline. **(I and J)** Progression-free survival **(H)** and overall survival **(I)** of NSCLC patients receiving Docetaxel, classified by high or low GSVA score of IFN-γ response at baseline. Statistical significance determined by log-rank test in (A), (F-I) and unpaired two-tailed t test in (D-E). *** $p<0.001$.

Supplemental figure 2

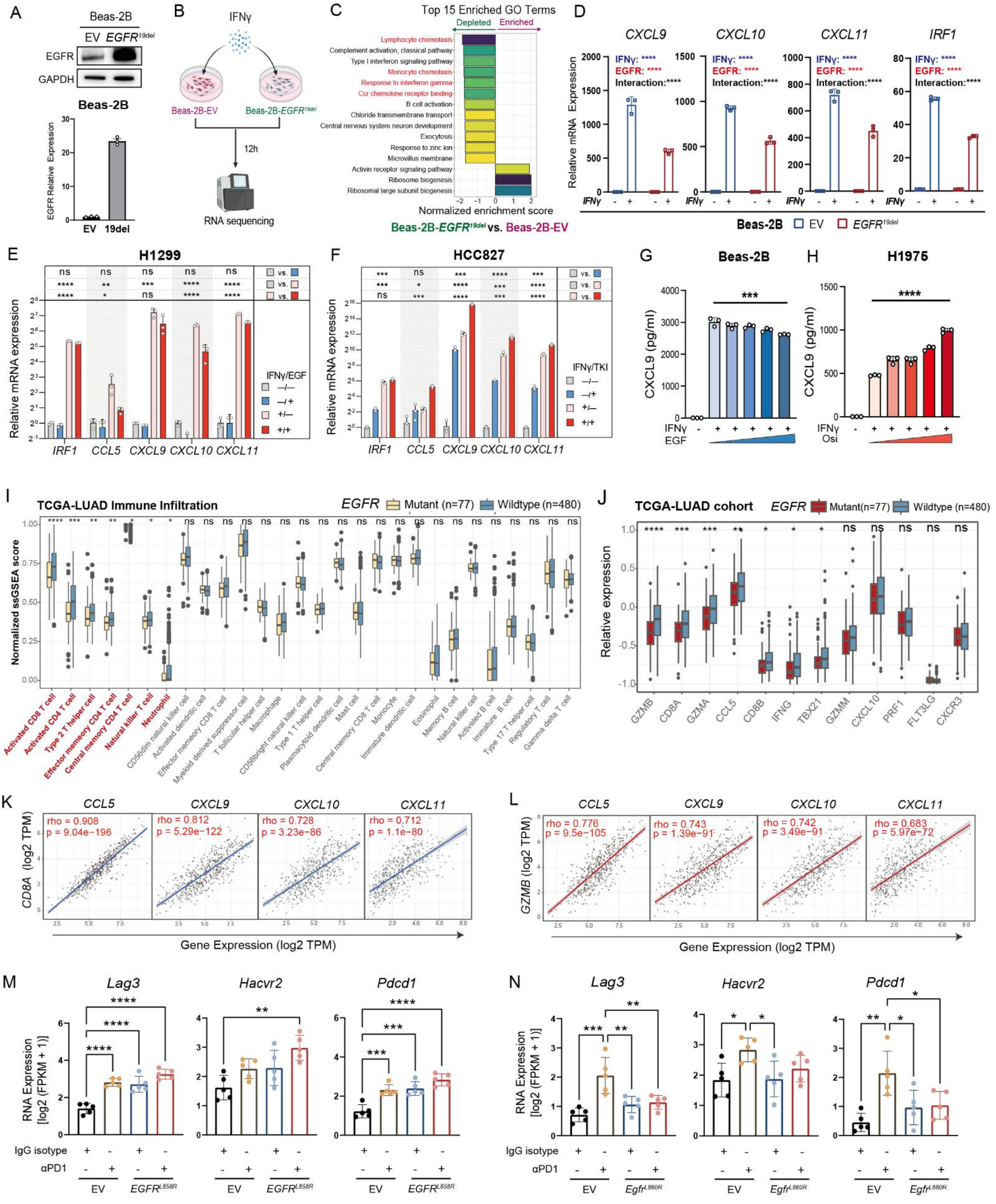

Supplemental figure 2. EGFR activation impairs IFN-γ-mediated chemokine production to compromise CTLs recruitment. **(A)** Western blot and quantitative RT-PCR analysis confirmed the stably transfection of *EGFR*^{19del} mutation in Beas-2B cell line. **(B)** Beas-2B was stably transfected with empty vector (EV) or *EGFR*^{19del} mutation and treated by IFN-γ (10

ng/ml) for 12h prior to bulk RNA-sequencing. **(C)** GSEA result of top 15 enriched or depleted pathways in Beas-2B-*EGFR*^{19del} cells compared to Beas-2B-EV cells upon exposure to IFN-γ (10ng/ml) for 12h. **(D)** Quantitative RT-PCR analysis of *CXCL9/CXCL10/CXCL11/IRF1* expression pre- or post-IFN-γ (10 ng/ml) stimulation for 12h in Beas-2B cell lines stably transfected with EV or *EGFR*^{19del}. **(E)** Quantitative RT-PCR analysis of genes expression in *EGFR*-wildtype H1299 cells after treated by IFN-γ (10 ng/ml), EGF (100 ng/ml) or their combination for 12h following a 12-hour serum starvation. **(F)** Quantitative RT-PCR analysis of genes expression in *EGFR*-mutant HCC827 cells after treated by IFN-γ (10 ng/ml), Osimertinib (50 nM) or their combination for 12h. **(G)** Levels of CXCL9 in cell culture supernatant from Beas-2B treated by a fixed concentration of IFN-γ (20 ng/ml) in combination with a concentration gradient of EGF (0, 25, 50, 100, 200 ng/ml) for 12h. **(H)** Levels of CXCL9 in cell culture supernatant from H1975 treated by a fixed concentration of IFN-γ (20ng/ml) in combination with a concentration gradient of Osimertinib (0, 10, 25, 50, 100 nM) for 12h. **(I)** Tumor immune infiltration of TCGA lung adenocarcinoma (LUAD) patients with or without *EGFR* mutations, derived from single sample GSEA of transcriptomic data. Gene sets obtained from TISIDB (Supple ref. 7). **(J)** Expression levels of T cell markers and effector genes in TCGA-LUAD patients with wildtype or mutant EGFR. **(K and L)** Spearman's correlation analysis of RNA expression between interested chemokines (*CCL5/CXCL9/CXCL10/CXCL11*) and CD8+ T cell marker genes *CD8A* (K) and *GZMB* (L) in TCGA-LUAD dataset. Plots generated in the TIMER2.0 website (Supple ref. 8). (**M and N**) RNA expression levels T cell exhaustion-related markers of MC38-EV/*EGFR*^{L858R} (M) and ASB-XIV-EV/*Egfr*^{L860R} (N) treated by anti-PD1 antibody or IgG control. Data are presented as mean ± SD. Statistical significance determined by two-way ANOVA in (D), unpaired two-tailed t test in (E-F), one-way ANOVA with Tukey's multiple comparison test in (G-H, M, N), Mann-Whitney U test in (I-J). * $p<0.05$, ** $p<0.01$, *** $p<0.001$, **** $p<0.0001$.

Supplemental figure 3

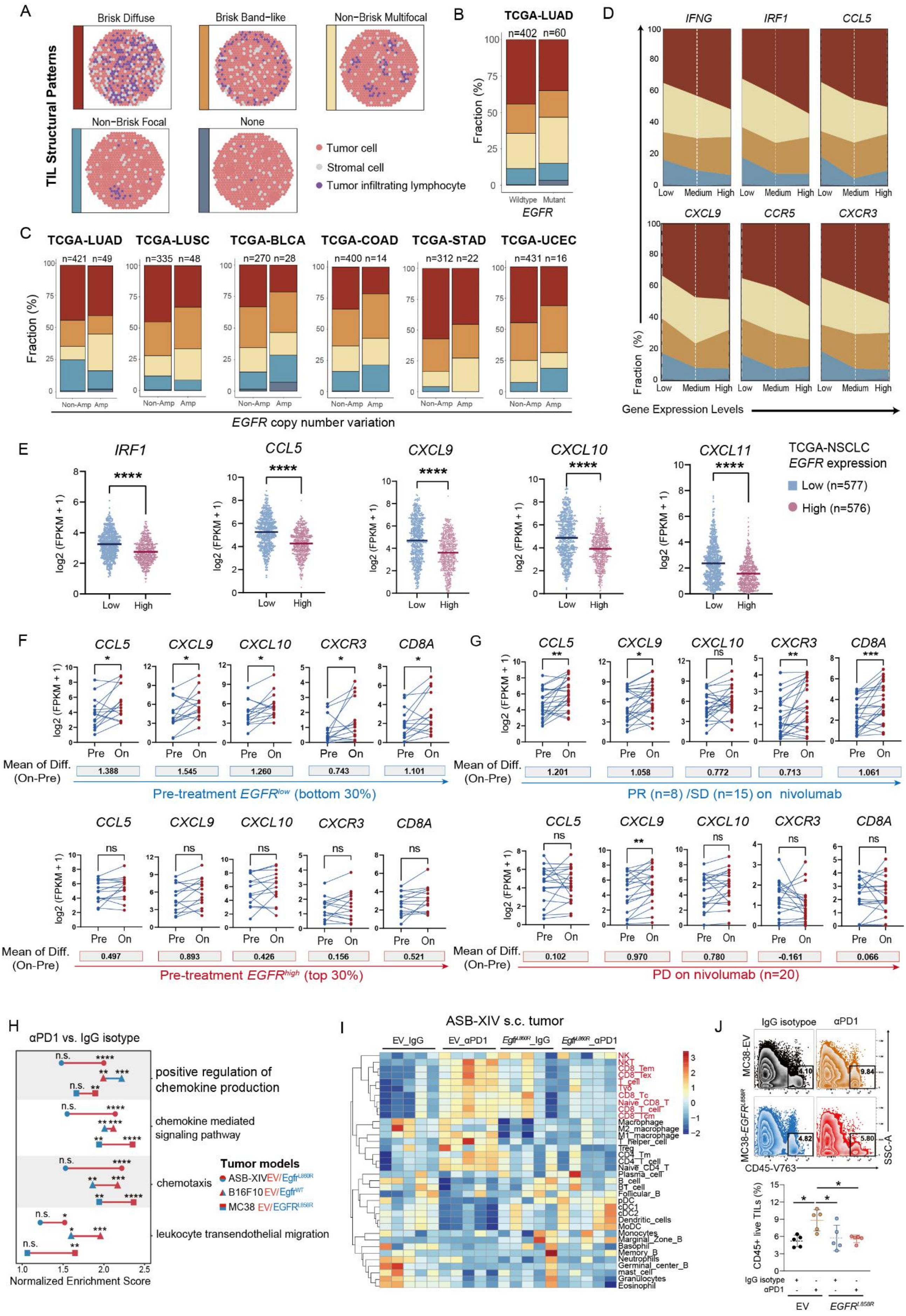

Supplemental figure 3. EGFR activation impairs IFN-γ-mediated chemokine production to compromise CTLs recruitment and spatial organization of TILs. (A) Schematic illustration showing the patterns of spatial organization of TILs defined by a previous study that employed deep learning-based computational stain for TILs on TCGA pathological slides (Supple ref. 9). **(B)** Spatial organization of TILs in TCGA-LUAD samples categorized by *EGFR* mutation status. **(C)** Impact of *EGFR* copy number variations on the spatial organization of TILs in the LUAD, LUSC, BLCA, COAD, STAD, and UCEC samples from TCGA database. **(D)** Spatial organization of TILs in TCGA-LUAD samples categorized by the RNA expression levels of IFN-γ-signature genes (*IFNG, IRF1*), IFN-γ-stimulated chemokines (*CCL5, CXCL9*), and the related chemokine receptors (*CCR5, CXCR3*). **(E)** RNA expression of *IRF1, CCL5, CXCL9, CXCL10,* and *CXCL11* in TCGA-NSCLC samples categorized by high or low *EGFR* expression. Mean expression value is indicated by bar. **(F)** Dynamic change of gene expression prior to or on nivolumab treatment in paired melanoma samples with pre-treatment low (upper panel) or high (lower panel) *EGFR* expression (GSE91061). **(G)** Dynamic change of *CCL5, CXCL9, CXCL10, IRF1, CXCR3, CD8A* RNA expression prior to or on nivolumab treatment in paired melanoma samples categorized by objective treatment response to nivolumab (GSE91061). PD, progression disease; PR, partial response; SD, stable disease. **(H)** GSEA normalized enrichment scores of selected signaling pathways in subcutaneous models of ASB-XIV, B16F10 and MC38 treated by anti-PD1 antibody compared with those treated by IgG control. Related to figure 1E-1J. n.s. not significant, * FDR $q<0.05$, ** FDR $q<0.01$, *** FDR $q<0.001$, **** FDR $q<0.0001$. **(I)** Relative abundance of different immune cell subsets in ASB-XIV-EV/$Egfr^{L860R}$ treated by anti-PD1 antibody or IgG control, estimated from RNA-seq data by ImmuCellAI-mouse algorithm. Related to figure 1E-1F. **(J)** $CD45^{+}$ TILs detected by flowcytometry in MC38-EV/$EGFR^{L858R}$ tumors treated by anti-PD1 antibody or IgG control, related to figure 1J. Statistical significance is determined by unpaired two-tailed t test in (E), by paired two-tailed t test in (F-G), by one-way ANOVA with Tukey's multiple comparison test in (J), * $p<0.05$, ** $p<0.01$, *** $p<0.001$, **** $p<0.0001$.

Supplemental figure 4

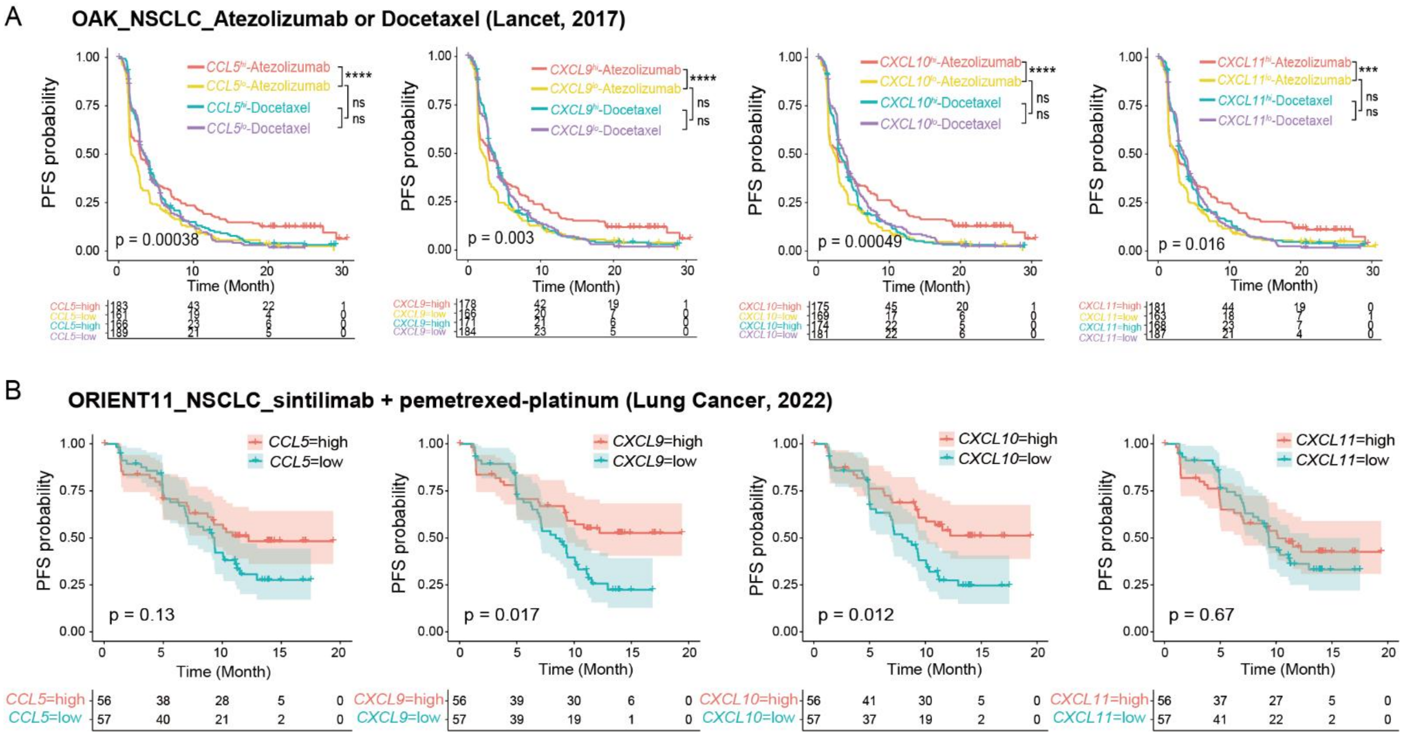

Supplemental figure 4. Expression of IFN-γ-inducible chemokines are associated with clinical survival outcome of ICB. (A) Progression-free survival (PFS) of NSCLC patients categorized by RNA expression levels of *CCL5, CXCL9, CXCL10, CXCL11* in the Atezolizumab arm or Docetaxel arm of OAK trial. **(B)** PFS of NSCLC patients receiving chemoimmunotherapy (Sintilimab, pemetrexed and platinum) in ORIENT11 trial, categorized by RNA expression levels of *CCL5, CXCL9, CXCL10, CXCL11*. Statistical significance is determined by log-rank test. *** $p<0.001$, **** $p<0.0001$.

Supplemental figure 5

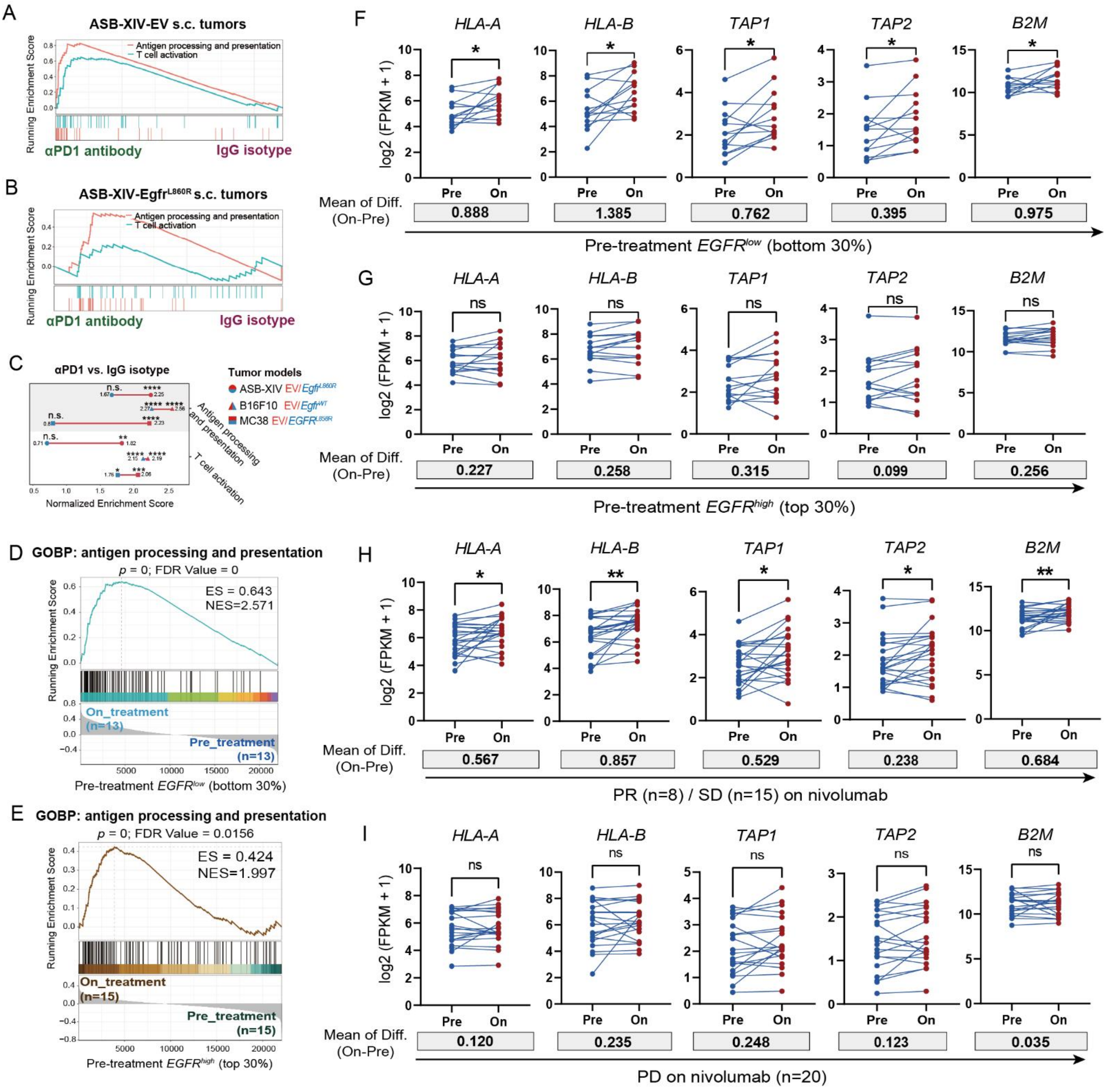

Supplemental figure 5. EGFR activation compromises the upregulation of antigen presentation machinery components upon ICB treatment. (A) GSEA of "Antigen processing and presentation" and "T cell activation" in ASB-XIV-EV (upper panel) and ASB-XIV-*Egfr*^{L860R} (lower panel) subcutaneous tumor models treated by anti-PD1 antibody compared to those treated by IgG isotype. **(B)** Summarized GSEA normalized enrichment scores of "Antigen processing and presentation" and "T cell activation" in subcutaneous models of ASB-XIV, B16F10 and MC38 treated by anti-PD1 antibody compared to those treated by IgG control. Related to figure 1E-1J. n.s. not significant, * FDR q<0.05, ** FDR q<0.01, *** FDR q<0.001, **** FDR q<0.0001. **(C)** GSEA of "Antigen processing and presentation" in samples on

nivolumab treatment compared to paired pretreatment samples from melanoma patients with baseline lower (bottom 30%) or higher (top 30%) EGFR expression (GSE91061). **(D)** Dynamic change of *HLA-A, HLA-B, TAP1, TAP2* and *B2M* RNA expression prior to or on nivolumab treatment in paired melanoma samples with baseline lower (bottom 30%) or higher (top 30%) *EGFR* expression (GSE91061). **(E)** Dynamic change of *HLA-A, HLA-B, TAP1, TAP2* and *B2M* RNA expression prior to or on nivolumab treatment in paired melanoma samples categorized by objective treatment response to nivolumab (GSE91061). PD, progression disease; PR, partial response; SD, stable disease. Statistical significance is determined by paired two-tailed t test in (D-E). * $p<0.05$, ** $p<0.01$, *** $p<0.001$, **** $p<0.0001$.

Supplemental figure 6

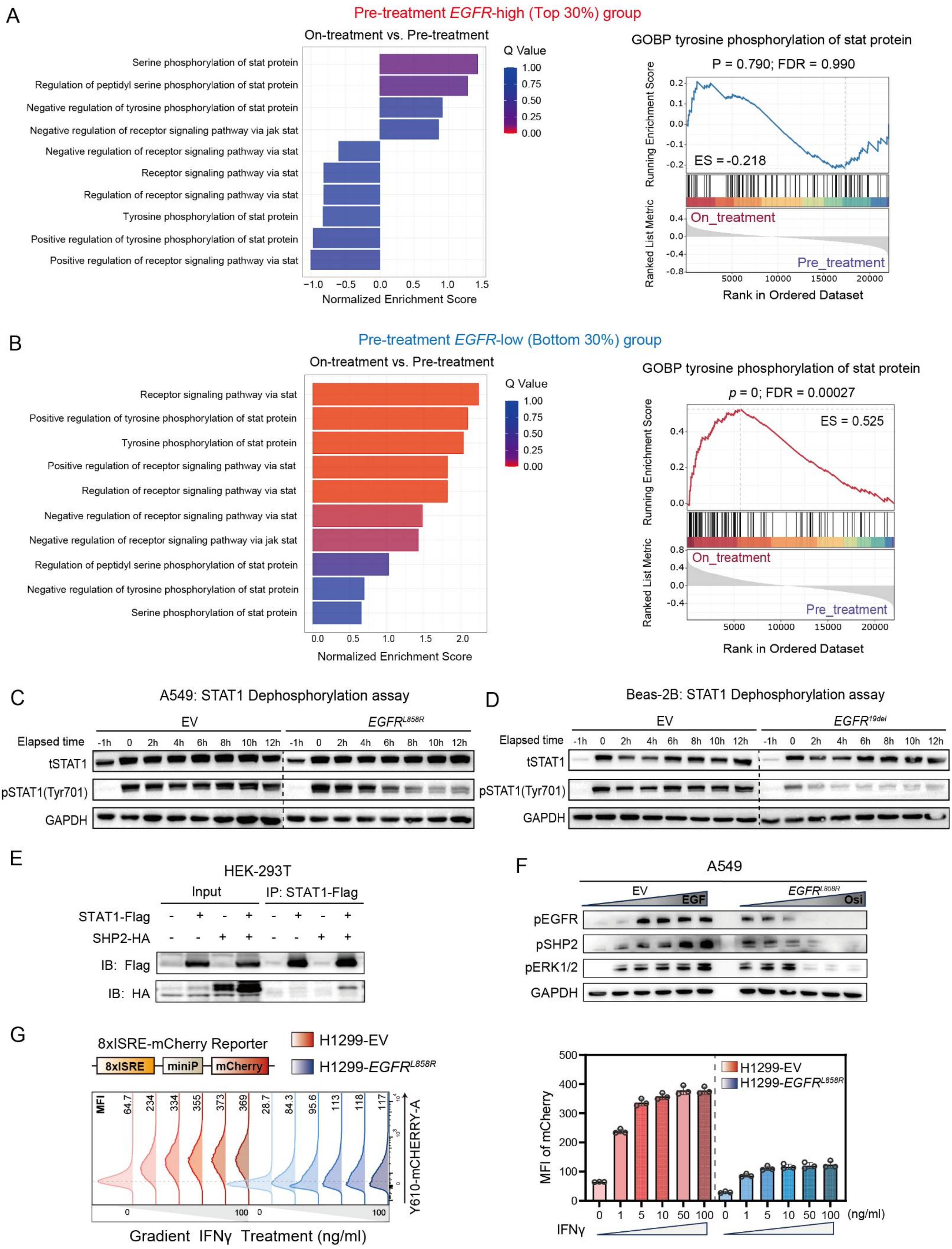

Supplemental figure 6. EGFR promotes SHP2 activation to accelerate STAT1 dephosphorylation thereby impairing the upregulation of interferon-stimulated genes. (A and B) GSEA of 4194 GOBP terms on paired pre- and post-immunotherapy melanoma samples (GSE91061) stratified by their baseline EGFR expression levels and plotting of selected pathways (see also supplemental tables 3 and 4). **(C and D)** Western blot analysis STAT1 dephosphorylation dynamics in A549-EV/$EGFR^{L858R}$ cell models **(C)** or Beas-2B-EV/$EGFR^{19del}$ cell models **(D)** at different timepoints post-IFN-γ exposure, as illustrated in figure 5G. Related to figure 5I. **(E)** Western blot analysis of co-immunoprecipitation between STAT1 and SHP2 in HEK-293T. **(F)** Western blot analysis of pSHP2 and pERK1/2 in A549-EV/$EGFR^{L858R}$ cell models treated by a concentration gradient of EGF (0, 1, 10, 50, 100, 500 ng/ml) or Osimertinib (0, 10, 50, 100,1000,10000 nM) for 3h. **(G)** Construction of an 8×Interferon Stimulated Response Element (ISRE)-mCherry reporter in H1299-EV and H1299-$EGFR^{L858R}$ cell models. Reporter system was validated by FACS analysis of fluorescence intensity of mCherry after 24-hour exposure to a concentration gradient of IFN-γ (0, 1, 5, 10, 50, 100 ng/ml), related to figure 5L. MFI, mean fluorescence intensity.

Supplemental figure 7

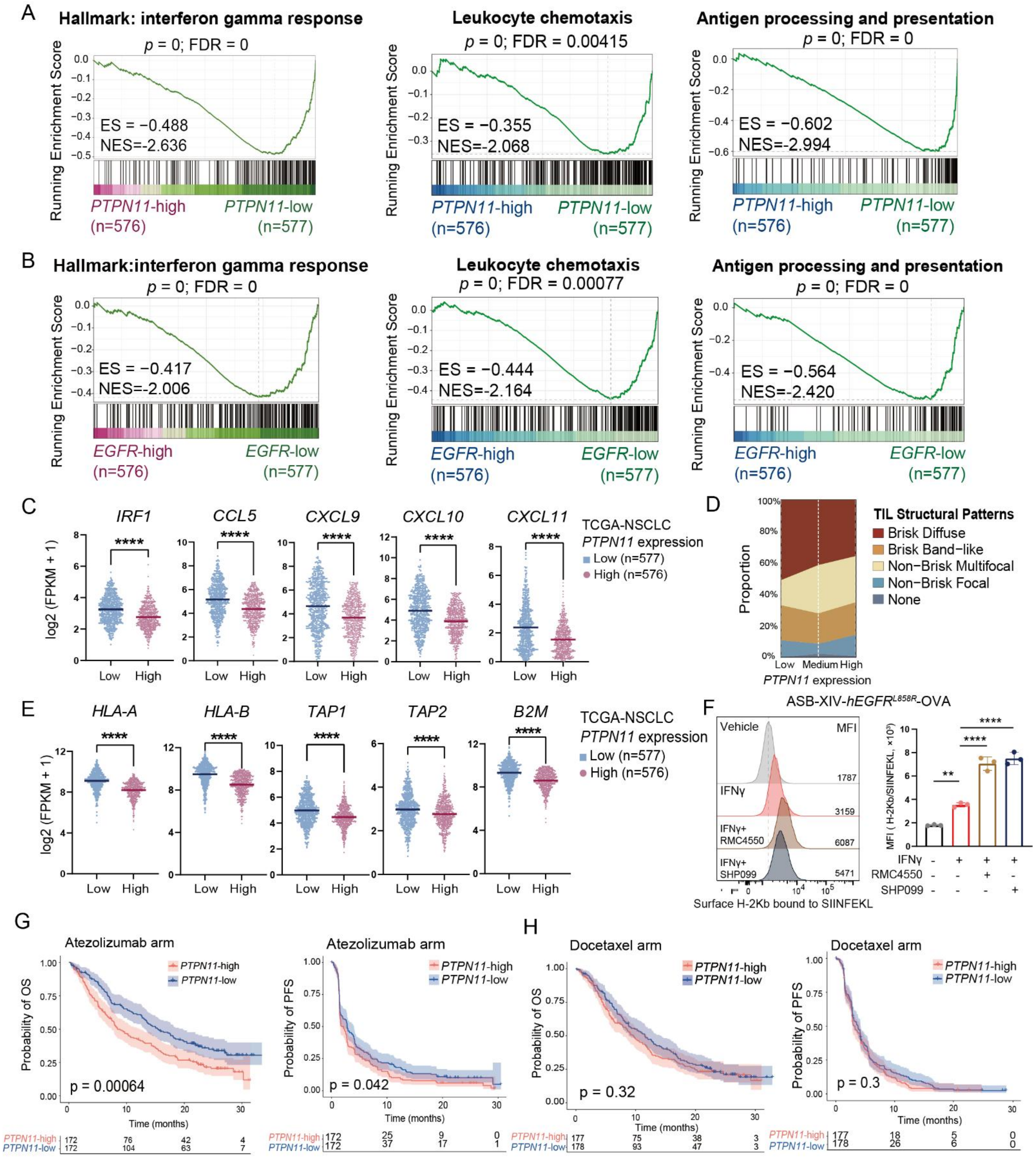

Supplemental figure 7. Impacts of *PTPN11* expression levels on chemokine production, antigen presentation and immunotherapy outcome recapitulates the impact of *EGFR*.

(A and B) GSEA of three gene sets using transcriptome of TCGA-NSCLC cohort (n=1153). Samples were categorized to high or low expression of *PTPN11* (A) and *EGFR* (B) based on their median expression value.

(C) RNA expression of *IRF1, CCL5, CXCL9, CXCL10,* and *CXCL11* in TCGA-NSCLC samples with high or low *PTPN11* expression. Mean expression value is indicated by bar. **(D)** Spatial organization of TILs in TCGA-LUAD samples with low, medium and high expression level of *PTPN11*. Related to Supplemental figure 3A. **(E)** RNA expression of *HLA-A, HLA-B, TAP1, TAP2,* and *B2M* in TCGA-NSCLC samples with high or low *PTPN11* expression. Mean expression value is indicated by bar. **(F)** FACS analysis of OVA-peptide presenting capacity in ASB-XIV-$hEGFR^{L858R}$ cell model treated by PBS, mouse IFN-γ (10 ng/ml), or mouse IFN-γ (10 ng/ml) in combined with RMC4550 (20 μM) or SHP099 (20 μM) for 24h. MFI, mean fluorescence intensity. **(G and H)** OS and PFS curves of NSCLC patients in the Atezolizumab arm (G) or Docetaxel arm (H) from OAK trial. Patients were categorized to high or low expression of *PTPN11* based on the median expression value. Statistical significance is determined by unpaired two-tailed t test in (C, E); one-way ANOVA with Tukey's multiple comparison test in (F) and log-rank test in (G, H). * $p<0.05$, ** $p<0.01$, *** $p<0.001$, **** $p<0.0001$.

Supplemental figure 8

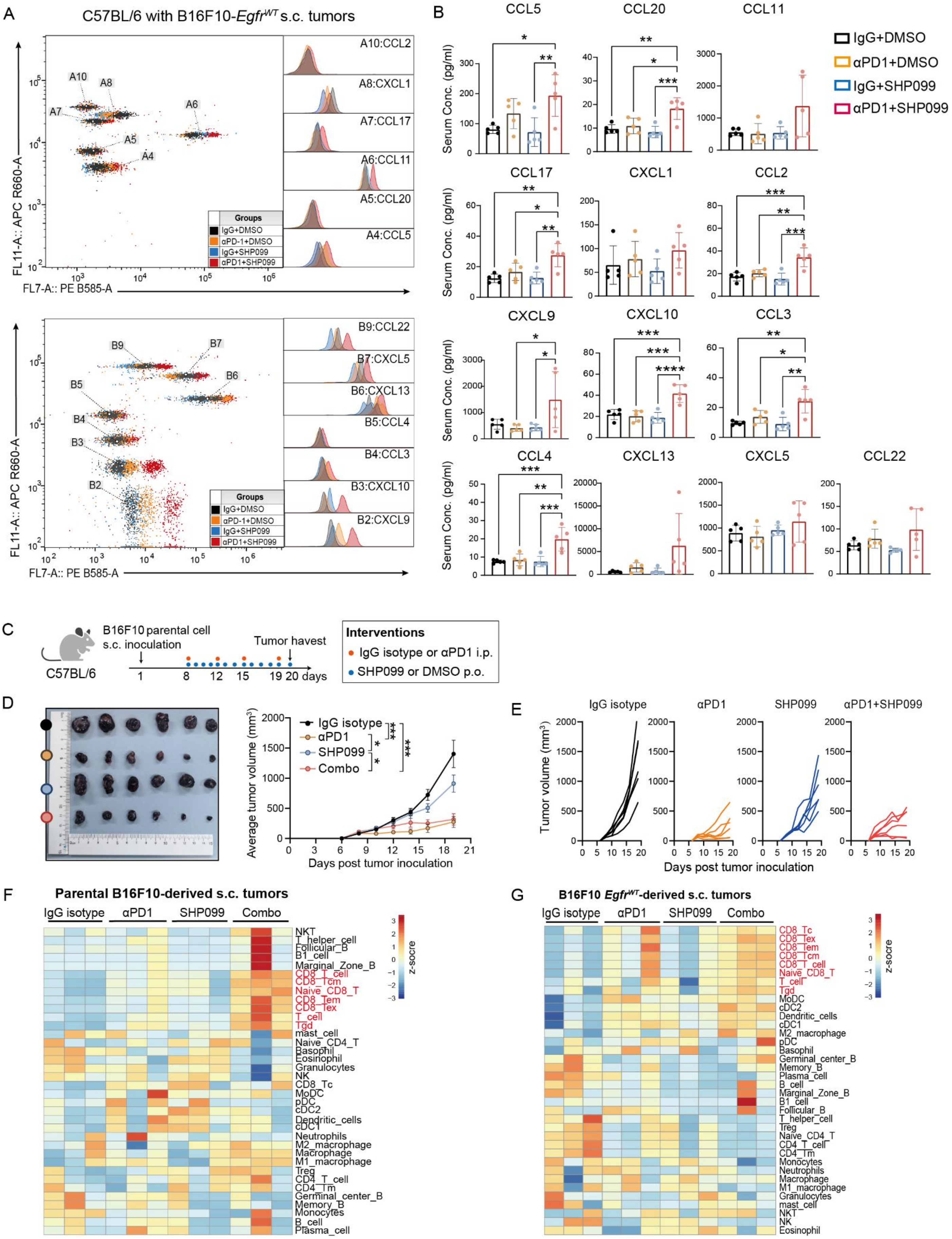

Supplemental figure 8. SHP2 inhibition restores chemokine production and immune infiltration in

EGFR-activated tumor models. (A) Representative cytometry result of LEGENDplexTM assay quantifying thirteen proinflammatory chemokines in the serum of mice bearing B16F10-$Egfr^{WT}$ tumors after completion of indicated treatments (related to Figure 7A-C). **(B)** Statistic analysis of serum concentration of thirteen proinflammatory chemokines in mice receiving indicated treatments. **(C)** Schematic of drug regimens and dosing strategy (IgG or anti-PD1 antibody 200 ug twice per week for two weeks, SHP099 75 mg/kg) in C57BL/6 mice bearing subcutaneous B16F10 tumors. **(D and E)** Overall and individual tumor growth curves of B16F10 tumors (n=6 mice/group). **(F and G)** Relative abundance of different immune subsets in parental B16F10 (F, related to supplemental figure 7C-E) or B16F10-$Egfr^{WT}$ (G, related to supplemental figure 7A-C) estimated from RNA-seq data by ImmuCellAI-mouse algorithm. Data are presented as mean±SD in (B) and mean±SEM in (D). Statistical significance determined by one-way ANOVA with Tukey's multiple comparison test. * p<0.05, ** p<0.01, *** p<0.001, **** p<0.0001.

Supplemental figure 9

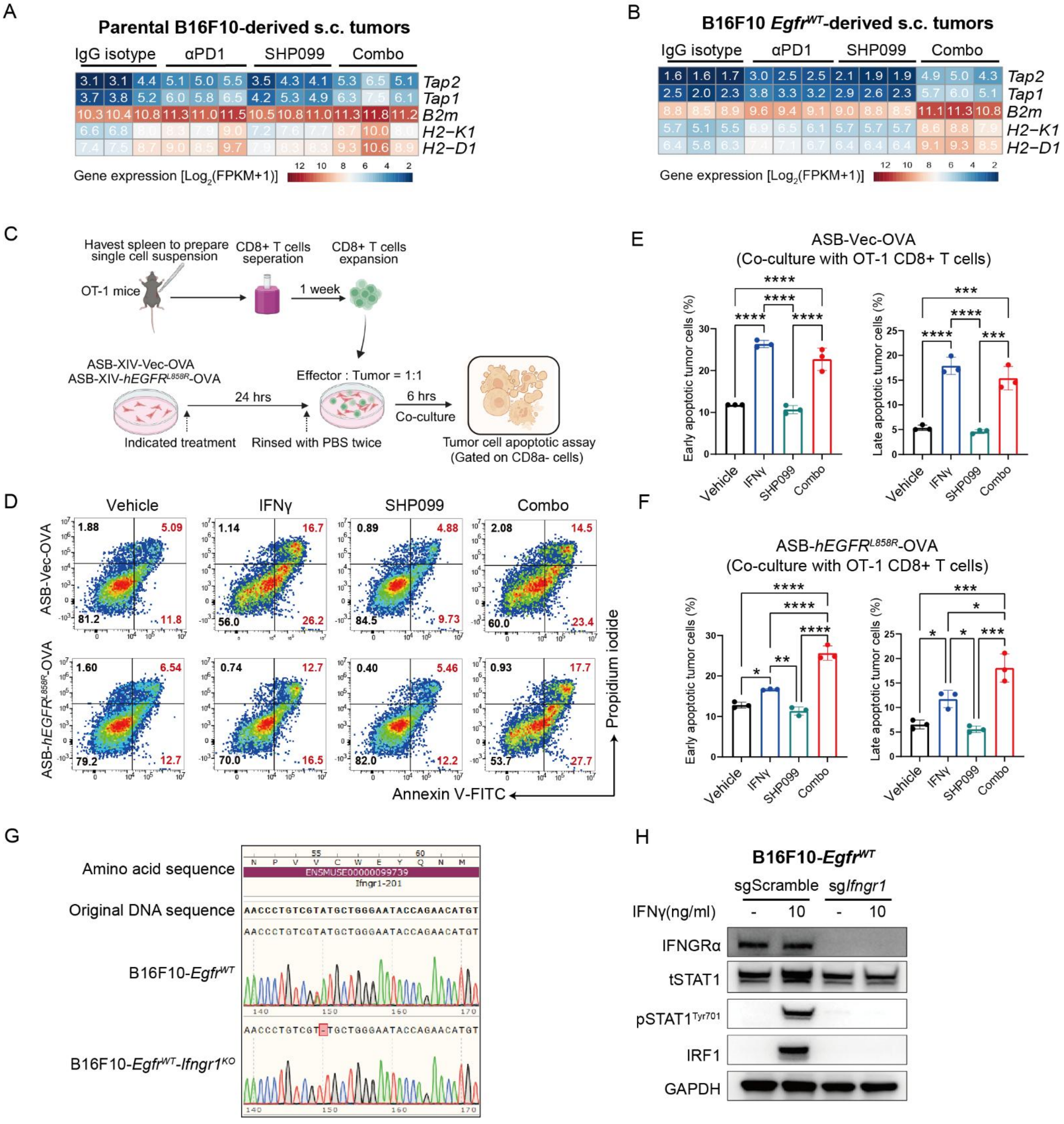

Supplemental figure 9. SHP2 inhibition restores antigen processing and presentation specifically in EGFR-activated tumor models. (A and B) RNA expression of representative genes associated with antigen processing and presentation in parental B16F10 (A) and B16F10-*Egfr*^{WT} (B) subcutaneous tumor models receiving indicated treatments. **(C)** Illustration of tumor apoptotic assay in the coculture system of OT-1 T cells and ASB-XIV-OVA cell models. ASB-XIV-Vec-OVA and ASB-XIV-*EGFR*^{L858R}-OVA cells were treated by vehicle control, mouse IFN-γ 5 ng/ml without or with SHP099 20uM for 24 hours prior to 6-hour coculture with OT-1 T cells. **(D)**

Representative fluorescence-activated cell sorting (FACS) analysis of tumor apoptotic assays. **(E and F)** Early and late apoptotic tumor cells were quantified and compared between groups in ASB-XIV-Vec-OVA (F) and ASB-XIV-*EGFR*^{L858R}-OVA (G) cell models. **(G and H)** Sanger sequencing (G) and western blot analysis (H) confirmed the successful gene editing and loss of function of *Ifngr1* in B16F10-*Egfr*^{WT} cells. All data are presented as mean±SD. Statistical significance determined by one-way ANOVA with Tukey's multiple comparison test. * $p<0.05$, ** $p<0.01$, *** $p<0.001$, **** $p<0.0001$.

Supplemental references

1. Ran FA, et al. Double nicking by RNA-guided CRISPR Cas9 for enhanced genome editing specificity. *Cell.* 2013; 154(6): 1380-9.

2. Ran FA, et al. Genome engineering using the CRISPR-Cas9 system. *Nat Protoc.* 2013; 8(11): 2281-308.

3. Cheng C, et al. NPRC deletion mitigated atherosclerosis by inhibiting oxidative stress, inflammation and apoptosis in ApoE knockout mice. *Signal Transduct Target Ther.* 2023; 8(1): 290.

4. Subramanian A, et al. Gene set enrichment analysis: a knowledge-based approach for interpreting genome-wide expression profiles. *Proc Natl Acad Sci U S A.* 2005; 102(43): 15545-50.

5. Miao YR, et al. ImmuCellAI-mouse: a tool for comprehensive prediction of mouse immune cell abundance and immune microenvironment depiction. *Bioinformatics.* 2022; 38(3): 785-91.

6. Jia Y, et al. Impact of EGFR-TKIs combined with PD-L1 antibody on the lung tissue of EGFR-driven tumor-bearing mice. *Lung Cancer.* 2019;137:85-93.

7. Ru B, et al. TISIDB: an integrated repository portal for tumor-immune system interactions. *Bioinformatics.* 2019; 35(20): 4200-02.

8. Li T, et al. TIMER2.0 for analysis of tumor-infiltrating immune cells. *Nucleic Acids Res.* 2020; 48(W1): W509-W14.

9. Saltz J, et al. Spatial Organization and Molecular Correlation of Tumor-Infiltrating Lymphocytes Using Deep Learning on Pathology Images. *Cell Rep.* 2018; 23(1): 181-93 e7.
