# Supplementary material for: EGFR activation disrupts immunotherapy response via SHP2-mediated suppression of tumor-intrinsic response to IFN-γ
Source: J Clin Invest. 2026 Jan 15;136(5):e194377. doi: 10.1172/JCI194377 (PMC12948428; doi:10.1172/JCI194377)

Supplemental Information-Full unedited western blot

EGFR activation disrupts immunotherapy response via SHP2-mediated suppression of tumor-intrinsic response to IFN-γ

Wei-Tao Zhuang, Lan-Lan Pang, Li-Yang Hu, Jun Liao, Jian-Hua Zhan, Ting Li, Ri-Xin Chen, Jia-Ni Zheng, An-Lin Li, Wen-Yan Yu, Tian-Qin Mao, Liang Chen, Yu-Jian Huang, Shao-Dong Hong, Jing Li, Jun-Han Wu, Yi-Ming Zeng, Meng-Juan Yang, Hai-Qing Zeng, Ya-Xiong Zhang, Li Zhang, Wen-Feng Fang*

*Lead Correspondence: fangwf@sysucc.org.cn

Full unedited western blot. Red boxes indicate the images used in figure 1

Figure 1E

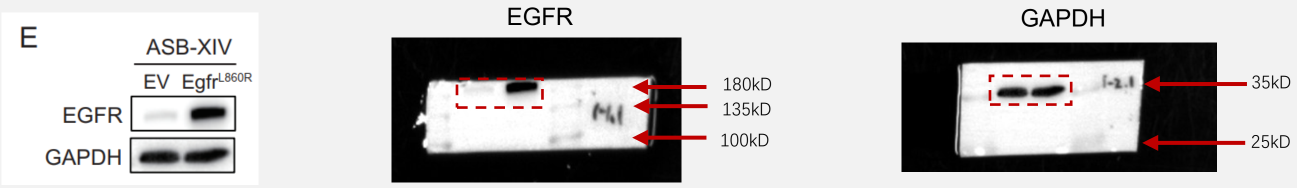

Figure 1G; Figure 1I

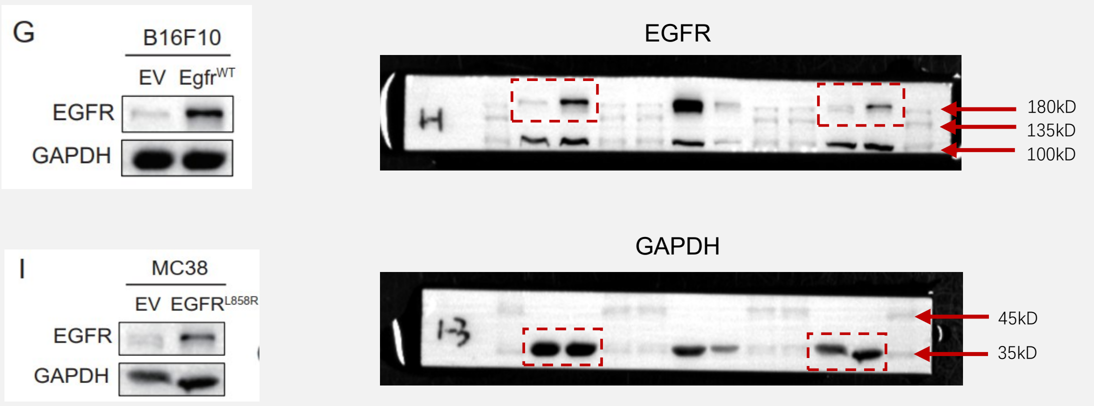

Full unedited western blot. Red boxes indicate the images used in figure 2

Figure 2A

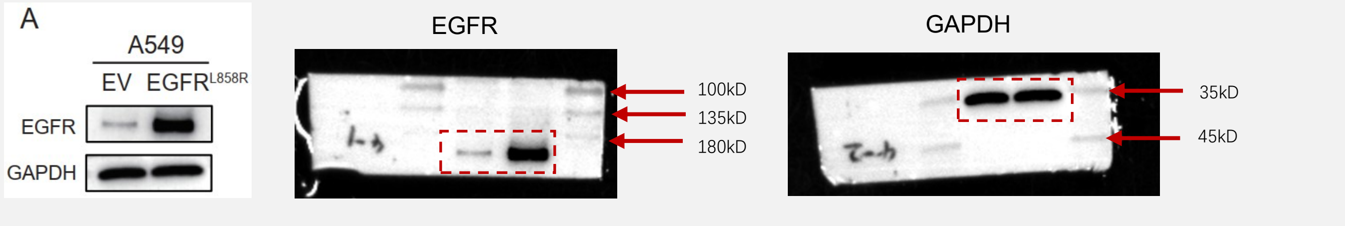

Figure 2I

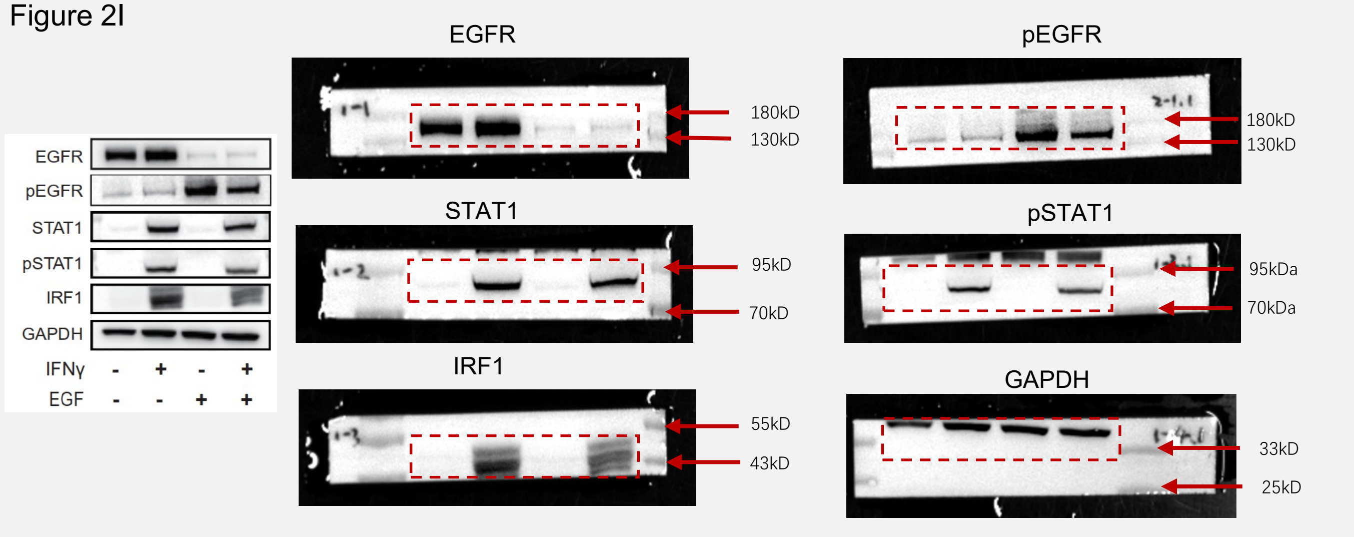

Figure 2J

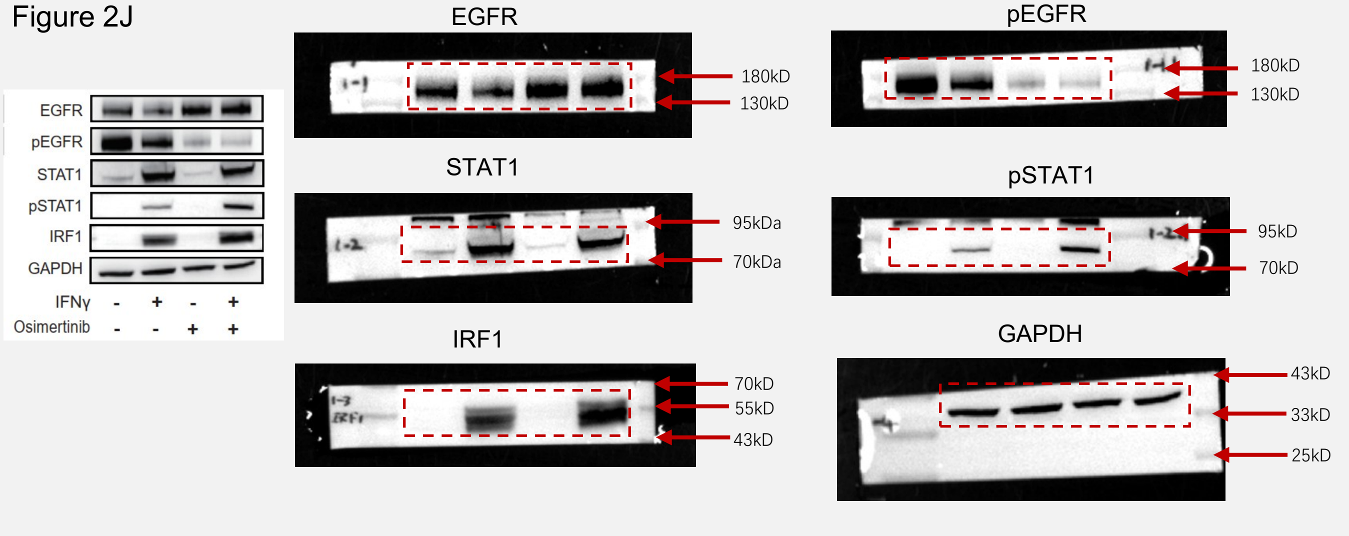

Full unedited western blot. Red boxes indicate the images used in figure 5

Figure 5A

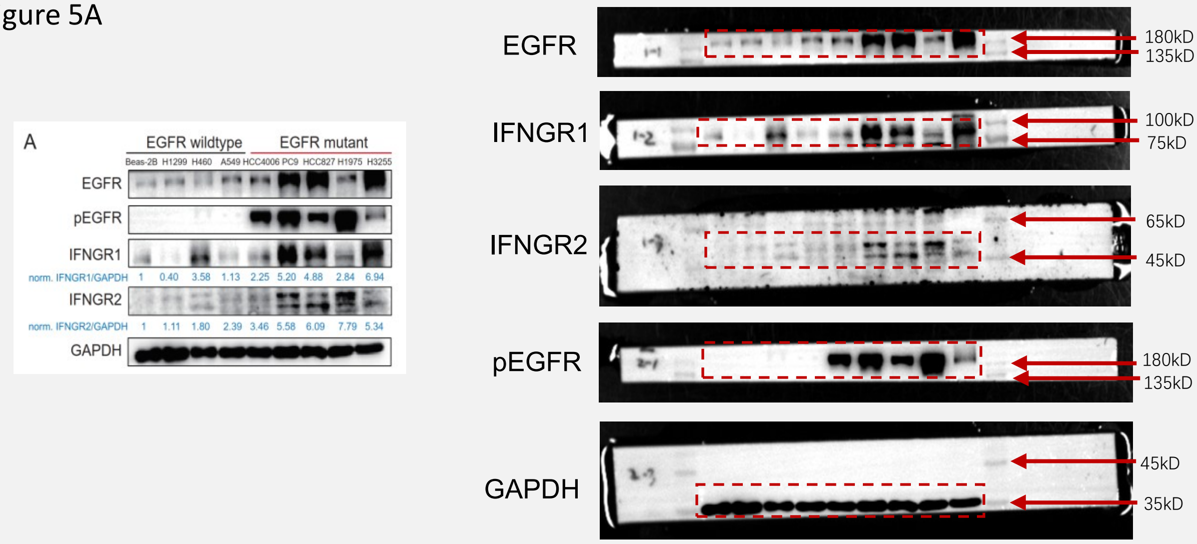

Figure 5F

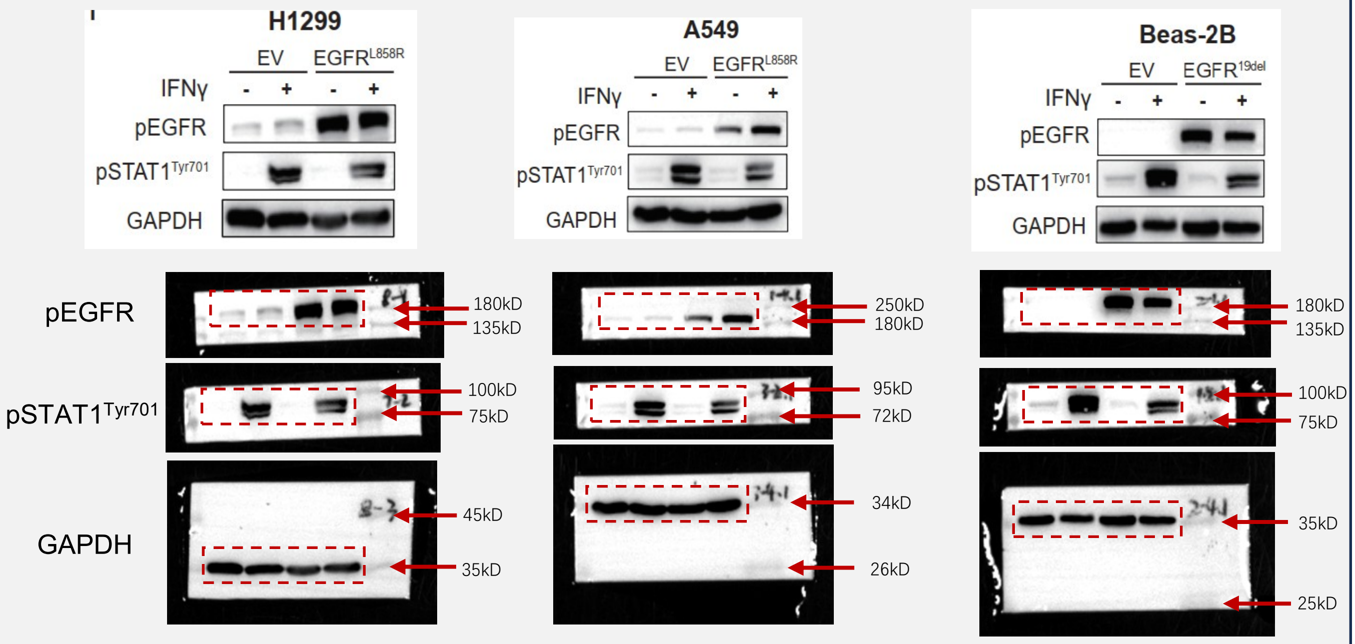

Full unedited western blot. Red boxes indicate the images used in figure 5

Figure 5D (left)

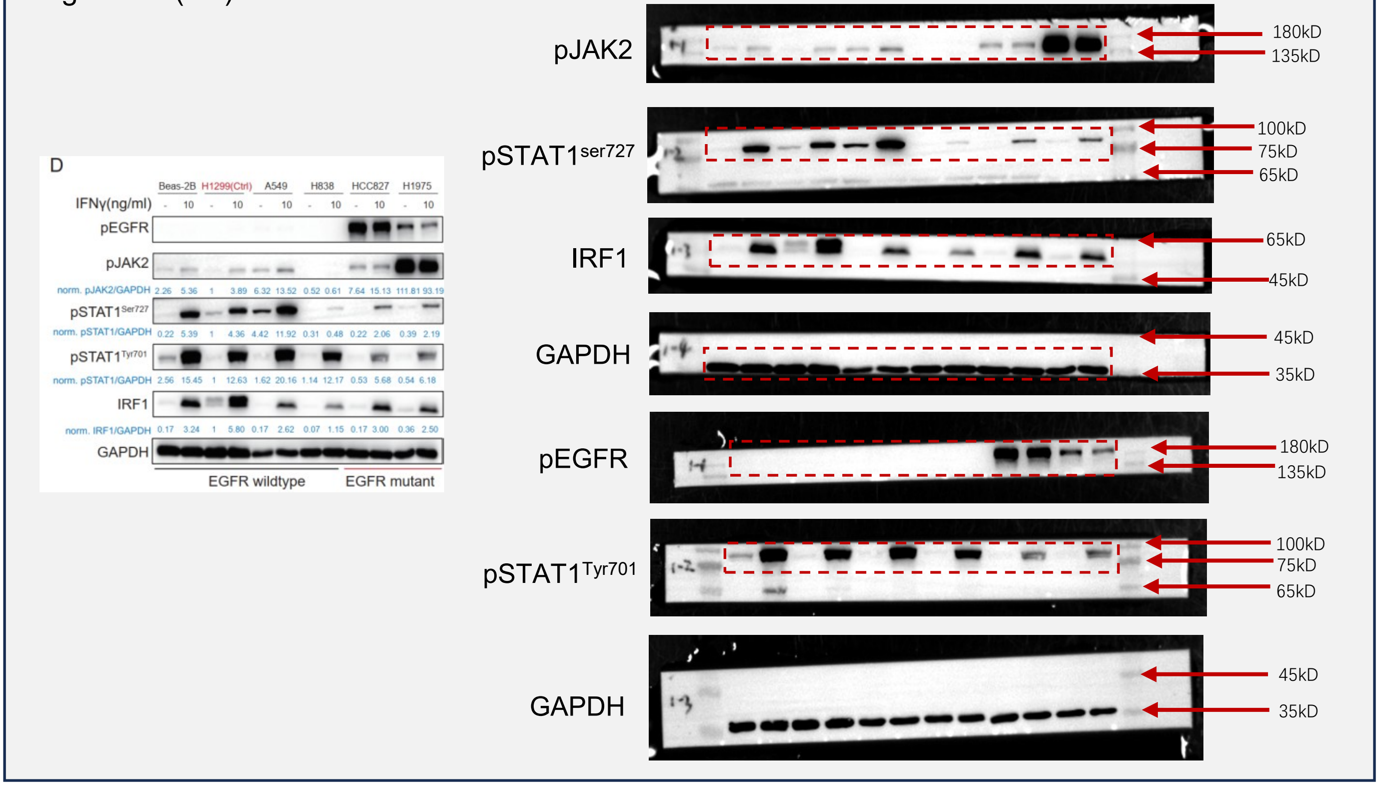

Figure 5D (right)

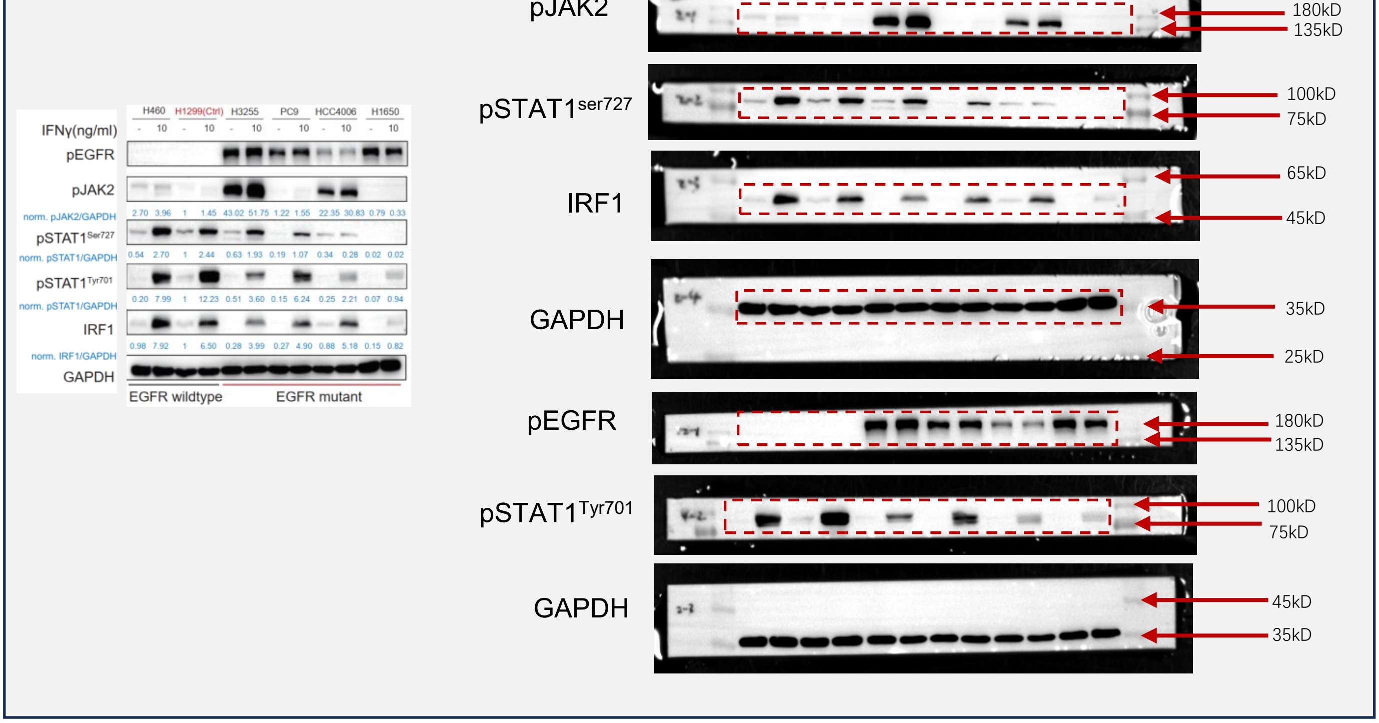

Full unedited western blot. Red boxes indicate the images used in figure 5

Figure 5H

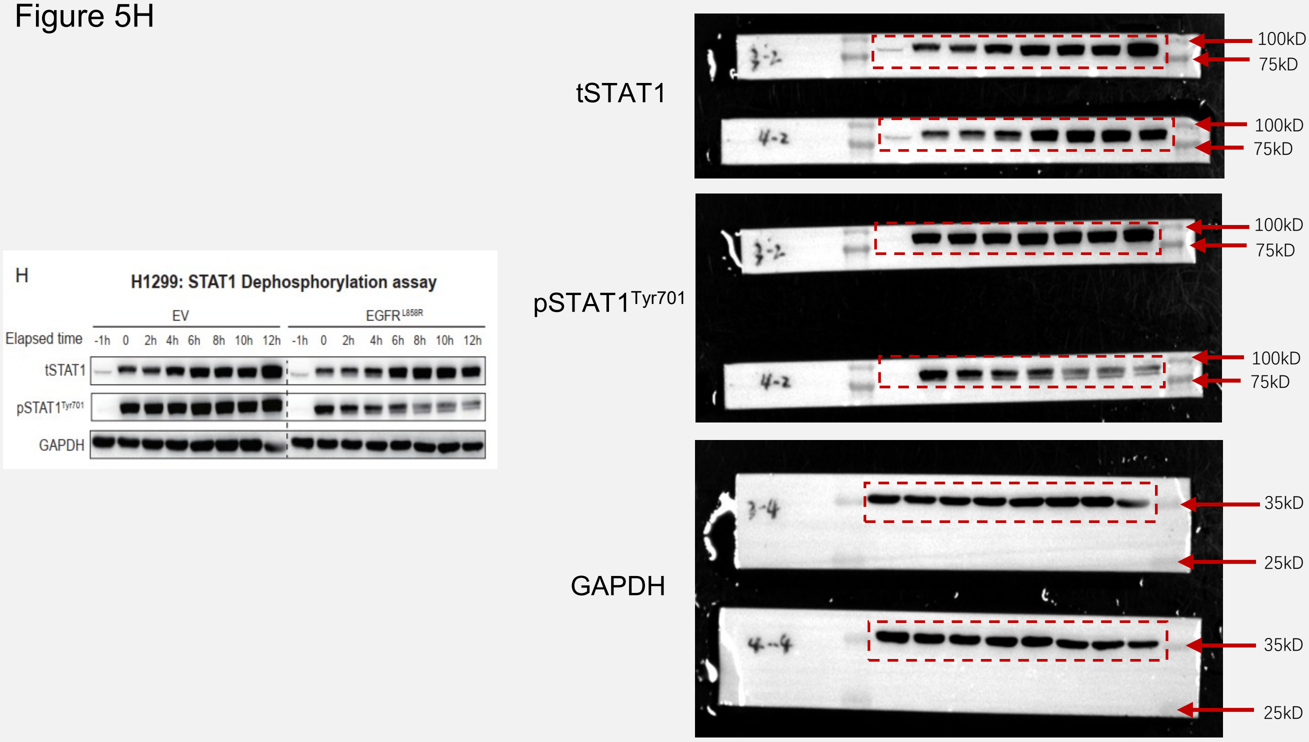

Figure 5K

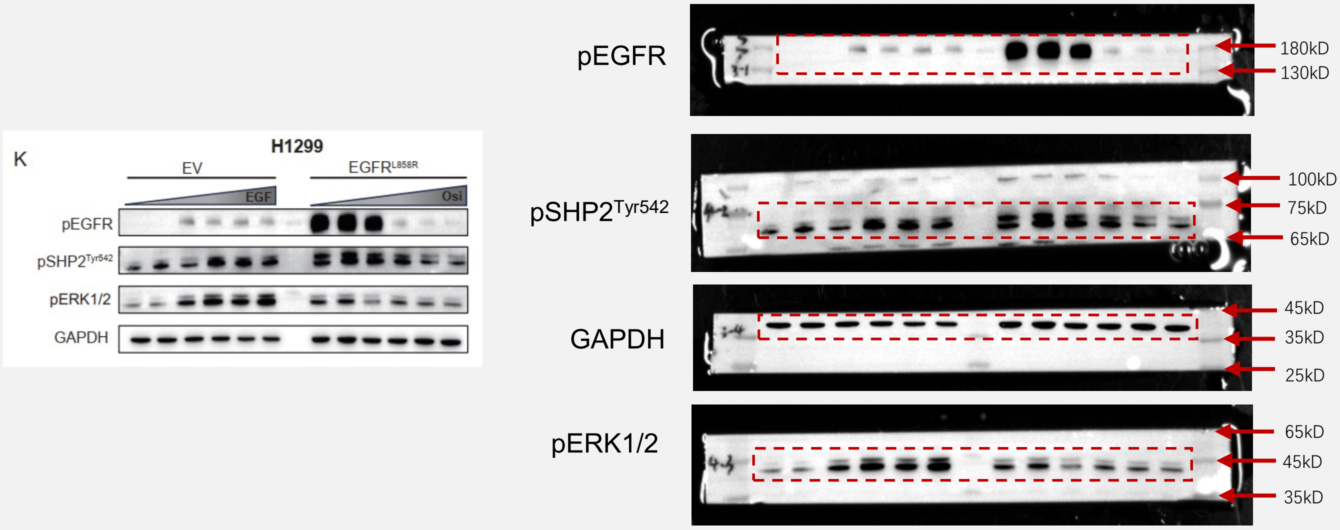

Full unedited western blot. Red boxes indicate the images used in figure 6

Figure 6A

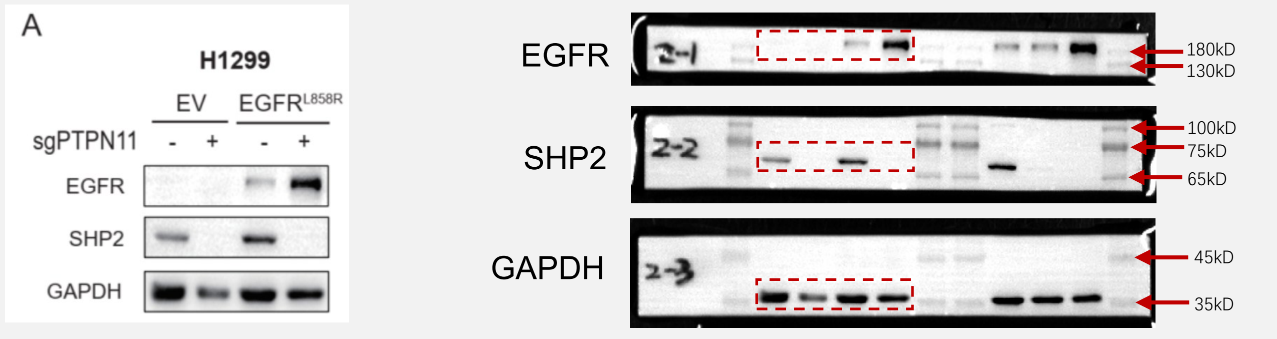

Figure 6H

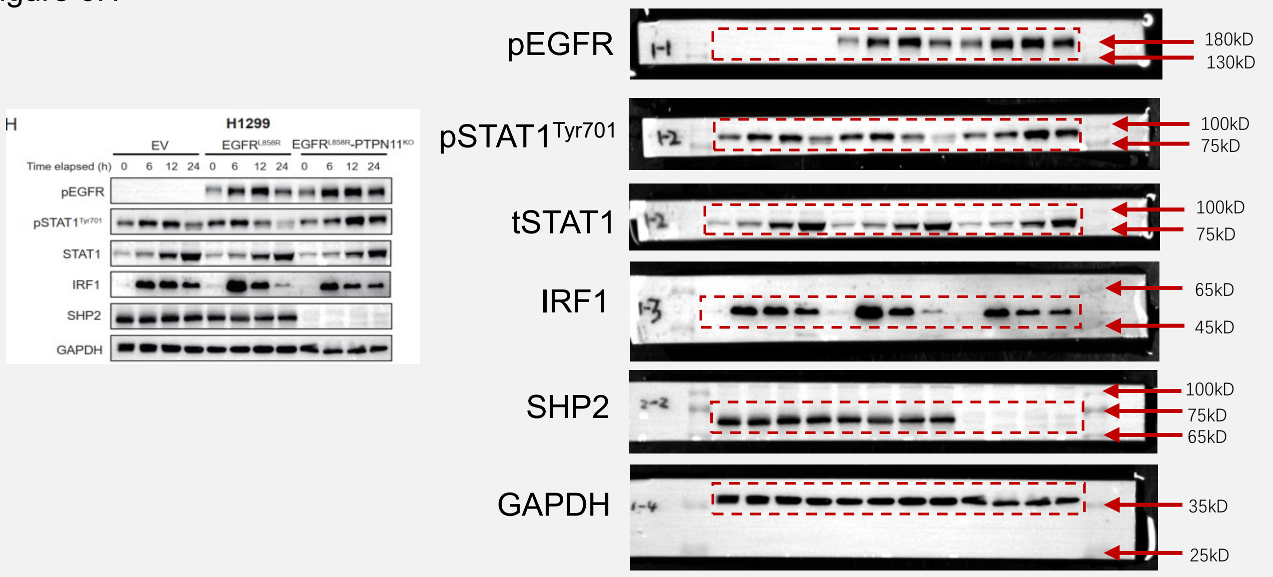

Figure 6I

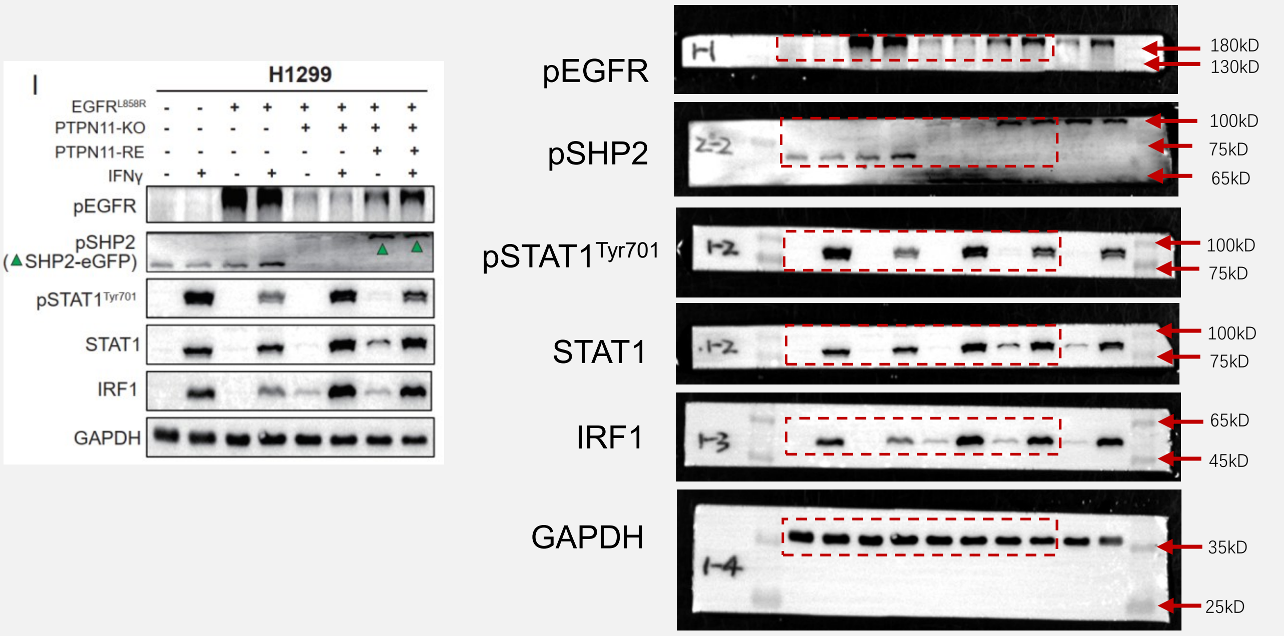

Full unedited western blot. Red boxes indicate the images used in Supplemental figure 6

Supplemental Figure 6C

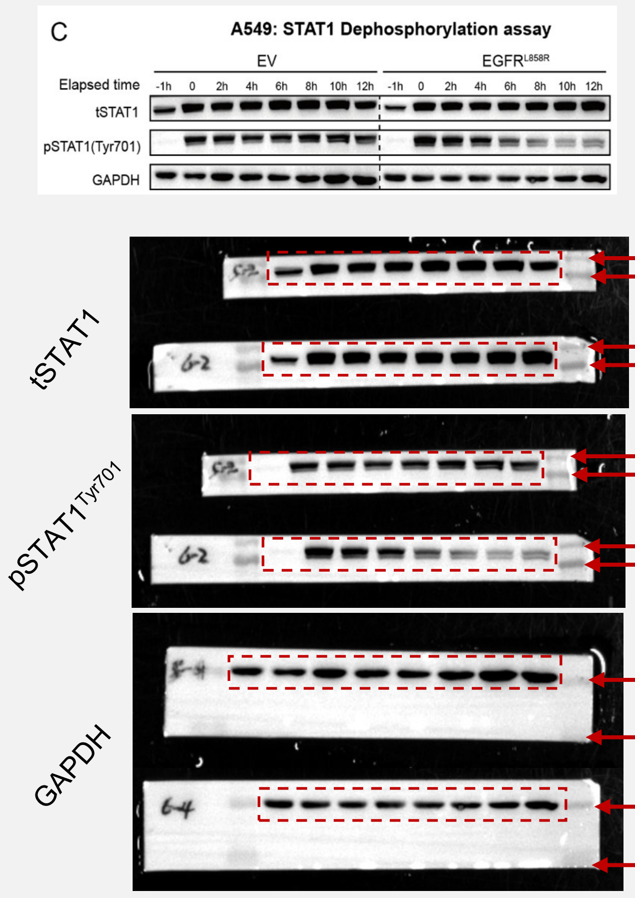

Supplemental Figure 6D

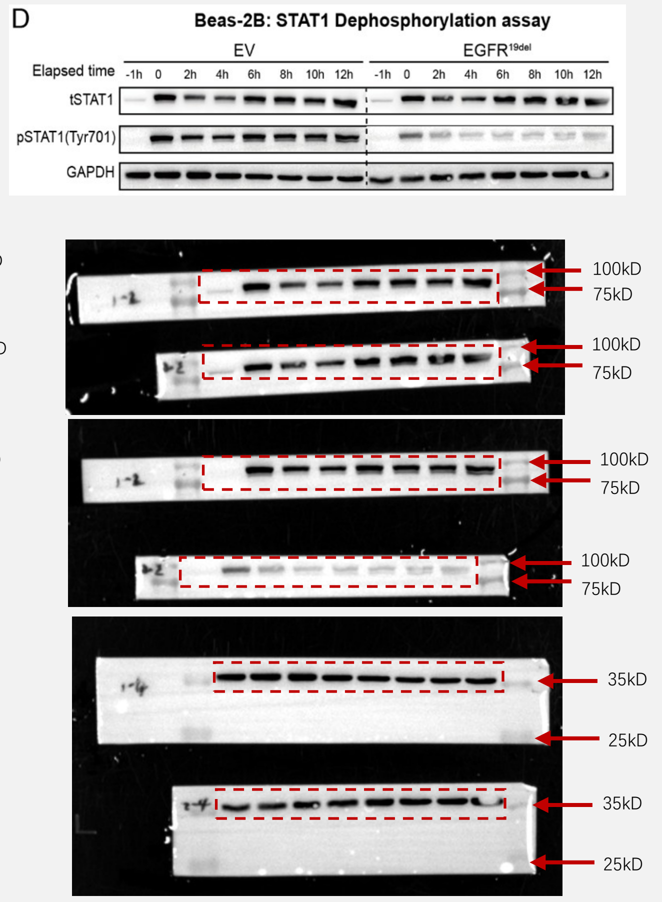

Supplemental Figure 6E

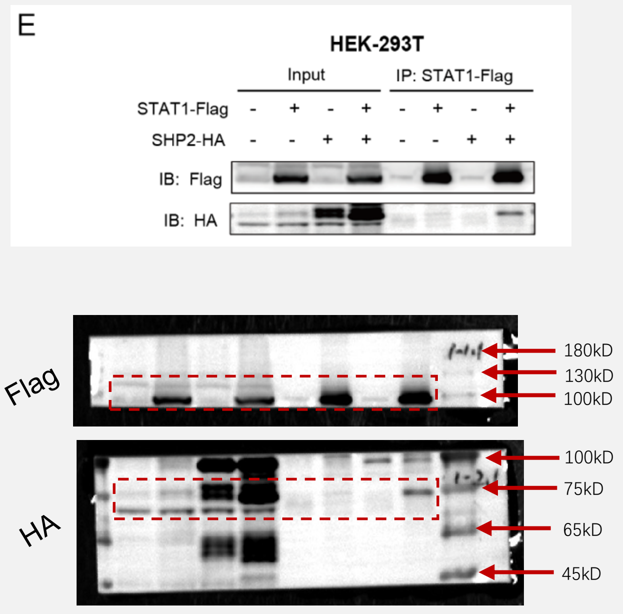

Supplemental Figure 6F

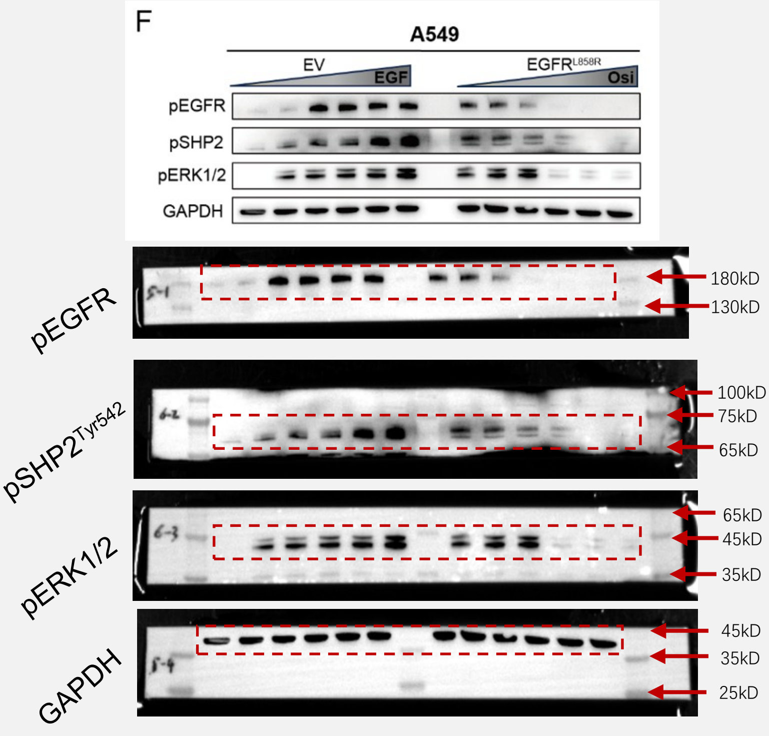

Full unedited western blot. Red boxes indicate the images used in Supplemental figure 9

Supplemental Figure 9H

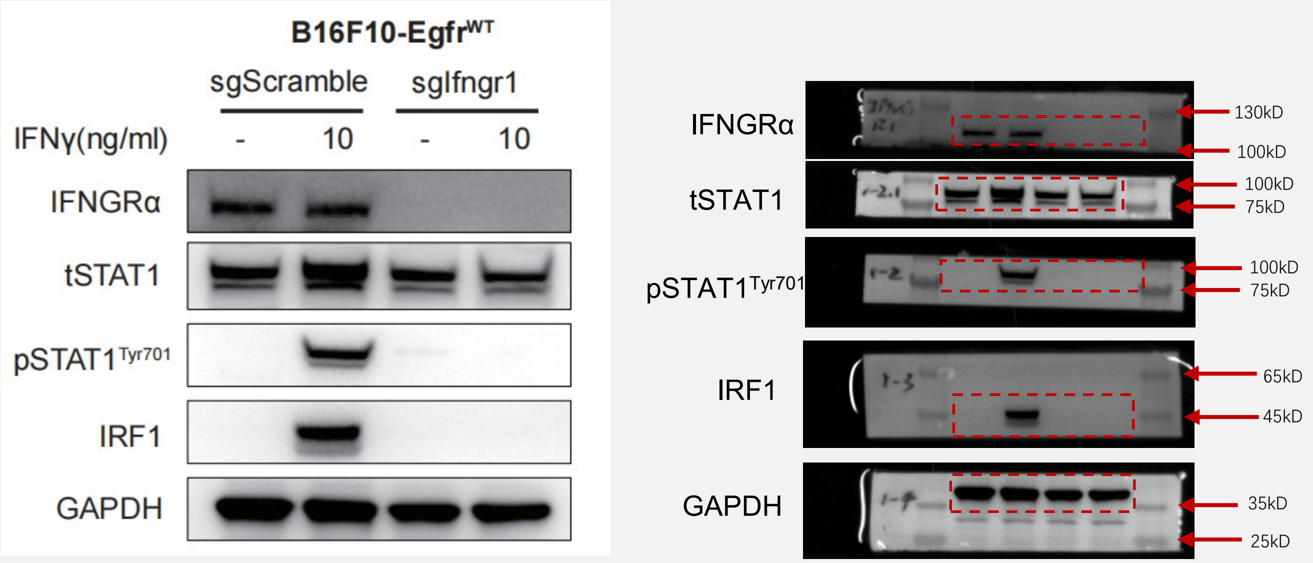

Supplement: Unedited blot and gel images [file jci-136-194377-s011.pdf]
